# The role of context in elucidating drivers of animal movement

**DOI:** 10.1002/ece3.9128

**Published:** 2022-07-24

**Authors:** Nicolas Lubitz, Michael Bradley, Marcus Sheaves, Neil Hammerschlag, Ryan Daly, Adam Barnett

**Affiliations:** ^1^ College of Science and Engineering James Cook University Townsville Queensland Australia; ^2^ Marine Data Technology Hub College of Science and Engineering James Cook University Townsville Queensland Australia; ^3^ Rosenstiel School of Marine and Atmospheric Science University of Miami Miami Florida USA; ^4^ Oceanographic Research Institute Durban South Africa; ^5^ South African Institute for Aquatic Biodiversity (SAIAB) Makhanda South Africa

**Keywords:** animal movement, birds, context, elasmobranchs, environmental change, intra‐specific variability, migration, Movement drivers, tagging bias

## Abstract

Despite its consequences for ecological processes and population dynamics, intra‐specific variability is frequently overlooked in animal movement studies. Consequently, the necessary resolution to reveal drivers of individual movement decisions is often lost as animal movement data are aggregated to infer average or population patterns. Thus, an empirical understanding of why a given movement pattern occurs remains patchy for many taxa, especially in marine systems. Nonetheless, movement is often rationalized as being driven by basic life history requirements, such as acquiring energy (feeding), reproduction, predator‐avoidance, and remaining in suitable environmental conditions. However, these life history requirements are central to every individual within a species and thus do not sufficiently account for the high intra‐specific variability in movement behavior and hence fail to fully explain the occurrence of multiple movement strategies within a species. Animal movement appears highly context dependent as, for example, within the same location, the behavior of both resident and migratory individuals is driven by life history requirements, such as feeding or reproduction, however different movement strategies are utilized to fulfill them. A systematic taxa‐wide approach that, instead of averaging population patterns, incorporates and utilizes intra‐specific variability to enable predictions as to which movement patterns can be expected under a certain context, is needed. Here, we use intra‐specific variability in elasmobranchs as a case study to introduce a stepwise approach for studying animal movement drivers that is based on a context‐dependence framework. We examine relevant literature to illustrate how this context‐focused approach can aid in reliably identifying drivers of a specific movement pattern. Ultimately, incorporating behavioral variability in the study of movement drivers can assist in making predictions about behavioral responses to environmental change, overcoming tagging biases, and establishing more efficient conservation measures.

## Graphical abstract

In this article, we use intra‐specific variability in elasmobranchs as a case study to introduce a stepwise approach for studying animal movement drivers that is based on a context‐dependence framework. Ultimately, incorporating behavioural variability in the study of movement drivers can assist in making predictions about behavioural responses to environmental change, overcoming tagging biases and establishing more efficient conservation measures.

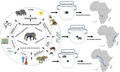

## 1 INTRODUCTION

Animal movement is often defined as the change of an individual's location over time (Nathan et al., 2008). This can include large‐scale migrations, nomadic behavior, small‐scale displacements, and range residency. Thus, in this article we consider movement over varying spatial and temporal scales. Movement enables mobile organisms to fulfill their life history requirements across time and space and has important implications for species evolution and ecology (Bauer & Hoye, 2014; Kingsolver et al., 2002). Large‐scale movement, for example, is often seen as costly, requiring substantial energetic investment, and should in theory only persist if it is beneficial by increasing overall fitness (Dingle, 2014). Movement enables gene flow through genetic connectivity between populations and sub‐populations (e.g., Rizzo & Schulte, 2009). Furthermore, mobile animals connect disparate habitats through the transfer of nutrients, energy, parasites and propagules, and the translocation of trophic processes (Bauer & Hoye, 2014). Far‐ranging predators, for example, can have strong seasonal influences on prey animals either through direct predation or risk effects (Barnett & Semmens, 2012; Hammerschlag et al., 2019; Heithaus et al., 2012).

Historically, the study of movement ecology has focused on the “where and when”—aspect of animal movement. This was the first step in studying patterns such as residency, seasonality, and migration patterns which has demonstrated that intra‐specific variability in movement behavior is common in all major taxonomic groups (Barnett et al., 2011; Block et al., 2011; Chambert et al., 2015; Chapman et al., 2012; Flack et al., 2016; Geijer et al., 2016; Hatase et al., 2010; Joly et al., 2019; Papastamatiou et al., 2013). Intra‐specific variability adds substantial complexity to animal movement studies, as it can occur in many different forms. This includes overall movement tendency, from resident to migratory behavior, variability in timing, and distance of movement, as well as destinaton (Chapman et al., 2011; Shaw, 2020). Additionally, it can occur at multiple scales, from differences in movement behavior among individuals in different geographic locations, among individuals within the same location, and within individuals over time (Chapman et al., 2011; Shaw, 2020).

Despite its consequences for ecological processes and population dynamics, intra‐specific variability is frequently overlooked in animal movement studies (Shaw, 2020). The necessary resolution to reveal drivers of individual movement decisions is often lost as animal movement data are aggregated to infer average or population patterns (Holyoak et al., 2008; Shaw, 2020). Thus, an empirical understanding of why a given movement pattern occurs remains patchy for many taxa, especially in marine systems (but see e.g., Humphries et al., 2010, 2012, Sims et al., 2012).

Movement drivers are often rationalized as being driven by basic life history requirements or intrinsic needs, sometimes referred to as “ultimate drivers” (Mayr, 1963; Scott‐Phillips et al., 2011; Shaw, 2016). These requirements reflect critical constraints, such as acquiring energy (feeding), reproduction, predator‐avoidance, and remaining within physiological optima, which are central to every individual within a species (Shaw, 2016). Thus, they alone do not sufficiently account for intra‐specific variability and the occurrence of multiple movement strategies within a species. For example, the behavior of both, resident and migratory individuals, is driven by life history requirements. Yet, different movement strategies are utilized to fulfill them. Therefore, animal movement appears context‐dependent (Bradley et al., 2019; Humphries et al., 2010).

Context‐dependence has long been acknowledged in ecological studies, however, often without establishing or defining the “context” under which ecological relationships and environmental processes occur (sensu Bradley et al., 2019). This step is increasingly appreciated as valuable in quantifying and understanding variability in the natural world (Bradley et al., 2020). For example, context‐dependence has been used to understand the drivers of variability in habitat use of inshore fish assemblages and foraging patterns in marine predators (Bradley et al., 2019, 2020; Humphries et al., 2010). However, a systematic taxa‐wide approach that, instead of averaging population patterns, incorporates intra‐specific variability to determine the context that can be reliably related to a specific movement pattern, is missing.

In this study, we describe a context‐focused approach for animal movement data and evaluate the extent to which it can improve our understanding of animal movement decisions. Such an approach is applicable to any taxa with variability in movement behavior. However, here we focus our discourse on sharks and rays (elasmobranchs) as a case study. Elasmobranchs represent a diverse and globally distributed group containing highly mobile species that exhibit large‐scale movements (up to 18,000 km, Queiroz et al., 2019) but also exhibit significant intra‐specific variation in movement patterns (e.g., Barnett et al., 2011; Espinoza et al., 2016; Papastamatiou et al., 2013; Vaudo et al., 2017). The objectives of this study were to assess how context shapes individual movement decisions by influencing behavioral traits linked to life history requirements and apply a context‐focused approach to elasmobranchs as a case study to evaluate the utility of the approach by examining its benefits and applications to animal movement ecology.

## 2 THE RELATIONSHIP BETWEEN CONTEXT AND MOVEMENT

**FIGURE 1:**
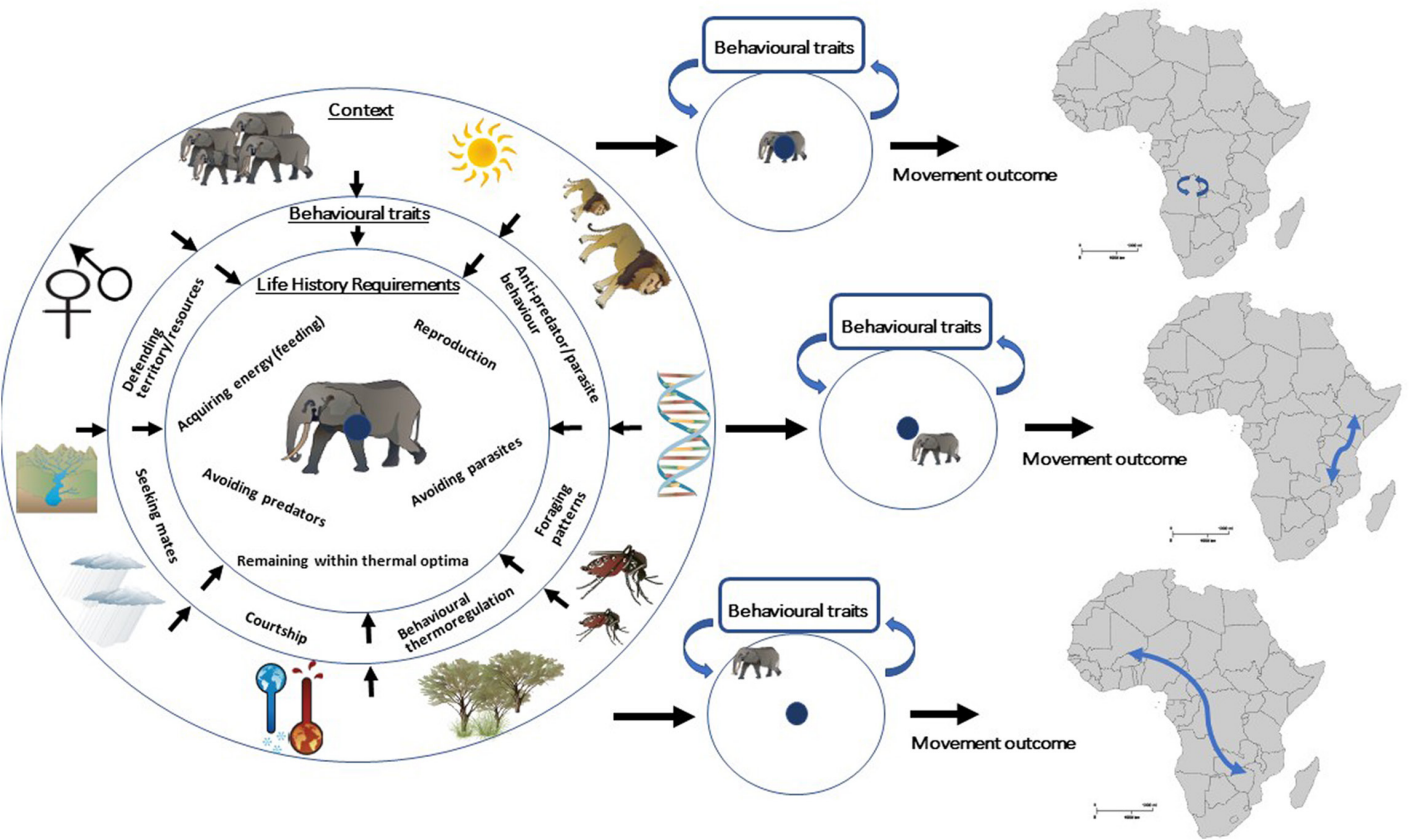
An individual, represented by the elephant in center of each circle, is subjected to constraints linked to life history requirements. Behavioral traits are expressed to fulfill life history requirements within a given context. The context is the sum of contextual factors. Depending on the context, deviation from a theoretical optimum (blue dots) where all life history requirements can be fulfilled within one geographical location at all times may occur. Behavioral adjustments then result in a particular movement decision. For each individual, this is based on an interaction of the environmental, ecological, and individual context in a given time and place. Determined by which life history requirements an individual can no longer fulfill in the current location at that time, that is, the magnitude and direction of shift from optimum (blue dot), differences in timing, direction, and distance between individuals will arise. Hypothetical examples below illustrate different levels of deviation from theoretical optimum caused by differing contextual factors that may give rise to a particular movement pattern.

The overall behavior of animals is driven by fulfilling life history requirements, such as acquiring energy, avoiding predators and parasites as well as reproducing, which are critical to an individual's fitness (Figure 1). Associated with fulfilling those requirements are certain physiological and behavioral traits. Examples are evolution of jaw structures for specific prey types, evolution of ornaments and weapons in males to increase reproductive success, seasonal migration away from unfavorable conditions, or grooming to avoid parasites (Andersson, 1982; Bels & Whishaw, 2019; Hart & Hart, 2018; Hedrick & Temeles, 1989; Somveille et al., 2015).

At any point in time an individual exists within a certain *context* which we define here as the product of a range of relatively predictable contextual factors: The *environmental context*, the *ecological context*, and the *individual context* (Box 1, Figure 1). The environmental context includes factors such as seasonal temperature changes, topography, rainfall, and current patterns. The ecological context encapsulates prey availability, prey species‐composition, levels of competition, and predation pressure, for example, and the individual context is comprised of individual body condition, sex, genetic‐makeup, and reproductive behavior for example (Box 1). These contextual factors broadly align with what is often referred to in the literature as “proximate drivers” (Mayr, 1963; Scott‐Phillips et al., 2011). Hereafter, “driver” refers to one or multiple contextual factors producing a certain movement *outcome* (Box 1).

BOX 1 **Definitions of terms**

**Table:** 

Contextual factor	A factor that drives a process or an individual's behavior. It may vary across time, space, and individuals
Outcome	The way a process or behavior, which is driven by contextual factors, is expressed (e.g., large‐scale movement, small‐scale movement or residency)
Context	Sum of contextual factors producing an outcome
System	Study systems under investigation: For example, geographically distinct populations of a species or animals tagged in differing geographic locations, demographic groups (e.g., Males vs. females, mature vs. immature), individuals at range limits, vs. individuals within the center of distribution
Environmental context (external)	Includes all abiotic factors encountered by an individual. Examples are temperature changes, topography, tides and currents, rainfall and substrate, nutrient levels.
Ecological context (external)	Includes all biotic factors encountered by an individual. Examples are food availability, species‐composition and ‐interactions (predator–prey, competition, prevalence of parasites).
Individual context (internal)	Includes all factors specific to an individual. Examples are sex, reproductive status, body condition, energetic state, health, individual feeding specialization, body‐ and appendage size, ontogeny, as well as genetic make‐up.

The interaction of life history requirements with the context in which the individual exists shapes the way physiological and behavioral traits are expressed to increase fitness (Figure 1). Possible intra‐specific differences in trait expression can take place on evolutionary time scales through genetic adaptions. For example, according to Bergmann's rule, individuals of higher body mass occur in colder climates compared to conspecifics occupying warmer areas (Bergmann, 1848; Meiri & Dayan, 2003). Alternatively, trait expression can be flexible and change over an individual's lifetime as a response to changing conditions. For instance, gentoo penguins (*Pygoscelis papua*) change their foraging patterns and diet with increased competition (Ratcliffe et al., 2018), while in the red junglefowl (*Gallus gallus*) male reproductive behavior changes with the level of intensity of sexual competition (Cornwallis & Birkhead, 2008).

Animal movement is also aimed at improving fitness (Dingle, 2014). This means, when one or more life history requirements can no longer be fulfilled in the current location, behavioral traits associated with the life history requirement are combined with movement (Figure 1). If movement is necessary, it is determined by contextual factors, which also drive the resulting pattern of movement (Humphries et al., 2010, 2012). For example, If the context in which an animal exists is consistently optimal in time and space, large‐scale movement away from an area would likely not be necessary (Figure 1). However, the context an individual is subjected to is the result of a complex interaction of a variety of contextual factors that can vary across time and space. For example, elk (*Cervus elaphus*) change between resident and migratory behavior driven by an interaction of winter severity, wolf abundance, and elk density (Eggeman et al., 2016). Thus, context is tightly linked to intra‐specific variability in movement behavior. It is this dynamic interplay between life history requirements and their associated behavioral traits with contextual factors that drives individual movement decisions (Figure 1, Shaw, 2020).

## 3 THE CONTEXT‐FOCUSED APPROACH

With the aim of unifying movement studies under a common conceptual framework, Nathan et al. (2008) proposed a movement ecology paradigm based on principle components of animal and plant movement. This includes how the organism moves, when and toward what destination the movement occurs, why the organism moves, and the interaction of these components. However, one of the key challenges in movement ecology remains the identification of specific external and internal factors driving the movement of individuals, especially in light of complexity introduced by intra‐specific variability (Holyoak et al., 2008; Nathan et al., 2008; Shaw, 2020). Although previously proposed frameworks are centerd around individuals and aim to at least recognize intra‐specific variability, approaches to deal with variation and utilize it are missing (Holyoak et al., 2008, Shaw, 2020, but see e.g., Humphries et al., 2010). Specifically, we lack a predictable means to determine which movement pattern can be expected given a combination of contextual factors.

By focusing on relatively predictable and measurable contextual factors (Box 1) a context‐focused approach constitutes a heuristic tool. Ecological relationships and phenomena, such as movement behavior are often complex. By trying to remove complexity through averaging data and inferring population patterns a thorough understanding of ecological relationships and behaviors and how they might be affected by future change is hindered. Thus, in terms of animal movement, for example, the aim of a context‐focused approach is to define the context in which certain patterns occur and simplify complexity while retaining enough resolution to link specific contextual factors to individual movement decisions.

**FIGURE 2:**
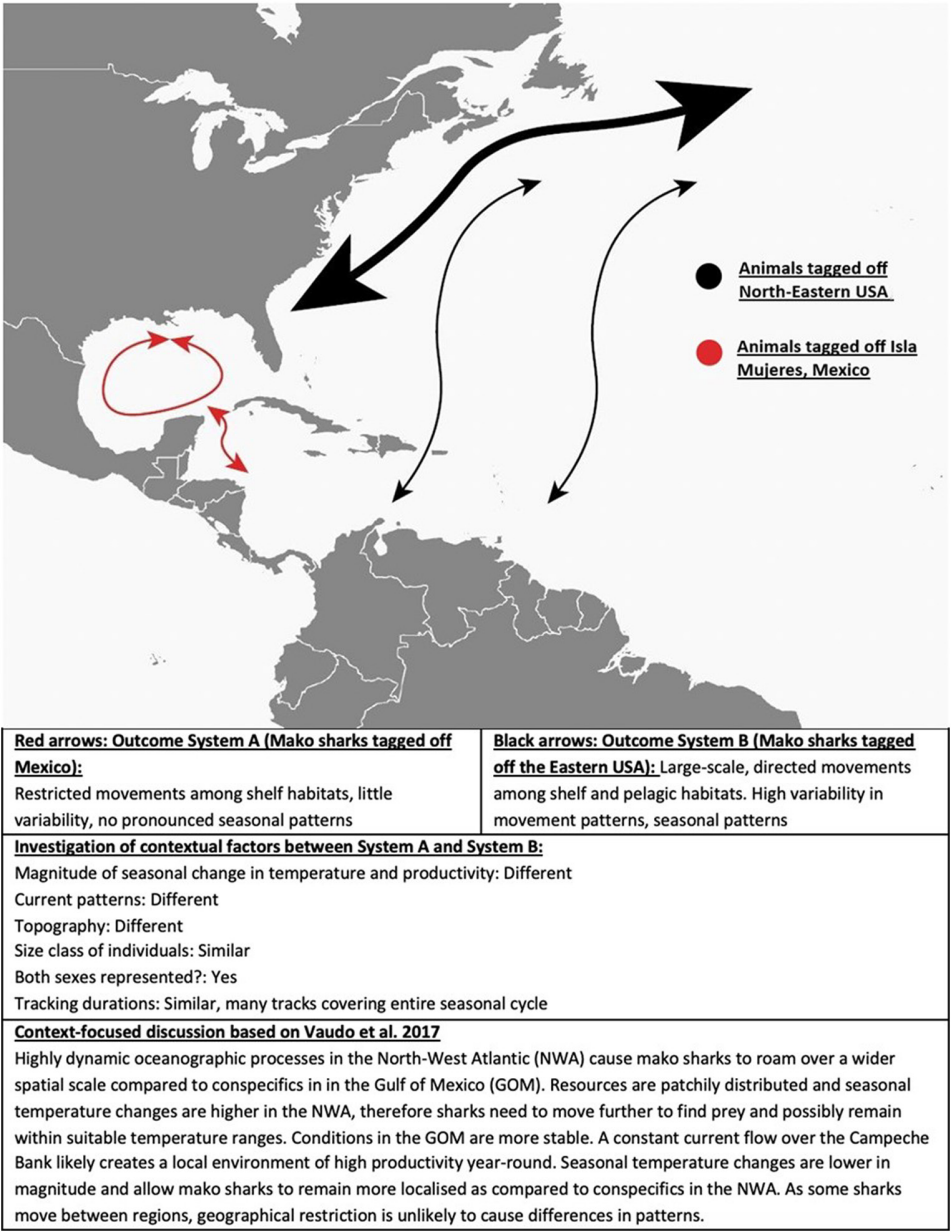
Simplified context‐approach (Steps 2 and 3, based on Vaudo et al., 2017)

Overall, this approach is based on comparing movement patterns in two or more study systems. A study system can consist of conspecifics in different geographical locations or animals of different sex or size classes, for example (Figure 2). The goal is to identify differences in contextual factors among study systems and relate those back to potential differences in observed movement patterns. Contextual factors that are similar among study systems are unlikely to drive differences in movement patterns, while contextual factors that differ can then be further investigated as to how those factors may shape observed movement patterns (Figure 2).

To achieve this, the approach employs a stepwise process that, first, begins with compiling existing knowledge of life history requirements and associated movement patterns of the species/study system considered while establishing the context in which the study systems exist. Second, it investigates the extent to which intra‐specific variability of movement behavior is evident within the species/study system, such as in geographically distinct groups of individuals or individuals within a population (such as males and females, adults and sub‐adults). Third, this variability is used to identify spatio‐temporal and individual differences in contextual factors that may be responsible for the variability in movement patterns (Figure 2). This aids in revealing how context shapes patterns of intra‐specific variability and ultimately individual movement decisions within each study system.

## 4 STEP 1: COMPILING CURRENT KNOWLEDGE OF LIFE HISTORY REQUIREMENTS AND ASSOCIATED MOVEMENT PATTERNS FOR STUDY SPECIES

As a first step, in Table 1 we compiled current knowledge of life history requirements and associated movement patterns for six example species (for an extended version of Table 1 and full discussions of movement patterns and movement drivers for 18 species see Appendix S1). Despite an increase in information on spatio‐temporal aspects of elasmobranch movement over the last decades, and numerous hypotheses as to specific factors shaping given movement patterns, the underlying mechanisms remain, for the most part, unknown. This lack of understanding is likely due to several reasons. First is the practical difficulty in observing behaviors of marine species that occur at low densities and can move over large spatial scales. Second, often studies investigate the spatio‐temporal patterns of movement without testing explicitly for drivers and then hypothesize possible movement drivers, posthoc (Hammerschlag et al., 2011; Papastamatiou & Lowe, 2012). Few hypothesis‐driven studies investigate specific contextual factors driving movement a priori (Table S1). Basic life history requirements, for example, are commonly proposed as driving overall movement behavior within species without explicitly considering the role of contextual factors shaping variability in movement (Table S1).

**TABLE 1:** Examples of species for which movement patterns related to fulfilling life history requirements have been discussed in the literature (For a more extended version of Table 2, covering 18 species, see Appendix S1).

Species	Life history requirement associated with movement	Information	Source of information	Sources
Broadnose sevengill shark (*Notorynchus cepedianus*)	Feeding, reproduction	Move into coastal areas in Tasmania following prey. Absence of smaller size‐classes and little mating behavior rules out reproduction causing movement into Tasmania. When leaving coastal areas in Winter show sex‐specific migration with males undergoing large‐scale movements up the New South Wales coast while some females remain in coastal Tasmania. In the United States, movement into estuary systems coincides with high food availability. A large female in Pacific North‐West showed movement to potential birthing and/or nursery areas.	Satellite telemetry (horizontal and vertical movements), acoustic telemetry, temperature measurements, stable isotope analysis, stomach content analysis, reproductive studies (hormone analysis + lack of mating scars), prey tracking, and investigation of spatial overlap, energetics analysis, catch rate surveys, potential nursery areas identified	Ebert (1996), Barnett, Abrantes, Stevens, Yick, et al. (2010); Barnett, Abrantes, Stevens, Bruce, and Semmens (2010); Barnett, Redd, Frusher, Stevens, and Semmens (2010); Barnett, Stevens, Frusher, and Semmens (2010), Abrantes and Barnett (2011), Barnett et al. (2011), Williams et al. (2011), Barnett and Semmens (2012), Williams et al. (2012), Awruch et al. (2014), Stehfest et al. (2014)
Bull shark (*Carcharhinus leucas*)	Feeding, reproduction, temperature	Show round‐trip migrations to and from the central Great Barrier Reef, coinciding with suspected high prey availability. Seasonal presence at low latitude sites such as Sydney Harbor, associated with food availability. Female bull sharks likely migrate into river systems to give birth. Limited data suggests female natal philopatry. Movement broadly associated with temperature changes in South Africa and Australia	Satellite telemetry (horizontal and vertical movements), acoustic telemetry, genetics, inferred prey life history from other studies, tracking prey and shark overlap, temperature measurements, identified nursery areas, stable isotope analysis, stomach content analysis, catch rates	Cliff and Dudley (1991), Carlson et al. (2010), Matich et al. (2011), Werry et al. (2011), Tillett et al. (2012), Brunnschweiler and Barnett (2013), Daly et al. (2014), Heupel et al. (2015), Lea, Humphries, et al. (2015), Espinoza et al. (2016), Glaus et al. (2019), Lee et al. (2019), Smoothey et al. (2019), Niella et al. (2020)
Reef manta *(Mobula alfredi)*	Feeding, reproduction? temperature?	Movements in Eastern Australia are associated with food availability caused by seasonal, dynamic oceanic conditions. Directional large scale‐movements evident in Western Australia. Movement in Maldives influenced by monsoons as they impact productivity. Fatty acid analysis shows diet is based on demersal zooplankton. Movement may be linked to tracking this prey type. Reproduction in relation to movement not well known. Small juveniles and young‐of‐the‐year are rarely observed. Pregnant females at aggregation sites, leave and re‐appear a few days later, seemingly nonpregnant suggesting at least small scale movements to birthing grounds. In other areas, adults, juveniles, and newborns overlap spatially, migration for parturition questionable. Temperature effects evident in Australia.	Satellite telemetry (horizontal and vertical movements), acoustic telemetry, abundance counts, and behavioral observations (photo‐ID), oceanographic conditions inferred from other studies, temperature measurements, remote sensing of environmental factors, fatty acid analysis	Marshall and Bennett (2010), Anderson et al. (2011), Jaine et al. (2012), Couturier et al. (2013), Jaine et al. (2014), Braun et al. (2015), Kessel et al. (2017), Stewart et al. (2018), Peel et al. (2019), Armstrong, Armstrong, Bennett, et al. (2020), Armstrong, Armstrong, McGregor, et al. (2020)
Short‐fin mako shark (*Isurus oxyrinchus*)	Feeding, reproduction? temperature?	Globally, movement patterns are associated with patterns of resource distribution and oceanographic conditions. In Eastern Pacific, movement corresponds to seasonal increase in productivity, upwelling, and sardine abundance. Higher numbers of juvenile mako sharks are present in some areas, suggesting a female migration to pupping grounds. Globally, North–South movements depending on season are evident, but high variability might indicate differences in food availability rather than temperature drives movement.	Satellite telemetry (horizontal and vertical movements), remote sensing for temperature and productivity levels, prey life history, and spatial overlap inferred from other studies, potential nursery areas identified, modeling of habitat characteristics, stable isotope and stomach content analysis	MacNeill et al. (2005), Rogers et al. (2015), Vaudo et al. (2017), Byrne et al. (2019), Francis et al. (2019), Nasby‐Lucas et al. (2019)
Tiger shark (*Galeocerdo cuvier*)	Feeding, reproduction, temperature	Globally, movements are associated with prey aggregations, such as albatross and sea turtles and areas of high productivity. Female movement to gestation sites and nursery areas proposed. Genetic studies suggest natal philopatry in females. Males may migrate to increase mate encounter rates. Temperature appears to have an effect on movement in some areas, especially for animals at range limits.	Satellite telemetry (horizontal and vertical movements), acoustic telemetry, stable isotope analysis, analysis of spatio‐temporal overlap with prey species, prey life history, and movement tracks inferred from other studies, simultaneous tracking of sharks and prey, temperature measurements, oceanographic conditions measured, analysis of activity patterns and temperature‐correlation, catch rates, productivity level measurements, hormone level analysis for reproduction, ultrasounds, general preliminary hormone analysis, stomach‐content analysis, “critter‐cams”, genetics, mating scars observed, potential gestation areas identified, potential nursery areas identified.	Lowe et al. (1996), Heithaus (2001), Heithaus et al. (2007), Whitney and Crow (2007), Meyer et al. (2010), Fitzpatrick et al. (2012), Hammerschlag, Gallagher, et al. (2012), Heithaus et al. (2012), Papastamatiou et al. (2013), Holmes et al. (2014), Werry et al. (2014), Ferreira et al. (2015), Hammerschlag et al. (2015), Lea, Wetherbee, et al. (2015), Bernard et al. (2016), Hammerschlag et al. (2016), Sulikowski et al. (2016), Acuña‐Marrero et al. (2017), Dicken et al. (2017), Ferreira et al. (2017), Daly et al. (2018), Lea et al. (2018), Meyer et al. (2018), Payne et al. (2018), Pirog et al. (2019), Ajemian et al. (2020), Lipscombe et al. (2020)
White shark (*Carcharodon carcharias*)	Reproduction, feeding, temperature?	Sharks aggregate in an oceanic area near Hawaii, presumably to mate. No concentrated food source around aggregation site, sharks return to coastal waters seemingly emaciated. Additionally, stable isotope analysis reveals reduced feeding at aggregation site (at least in males, female data lacking). This suggests mating main reason for off‐shore migrations in Eastern Pacific. Globally, Movements have been associated with prey aggregations and general productivity. Seasonal upwelling could directly affect white sharks. Show seasonal association with eddies in Atlantic and Pacific. Movement of females to coastal pupping sites.	Satellite telemetry (horizontal and vertical movements), acoustic telemetry, temperature measurements, prey life history and spatial overlap with prey inferred from other studies, visual behavioral observations, potential nursery areas identified, oceanographic and environmental data, stable isotope and stomach content analysis, genetics	Bonfil et al. (2005), Bruce et al. (2006), Weng et al. (2007), Domeier and Nasby‐Lucas (2008), Nasby‐Lucas et al. (2009), Jorgensen et al. (2010), Carlisle et al. (2012), Hussey et al. (2012), Jorgensen et al. (2012), Domeier and Nasby‐Lucas (2013), Malpica‐Cruz et al. (2013), Curtis et al. (2014), Skomal et al. (2017), Gaube et al. (2018), Huveneers et al. (2018), Bradford et al. (2020), Spaet et al. (2020)

## 5 STEP 2: EVIDENCE OF CONTEXT‐DEPENDENCE IN THE STUDY SPECIES

Despite the commonality of basic life history requirements related to overall movement behavior for most species found in step 1, in step 2 it is evident that many elasmobranchs exhibit high intra‐specific variability of movement behavior among and within populations and that varying movement patterns exist across multiple scales (Table S2), suggesting context‐dependence. Many species vary in movement behavior across and within regions, and individuals can change their movement patterns over time (Table S2). Different movement strategies include differing directions and destinations, the extent of movement (large‐scale vs. small‐ scale, resident vs. migratory), as well as timing. Sex and size‐classes can differ in movement strategies (Tables S1 and S2). This re‐emphasizes the point made by Nathan et al. (2008) that a key challenge in animal movement ecology remains the identification of specific external and internal factors shaping the movement of individuals. While life history requirements, such as feeding and reproduction, are central to every individual within a mobile species, high variability in movement patterns supports the notion that the shape movement patterns take on to fulfill life history requirements is dependent on contextual factors that change across time, space, and individuals and shape behavioral trait expression.

## 6 STEP 3: PUTTING MOVEMENT INTO CONTEXT: SYNTHESIZING STEPS 1 AND 2 WITH REMARKS TO AVIAN LITERATURE

The third step of a context‐focused approach is based on a systematic comparison of the differences in movement patterns within the study systems (gathered in step one and two) to the differences in contextual factors (Figure 2). This step specifically investigates how the behavioral traits exhibited to fulfill life history requirements are shaped by contextual factors and how predictions can be made about which contextual factors drive certain movement patterns.

The theoretical basis for how biotic and abiotic factors may drive movement and the range of possible mechanisms responsible for intra‐specific variability is much more developed in the avian literature. Additionally, as steps 1 and 2 of our context‐focused approach are also well established in birds, when interpreted under a context‐dependence framework, bird movement studies may aid in filling knowledge gaps in less‐studied taxa. Therefore, we draw on this literature to provide a useful roadmap for applying a context‐focused approach and to provide examples of mechanisms potentially influencing intra‐specific variability not yet explored in other taxa, such as elasmobranchs (Side‐Box 2, 2, 3).

### 6.1 Environmental context

The influence of environmental context on intra‐specific variability of movement has been extensively studied in birds (see Side‐Box 1). In some species, populations show strong differences in movement patterns based on latitudinal distribution and habitat types (Side‐Box 1). Differing resource distribution, winter conditions at breeding sites and time‐resource trade‐offs between populations are well‐established drivers of individual movement decisions in this taxon (Side‐Box 1).

In elasmobranchs, many individuals of a species inhabit waters of varying climatic conditions and as such, experience different current regimes and habitats. For example, geographically varying oceanographic conditions can result in distinct patterns of resource distribution and magnitude of seasonal environmental change. While reef manta rays (*Manta alfredi*) undergo large‐scale movements in the dynamic, highly seasonal East Australian Current, small‐scale movements are evident in the Red Sea where oceanographic conditions result in year‐round productivity and less environmental fluctuations, such as in temperature (Braun et al., 2015; Jaine et al., 2014). This comparison between the two populations suggests that oceanographic conditions influence behavioral trait expression linked to foraging and behavioral thermoregulation, which results in differences in movement patterns (Armstrong, Armstrong, Bennett, et al., 2020; Braun et al., 2015; Jaine et al., 2014).

Overall, animals at the range‐limit of their distribution are often subject to larger environmental fluctuations, especially in temperature (Heupel et al., 2015; Holmes et al., 2014). The optimal temperature niche at which Tiger sharks (*Galeocerdo cuvier*) exhibit highest activity levels and abundance has been found to be 22–24°C (Payne et al., 2018). Along Australia's East Coast this species demonstrates differences in movement strategies based on latitudinal distribution (Holmes et al., 2014; Lipscombe et al., 2020). Animals tagged at the range limit, in New South Wales, where temperatures drop below the optimal temperature niche in the Austral winter, move north to warmer latitudes (Holmes et al., 2014; Lipscombe et al., 2020). In the center of distribution, such as Central and North Queensland, where seasonal temperatures rarely drop below this temperature niche (Payne et al., 2018), individuals show high variability in movement patterns, with localized movements, longitudinal offshore movements, and resident behavior (Fitzpatrick et al., 2012; Holmes et al., 2014; Lipscombe et al., 2020). Thus, tiger sharks along the east coast of Australia are subject to differing environmental contexts. Animals in subtropical and tropical areas are not bound to behavioral traits aimed at avoiding low winter temperatures, compared to individuals at the range limit, thus allowing for diversification of movement strategies. However, in Northern Queensland, temperatures can often exceed the optimal niche found by Payne et al. (2018). For example, around Raine Island, the world's largest green turtle (*Chelonia mydas*) nesting site, some tiger sharks are exposed to temperatures over 30°C for prolonged periods (Fitzpatrick et al., 2012). Here, abundant and easily available prey in the form of dead and weakened green turtles (Hammerschlag et al., 2016) may create a trade‐off that allows tiger sharks to remain in sub‐optimal temperatures.

SIDE‐BOX 2 **Environmental context: Comparison to a well‐studied system—birds**

Globally, intra‐specific variability in bird movement patterns is driven by differences in environmental context, principally temperature conditions, resource availability, and geographical distance between breeding and nonbreeding sites (Ketterson et al., 2015). Variability provides detailed insights into bird movement drivers. It is well established that bird movement patterns, such as routes and timing, vary based on local environmental conditions (e.g., Vardanis et al., 2011). However, movement patterns are also shaped by expected conditions at the destination (Kuang et al., 2020). A strong relationship between latitude and movement patterns exists in the East of North America as well as Western Europe. Individuals at higher latitudes, with stronger seasonal changes in temperature and food availability show higher migration propensity (Ketterson et al., 2015; Newton & Dale, 1996). Pied avocets, for example, are mostly resident in France, while undergoing longer movements at higher latitudes. This is due to more severe winters resulting in a stronger decrease in habitat suitability (Chambon et al., 2018).

Leapfrog migration is a common source of movement variability between bird populations. Here, populations breeding further north, overwinter further south than more southerly breeding populations. Therefore, these more northerly breeding populations migrate further and skip over southerly breeding populations. Such patterns are attributed to latitudinal differences in spring arrival, thus creating a time‐resource trade‐off for different populations (Bell, 1996).

Even within smaller geographical regions, individuals of the same population may encounter different environmental conditions causing them to migrate differently. Nonmigratory vs. migratory greater sage‐grouses, for example, experience different degrees of seasonal change in terms of plant desiccation, snow accumulation and precipitation causing partial altitudinal migration in this species (Pratt et al., 2017).

### 6.2 Ecological context

Studies on bird migration show that ecological factors drive intra‐specific variability in movement (Side‐Box 2). Competition among conspecifics, varying predation risks among regions and seasonally varying prey availability are thought to drive bird movement (side‐Box 2). For elasmobranchs seasonally changing prey availability and prey species composition have been shown to drive movement patterns, while the role of predation risk and competition are less well understood (Tables 1 and 2).

**TABLE 2:** Examples of variation in shark and ray movement patterns (For a more extended version of Table 2, covering 18 species, see Appendix S1)

Species	Differences in movement behavior within regions?	Differences in movement behavior between regions?	Does the species aggregate naturally (no artificial feeding)?	Evidence of context‐dependence	Source
Broadnose sevengill shark (*Notorynchus cepedianus*)	Yes	Yes	Yes	Partial migration is evident in Tasmania and reproductive strategies of sexes (individual context) might shape movement decisions within the population. Sequential tracking of sharks and prey suggests migration due to prey concentrations with individual site fidelity evident. Reproductive studies rule out breeding as reason for movement into coastal Tasmania. However, movement to suspected nursery areas in USA may take place. Differential movement and resource separation by individuals occurs in Tasmania. Females make larger movements in the United States, while males do in Australia.	Barnett, Abrantes, Stevens, Yick, et al. (2010); Barnett, Abrantes, Stevens, Bruce, and Semmens (2010); Barnett, Redd, Frusher, Stevens, and Semmens (2010); Barnett, Stevens, Frusher, and Semmens (2010), Abrantes and Barnett (2011), Barnett et al. (2011), Williams et al. (2011), Williams et al. (2012), Stehfest et al. (2014)
Bull shark (*Carcharhinus leucas*),	Yes	Yes	Yes	Movement in Australia and Southern Africa potentially driven by seasonal changes in temperature and food availability. Movements in South‐Eastern United States more limited, potentially due to more stable conditions. Bull sharks exhibit high variability in movement patterns within regions, with some individuals moving large distances while others remain more localized. Animals at range limits appear to migrate differentially to animals in center. Skipped‐breeding migration and natal philopatry to river systems could result in variability among females.	Tillett et al. (2012), Daly et al. (2013), Daly et al. (2014), Heupel et al. (2015), Lea, Humphries, et al. (2015), Espinoza et al. (2016), Lee et al. (2019)
Reef manta *(Mobula alfredi)*	Yes	Yes	Yes	Rays encountering dynamic oceanographic conditions in Australia resulting in seasonal upwelling show large‐scale movements, while rays in the Red Sea show limited movements suggesting stable oceanographic conditions result in relatively favorable conditions year‐round. In some regions, females may migrate to separate birthing grounds while in other areas adults and neonates overlap spatially	Marshall and Bennett (2010), Jaine et al. (2014), Braun et al. (2015), Kessel et al. (2017)
Short‐fin mako shark (*Isurus oxyrinchus*)	Yes	Yes	NA	Different populations show resident vs. transient behaviors based on seasonal consistency of primary production. Differences in magnitude of environmental change shapes different movement patterns in different regions.	Vaudo et al. (2017), Byrne et al. (2019), Francis et al. (2019)
Tiger shark (*Galeocerdo cuvier*)	Yes	Yes	Yes	Movements are more restricted around oceanic islands with narrow shelves compared to movements in areas with more extensive shelves, such as along continents. Movement patterns appear more restricted in Southern Africa and Eastern Australia compared to Atlantic and Western Australia. Partial migrations evident in most populations. Movements in Hawaii differ between regions, depending on resource distribution around islands. Movement also differs based on sex, suggesting reproductive strategies and breeding cycles shape movement. Size classes differ in movement behavior and in their response to seasonal environmental factors. Stable isotope values differ between regions in Australia and so do extent of movements, suggesting a link between feeding behavior and movement strategy. Animals at range limits appear to migrate differently to animals in center of distribution. Despite being thermal generalist, in some regions tiger sharks respond differently to temperature. Latitudinal vs. longitudinal movements in different regions of Australia.	Heithaus et al. (2007), Fitzpatrick et al. (2012), Hammerschlag, Luo, et al. (2012), Papastamatiou et al. (2013), Holmes et al. (2014), Werry et al. (2014), Ferreira et al. (2015), Lea, Wetherbee, et al. (2015), Acuña‐Marrero et al. (2017), Ferreira et al. (2017), Daly et al. (2018), Lea et al. (2018), Meyer et al. (2018), Ajemian et al. (2020), Lipscombe et al. (2020)
White shark (*Carcharodon carcharias*)	Yes	Yes	Yes	Offshore movements occur in some populations but patterns are less clear in Atlantic while more synchronized in the Eastern Pacific. Sex‐and size‐specific migrations in Eastern Pacific and Southern Australia. Latitudinal vs. longitudinal movements in different regions. Females may differ in movement patterns based on reproductive cycle. Seasonal North–South movements in Atlantic staggered between individuals, despite changing temperatures, suggesting individual context may play a role. Environmental preferences appear to differ between Atlantic and Pacific.	Domeier and Nasby‐Lucas (2013), Skomal et al. (2017), Gaube et al. (2018), Bradford et al. (2020)

A suite of studies on broadnose sevengill sharks (*Notorynchus cepedianus)* in southern Tasmania, Australia provided insight into their drivers of migration into coastal systems (Abrantes & Barnett, 2011; Awruch et al., 2014; Barnett, Abrantes, Stevens, Bruce, & Semmens, 2010; Barnett, Abrantes, Stevens, Yick, et al., 2010; Barnett, Redd, Frusher, Stevens, & Semmens, 2010; Barnett & Semmens, 2012; Barnett, Stevens, Frusher, & Semmens, 2010). Although most sevengill sharks leave coastal areas in winter, some females remain, suggesting that temperature per se is not the only key driver of their movement (Barnett et al., 2011; Barnett, Stevens, Frusher, & Semmens, 2010). The absence of neonates and smaller juveniles (<100 cm) (Barnett, Stevens, Frusher, & Semmens, 2010), low number of females with mating scars and their reproductive status (e.g., no near‐term pregnant individuals) ruled out reproduction as a driver for movement into the area (Awruch et al., 2014). Diet analysis and estimates of predator—prey abundance combined with similar movement patterns, seasonality, and high spatial overlap with prey provide strong, contextually explicit support that sevengill sharks move into coastal systems in Tasmania following seasonally abundant prey resources (Barnett & Semmens, 2012). Here, a comparison across contexts, involving the tracking of prey animals, ruled out factors unlikely to be responsible for movement while narrowing down the most likely driver—high seasonal prey availability.

Individuals within a population can also be distributed across different ecological contexts (Meyer et al., 2018). Tiger sharks from the Hawaiian archipelago are reproductively connected and show spatial overlap around Maui during the proposed breeding season (Meyer et al., 2018). For the rest of the year, some individuals spread out across the Hawaiian Islands, while some remain localized near Maui (Meyer et al., 2018). The context of this island likely provides optimal feeding opportunities as well as suitable environmental conditions, as residents fulfill all requirements there. Meyer et al. (2018) state that this region has some of the highest levels of primary production of any Pacific Island area and likely offers year‐round prey. In contrast, conditions at other Hawaiian Islands may drive seasonal movements of nonresident individuals, for example, in other parts of the archipelago tiger sharks make large‐scale movements tracking prey aggregations that are dispersed and only seasonally available, such as fledging albatross (Meyer et al., 2010). Due to differences in the spatio‐temporal distribution of prey resources across the archipelago varying foraging strategies result in differential movements within this population of tiger sharks (Humphries et al., 2010; Meyer et al., 2010, 2018).

SIDE‐BOX 2 **Ecological context: Comparison to a well‐studied system—birds**

Movement theory on birds provides well‐established examples of how ecological context creates intra‐specific variability in movement patterns and how variability can offer great insights into overall movement drivers. Chain migration, where northerly breeding populations overwinter at breeding sites of more southerly populations which in turn move even further south to overwinter have been observed in swifts (Åkesson et al., 2020). Competition may force some individuals to overwinter further north, while others remain south to breed. These southern breeding birds are also able to time their movements south earlier than northern conspecifics in order to take advantage of seasonally predictable insect abundance at lower latitudes (Åkesson et al., 2020).

Altitudinal migration in neo‐tropical birds is driven by density‐dependent predation risk (Boyle, 2008a). As predation risk can vary between location movement may also differ. Indeed, Lank et al. (2003) showed that different sandpiper populations vary in timing and distance of seasonal movement. This variability appears to be a response to varying levels of predation risk in each region (Lank et al., 2003). Furthermore, barnacle geese migrating from the Netherlands to Russia changed their migratory behavior in response to increasing predator populations at Baltic stop‐over sites (Jonker et al., 2010). In contrast, barnacle geese migrating from the United Kingdom to Norway did not show a change in migratory behavior suggesting that local predation risk in the Baltic rather than long‐term environmental change caused the shift in movement in the former population (Tombre et al., 2008).

### 6.3 Individual context

There is extensive literature showing that individual context drives intra‐specific variability in bird movement patterns. Dominance, physical condition, body size, breeding strategies, and individual feeding specialization have been established as drivers of movement in birds (Side‐Box 3). Thus, the avian literature allows for a context‐focused approach to study movement drivers (see Side‐Box 3).

The influence of individual context has been less studied in elasmobranchs. Nevertheless, some information suggests it may play a role in shaping movement patterns. For instance, some species have distinct nursery grounds that females migrate to, to give birth. For example, bull sharks (*Carcharhinus leucas*) are believed to exhibit natal philopatry where females return to the same river they were born in to pup each reproductive cycle (Lea, Humphries, et al., 2015; Tillett et al., 2012). Being a species that can roam over large distances outside of the pupping season (over 1700 km along the East Coast of Australia, Espinoza et al., 2016, Heupel et al., 2015), natal philopatry may drive overall variability in how females fulfill their life history requirements because of the distance between individual pupping and feeding sites.

White sharks in the Eastern Pacific show different periodicity and spatial scales of movements between males and females. Such sex differences suggest variability in behavioral traits linked to reproduction (Domeier & Nasby‐Lucas, 2013). Both sexes overlap spatially for short periods, increasing mate encounter rates. While males visit aggregation sites every year, females only do so every two years, likely based on their reproductive cycle (Domeier & Nasby‐Lucas, 2013). After aggregating, females move over large distances and experience higher water temperatures than males, potentially to expedite gestation (Domeier & Nasby‐Lucas, 2013). Some females then return to coastal sites for parturition (Domeier & Nasby‐Lucas, 2013). This coincides with young‐of‐the‐year white sharks observed along the coast (Domeier & Nasby‐Lucas, 2013). In this example, sex differences in behavioral traits linked to reproductive success result in differential movements. Females possibly require three distinct locations, as they need to mate, gestate, and give birth. Hence, female sharks move further and show bi‐annual periodicity in movement. Males, on the other hand aim to increase reproductive output by mating every year and thus move over smaller spatial scales with annual return movements (Domeier & Nasby‐Lucas, 2013).

SIDE‐BOX 3 **Internal context: Comparison to a well‐studied system—birds**

Intra‐specific variability in bird movement patterns is often related to internal differences of individuals. Such variation has been attributed to energetic condition, for example. Differences in fat stores and molting stage produce varying movement patterns (Goymann et al., 2010; Stutchbury et al., 2011).

Benefits of arriving early at breeding areas for males and dominance patterns have been established as drivers of movement in birds creating intra‐specific variability (Chapman et al., 2011; Fudickar et al., 2013).

Body size also plays an important role in bird movement and intra‐specific variability. In general larger individuals often migrate less than smaller conspecifics in winter, as they have higher thermal tolerance or are able to fast longer during unfavorable conditions (Boyle, 2008b; Ketterson & Nolan Jr, 1976). Conversely, in hotter climates, movement of larger individuals of a population can be driven by limited tolerance to warmer temperatures (Alonso et al., 2009).

Some evidence from diet studies on partially migratory kestrels suggests that intra‐specific differences in feeding niche could explain why some individuals migrate as seasonal shifts in food availability might affect individuals differently (Aparicio, 2000).

Philopatry to nesting and mating sites has been demonstrated to shape movement patterns in birds (Alonso et al., 2000). Additionally, Skipped‐breeding migrations are a common source of intra‐specific variability in sea bird movement (Shaw & Levin, 2011). Individuals in less good condition may skip migration to breeding grounds.

Individual context also incorporates other aspects, such as body size. In elasmobranchs different size classes often show varying movement patterns (Lea et al., 2018) and size‐related diet changes may drive differential movements as size classes track different prey animals (Ajemian et al., 2020; Lea et al., 2018; Lea, Wetherbee, et al., 2015). Additionally, differences in movement patterns may relate to differences in energetics and locomotive abilities (Fu et al., 2016; Lawson et al., 2019). According to allometric scaling of metabolic rates in elasmobranchs, larger body size may reduce cost of transport and allow larger individuals to roam further (Lawson et al., 2019) utilizing a wider range of habitats for foraging.

## 7 IMPLICATIONS OF A CONTEXT‐FOCUSED APPROACH AND FUTURE RESEARCH DIRECTIONS

The strength in a comparative, context‐focused approach lays in its macroecological perspective (e.g., Somveille et al., 2015). For example, seasonal migration in many species is believed to be driven by thermal constraints based on low winter temperatures (Shaw, 2016). However, a correlation between animal movement and temperature can often mask the actual set of contextual factors responsible for a given movement such as prey species occurrence, competition, and low resource availability (Barnett, Stevens, Frusher, & Semmens, 2010; Somveille et al., 2015). Thus, a context‐approach comparing movement patterns and temperature exposure for individuals across different latitudes and/or investigating multiple contextual factors can aid in reducing masking effects (e.g., Barnett, Stevens, Frusher, & Semmens, 2010; Daly et al., 2014; Espinoza et al., 2016; Lee et al., 2019).

Additionally, taxa‐wide, tagging biases have long been a problem in tracking studies (Hays et al., 2020). Estimates of spatio‐temporal patterns of animal movement are often weighted toward the tagging location, with areas further away from the tagging site being underrepresented (Hays et al., 2020). Context could be utilized to overcome tagging‐location biases. If the contextual factors driving certain movement patterns of one study system have been identified, increased confidence in predictions and models about movements in other systems, similar in context, could help close gaps in knowledge of movement patterns in new or understudied systems/locations. Furthermore, locations where animals are tagged are often chosen based on accessibility and high abundance of study species. If the context under which study species aggregate is established (such as in Copping et al., 2018) further tagging locations could be explored based on similar contextual factors which could then produce more representative sample sizes from the greater population. A context‐focused approach can also aid in predictions about movement as a behavioral response, if the contextual factors driving movement are known, models and predictions about future environmental change can be more precise. For example, climate change, habitat degradation, and overfishing influence populations of many shark species (Dulvy et al., 2014; Vedor et al., 2021) and a context‐focused approach can be utilized to predict how future anthropogenic change will affect movement patterns. Shifts and changes in migration timing and routes potentially due to changing climates have already been observed in birds (Jenni & Kéry, 2003) and similar shifts are expected in sharks and rays (Birkmanis et al., 2020; Niella et al., 2020). However, without systematically understanding the contextual factors driving movement for populations and individuals, the scale and direction of shifts in movement behavior due to global change cannot be easily predicted. Much of the variation in movement patterns likely constitutes individual responses to specific contextual factors that differ across time, space, and individuals. Thus, blanket management/conservation measures might not be applicable to a species across its range and future measures can benefit from being informed by a context‐approach that disentangles movement drivers of different populations and demographic groups and takes into account variability among regions (Table 3).

**TABLE 3:** Avenues of future research into movement drivers

Systematic comparisons of movement patterns and environmental factors between different regions (see e.g., Vaudo et al., 2017) Increased incorporation of remotely sensed environmental data into elasmobranch movement studies (see e.g., Lee et al., 2019) Context‐focused investigations of global aggregation sites (see e.g., Copping et al., 2018) Incorporation of trophic and dietary information into movement studies to track individual foraging patterns across diverse and distant environments Incorporation of health and energetics through analysis of body condition, blood hormones and lipid reserves, as well as energetics models into movement studies (see e.g., AtallahBenson et al., 2020; Gallagher et al., 2014; Moorhead et al., 2021) Identification of important prey species and simultaneous tracking of potential prey and predator (see e.g., Barnett & Semmens, 2012, Hammerschlag, Luo, et al., 2012) Combination of hormone studies and ultra‐sounds to study female reproductive movements (see e.g., Awruch et al., 2014; Barnett et al., 2019; Sulikowski et al., 2016) Genetics studies in combination with movement studies to elucidate the relationship between individually varying movement patterns and natal philopatry Systematic investigation of global nursery areas to reveal potential relationship between regional differences in juvenile survival benefits and female movement strategies for parturition

## 8 CONCLUSION

A stepwise, context‐focused approach to studying animal movement can aid in elucidating internal and external factors shaping individual movement patterns. While life history requirements such as breeding, feeding, and remaining in suitable environmental conditions drive overall behavior and are central to each individual, many taxa, including elasmobranchs show high variability and complexity in movement patterns across and within populations. The occurrence of multiple movement strategies within a species is driven by contextual factors that differ across time, space, and individuals. These factors shape how behavioral traits related to life history requirements are expressed across time and space and thus shape individual movement decisions. Overall, our systematic comparison between study systems with regard to contextual factors, provides a framework to incorporate variability as a tool to determine combinations of contextual factors that can be reliably related to a specific movement outcome. Capturing complexity and variability across populations and demographic groups can help further our understanding of animal movement. This approach is applicable to any taxa showing variability in movement patterns. Additionally, a context‐focused approach can aid in overcoming tagging biases, making predictions about movement responses to environmental change and designing efficient conservation measures.

## AUTHOR CONTRIBUTIONS

**Nicolas Lubitz:** Conceptualization (lead); investigation (lead); methodology (lead); writing – original draft (lead); writing – review and editing (lead). **Michael Bradley:** Conceptualization (equal); writing – review and editing (equal). **Marcus Sheaves:** Supervision (equal); writing – review and editing (equal). **Neil Hammerschlag:** Writing – review and editing (equal). **Ryan Daly:** Writing – review and editing (equal). **Adam Barnett:** Conceptualization (equal); supervision (equal); writing – review and editing (equal).

## CONFLICT OF INTEREST

The authors declare no conflict of interest.

## Supporting information


Appendix S1

## Data Availability

No Data was used for this manuscript.

## References

[ece39128-bib-0001] Abrantes, K. G. , & Barnett, A. (2011). Intrapopulation variations in diet and habitat use in a marine apex predator, the broadnose sevengill shark *Notorynchus cepedianus* . Marine Ecology Progress Series, 442, 133–148.

[ece39128-bib-0002] Acuña‐Marrero, D. , Smith, A. N. , Hammerschlag, N. , Hearn, A. , Anderson, M. J. , Calich, H. , Pawley, M. D. M. , Fischer, C. , & Salinas‐de‐León, P. (2017). Residency and movement patterns of an apex predatory shark (*Galeocerdo cuvier*) at the Galapagos Marine Reserve. PLoS One, 12(8), e0183669. 28829820 10.1371/journal.pone.0183669 PMC5567640

[ece39128-bib-0003] Ajemian, M. J. , Drymon, J. M. , Hammerschlag, N. , Wells, R. D. , Street, G. , Falterman, B. , McKinney, J. , Driggers, W. B., 3rd , Hoffmayer, E. R. , Fischer, C. , & Stunz, G. W. (2020). Movement patterns and habitat use of tiger sharks (*Galeocerdo cuvier*) across ontogeny in the Gulf of Mexico. PLoS One, 15(7), e0234868. 32667920 10.1371/journal.pone.0234868 PMC7363083

[ece39128-bib-0004] Åkesson, S. , Atkinson, P. W. , Bermejo, A. , de la Puente, J. , Ferri, M. , Hewson, C. M. , Holmgren, J. , Kaiser, E. , Kearsley, L. , Klaassen, R. H. G. , Kolunen, H. , Matsson, G. , Minelli, F. , Norevik, G. , Pietiäinen, H. , Singh, N. J. , Spina, F. , Viktora, L. , & Hedenström, A. (2020). Evolution of chain migration in an aerial insectivorous bird, the common swift *Apus apus* . Evolution, 74(10), 2377–2391. 32885859 10.1111/evo.14093 PMC7589357

[ece39128-bib-0005] Alonso, J. C. , Morales, M. B. , & Alonso, J. A. (2000). Partial migration, and lek and nesting area fidelity in female great bustards. The Condor, 102(1), 127–136.

[ece39128-bib-0006] Alonso, J. C. , Palacín, C. , Alonso, J. A. , & Martín, C. A. (2009). Post‐breeding migration in male great bustards: Low tolerance of the heaviest Palaearctic bird to summer heat. Behavioral Ecology and Sociobiology, 63(12), 1705–1715.

[ece39128-bib-0007] Anderson, R. C. , Adam, M. S. , & Goes, J. I. (2011). From monsoons to mantas: Seasonal distribution of Manta alfredi in the Maldives. Fisheries Oceanography, 20(2), 104–113.

[ece39128-bib-0008] Andersson, M. (1982). Sexual selection, natural selection and quality advertisement. Biological Journal of the Linnean Society, 17, 375–393.

[ece39128-bib-0009] Aparicio, J. M. (2000). Differences in the diets of resident and non‐resident Kestrels in Spain. Ornis Fennica, 77(4), 169–175.

[ece39128-bib-0010] Armstrong, A. J. , Armstrong, A. O. , Bennett, M. B. , McGregor, F. , Abrantes, K. G. , Barnett, A. , Richardson, A. J. , Townsend, K. A. , & Dudgeon, C. L. (2020). The geographic distribution of reef and oceanic manta rays (*Mobula alfredi* and *Mobula birostris*) in Australian coastal waters. Journal of Fish Biology, 96(3), 835–840. 31925780 10.1111/jfb.14256

[ece39128-bib-0011] Armstrong, A. J. , Armstrong, A. O. , McGregor, F. , Richardson, A. J. , Bennett, M. B. , Townsend, K. A. , Hays, G. C. , van Keulen, M. , Smith, J. , & Dudgeon, C. L. (2020). Satellite tagging and photographic identification reveal connectivity between two UNESCO World Heritage Areas for reef manta rays. Frontiers in Marine Science, 7, 725.

[ece39128-bib-0012] AtallahBenson, L. , Merly, L. , Cray, C. , & Hammerschlag, N. (2020). Serum protein analysis of nurse sharks. Journal of Aquatic Animal Health, 32(2), 77–82. 32012365 10.1002/aah.10100

[ece39128-bib-0013] Awruch, C. A. , Jones, S. M. , Asorey, M. G. , & Barnett, A. (2014). Non‐lethal assessment of the reproductive status of broadnose sevengill sharks (*Notorynchus cepedianus*) to determine the significance of habitat use in coastal areas. Conservation Physiology, 2(1), cou013. 27293634 10.1093/conphys/cou013 PMC4806732

[ece39128-bib-0014] Barnett, A. , Abrantes, K. , Stevens, J. D. , Yick, J. L. , Frusher, S. D. , & Semmens, J. M. (2010). Predator‐prey relationships and foraging ecology of a marine apex predator with a wide temperate distribution. Marine Ecology Progress Series, 416, 189–200. 10.3354/meps08778

[ece39128-bib-0015] Barnett, A. , Abrantes, K. G. , Stevens, J. D. , Bruce, B. D. , & Semmens, J. M. (2010). Fine‐scale movements of the broadnose sevengill shark and its main prey, the gummy shark. PLoS One, 5(12), e15464. 10.1371/journal.pone.0015464 21151925 PMC2997065

[ece39128-bib-0016] Barnett, A. , Abrantes, K. G. , Stevens, J. D. , & Semmens, J. M. (2011). Site fidelity and sex‐specific migration in a mobile apex predator: Implications for conservation and ecosystem dynamics. Animal Behaviour, 81(5), 1039–1048. 10.1016/j.anbehav.2011.02.011

[ece39128-bib-0017] Barnett, A. , McAllister, J. D. , Semmens, J. , Abrantes, K. , Sheaves, M. , & Awruch, C. (2019). Identification of essential habitats: Including chimaeras into current shark protected areas. Aquatic Conservation: Marine and Freshwater Ecosystems, 29(6), 865–880. 10.1002/aqc.3087

[ece39128-bib-0018] Barnett, A. , Redd, K. S. , Frusher, S. D. , Stevens, J. D. , & Semmens, J. M. (2010). Non‐lethal method to obtain stomach samples from a large marine predator and the use of DNA analysis to improve dietary information. Journal of Experimental Marine Biology and Ecology, 393(1–2), 188–192, 192. 10.1016/j.jembe.2010.07.022

[ece39128-bib-0019] Barnett, A. , & Semmens, J. M. (2012). Sequential movement into coastal habitats and high spatial overlap of predator and prey suggest high predation pressure in protected areas. Oikos, 121(6), 882–890. 10.1111/j.1600-0706.2011.20000.x

[ece39128-bib-0020] Barnett, A. , Stevens, J. D. , Frusher, S. D. , & Semmens, J. M. (2010). Seasonal occurrence and population structure of the broadnose sevengill shark *Notorynchus cepedianus* in coastal habitats of south‐east Tasmania. Journal of Fish Biology, 77(7), 1688–1701. 10.1111/j.1095-8649.2010.02810.x 21078027

[ece39128-bib-0021] Bauer, S. , & Hoye, B. J. (2014). Migratory animals couple biodiversity and ecosystem functioning worldwide. Science, 344(6179), 54. 10.1126/science.1242552 24700862

[ece39128-bib-0022] Bell, C. P. (1996). Seasonality and time allocation as causes of leap‐frog migration in the Yellow Wagtail Motacilla flava. Journal of Avian Biology, 27, 334–342.

[ece39128-bib-0023] Bels, V. , & Whishaw, I. Q. (2019). Feeding in vertebrates: Evolution, morphology, behavior, biomechanics. Springer.

[ece39128-bib-0024] Bergmann, C. (1848). Über die verhältnisse der wärmeökonomie der thiere zu ihrer grösse. Vandenhoeck & Ruprecht.

[ece39128-bib-0025] Bernard, A. M. , Feldheim, K. A. , Heithaus, M. R. , Wintner, S. P. , Wetherbee, B. M. , & Shivji, M. S. (2016). Global population genetic dynamics of a highly migratory, apex predator shark. Molecular Ecology, 25(21), 5312–5329. 10.1111/mec.13845 27662523

[ece39128-bib-0026] Birkmanis, C. A. , Freer, J. J. , Simmons, L. W. , Partridge, J. C. , & Sequeira, A. M. (2020). Future distribution of suitable habitat for pelagic sharks in Australia under climate change models. Frontiers in Marine Science, 7, 570.

[ece39128-bib-0027] Block, B. A. , Jonsen, I. D. , Jorgensen, S. J. , Winship, A. J. , Shaffer, S. A. , Bograd, S. J. , Hazen, E. L. , Foley, D. G. , Breed, G. A. , Harrison, A. L. , Ganong, J. E. , Swithenbank, A. , Castleton, M. , Dewar, H. , Mate, B. R. , Shillinger, G. L. , Schaefer, K. M. , Benson, S. R. , Weise, M. J. , … Costa, D. P. (2011). Tracking apex marine predator movements in a dynamic ocean. Nature, 475(7354), 86–90. 10.1038/nature10082 21697831

[ece39128-bib-0028] Bonfil, R. , Meÿer, M. , Scholl, M. C. , Johnson, R. , O'Brien, S. , Oosthuizen, H. , Swanson, S. , Kotze, D. , & Paterson, M. (2005). Transoceanic migration, spatial dynamics, and population linkages of white sharks. Science, 310(5745), 100–103. 10.1126/science.1114898 16210537

[ece39128-bib-0029] Boyle, W. A. (2008a). Can variation in risk of nest predation explain altitudinal migration in tropical birds? Oecologia, 155(2), 397–403. 18188606 10.1007/s00442-007-0897-6

[ece39128-bib-0030] Boyle, W. A. (2008b). Partial migration in birds: Tests of three hypotheses in a tropical lekking frugivore. Journal of Animal Ecology, 77(6), 1122–1128. 18657208 10.1111/j.1365-2656.2008.01451.x

[ece39128-bib-0031] Bradford, R. , Patterson, T. A. , Rogers, P. J. , McAuley, R. , Mountford, S. , Huveneers, C. , Robbins, R. , Fox, A. , & Bruce, B. D. (2020). Evidence of diverse movement strategies and habitat use by white sharks, *Carcharodon carcharias*, off southern Australia. Marine Biology, 167(7), 1–12. 10.1007/s00227-020-03712-y

[ece39128-bib-0032] Bradley, M. , Baker, R. , Nagelkerken, I. , & Sheaves, M. (2019). Context is more important than habitat type in determining use by juvenile fish. Landscape Ecology, 34(2), 427–442. 10.1007/s10980-019-00781-3

[ece39128-bib-0033] Bradley, M. , Nagelkerken, I. , Baker, R. , & Sheaves, M. (2020). Context dependence: A conceptual approach for understanding the habitat relationships of coastal marine fauna. Bioscience, 70(11), 986–1004. 10.1093/biosci/biaa100

[ece39128-bib-0034] Braun, C. D. , Skomal, G. B. , Thorrold, S. R. , & Berumen, M. L. (2015). Movements of the reef manta ray (*Manta alfredi*) in the Red Sea using satellite and acoustic telemetry. Marine Biology, 162(12), 2351–2362.

[ece39128-bib-0035] Bruce, B. , Stevens, J. , & Malcolm, H. (2006). Movements and swimming behaviour of white sharks (*Carcharodon carcharias*) in Australian waters. Marine Biology, 150(2), 161–172.

[ece39128-bib-0036] Brunnschweiler, J. M. , & Barnett, A. (2013). Opportunistic visitors: Long‐term behavioural response of bull sharks to food provisioning in Fiji. PLoS One, 8(3), e58522. 10.1371/journal.pone.0058522 23516496 PMC3596312

[ece39128-bib-0037] Byrne, M. E. , Vaudo, J. J. , Harvey, G. C. M. , Johnston, M. W. , Wetherbee, B. M. , & Shivji, M. (2019). Behavioral response of a mobile marine predator to environmental variables differs across ecoregions. Ecography, 42(9), 1569–1578.

[ece39128-bib-0038] Carlisle, A. B. , Kim, S. L. , Semmens, B. X. , Madigan, D. J. , Jorgensen, S. J. , Perle, C. R. , Anderson S.D. , Chapple T.K. , Kanive P.E. , Block B.A. (2012). Using stable isotope analysis to understand the migration and trophic ecology of northeastern Pacific white sharks (*Carcharodon carcharias*). PLoS One, 7(2), e30492. 10.1371/journal.pone.0030492 22355313 PMC3280240

[ece39128-bib-0039] Carlson, J. K. , Ribera, M. M. , Conrath, C. L. , Heupel, M. R. , & Burgess, G. H. (2010). Habitat use and movement patterns of bull sharks *Carcharhinus leucas* determined using pop‐up satellite archival tags. Journal of Fish Biology, 77(3), 661–675. 10.1111/j.1095-8649.2010.02707.x 20701646

[ece39128-bib-0040] Chambert, T. , Rotella, J. J. , & Garrott, R. A. (2015). Female weddell seals show flexible strategies of colony attendance related to varying environmental conditions. Ecology, 96(2), 479–488. 10.1890/14-0911.1 26240869

[ece39128-bib-0041] Chambon, R. , Dugravot, S. , Paillisson, J. M. , Lemesle, J. C. , Ysnel, F. , & Gélinaud, G. (2018). Partial migration in inexperienced pied avocets Recurvirostra avosetta: Distribution pattern and correlates. Journal of Avian Biology, 49(6), e01549.

[ece39128-bib-0042] Chapman, B. B. , Bronmark, C. , Nilsson, J. A. , & Hansson, L. A. (2011). The ecology and evolution of partial migration. Oikos, 120(12), 1764–1775. 10.1111/j.1600-0706.2011.20131.x

[ece39128-bib-0043] Chapman, B. B. , Hulthen, K. , Brodersen, J. , Nilsson, P. A. , Skov, C. , Hansson, L. A. , & Bronmark, C. (2012). Partial migration in fishes: Causes and consequences. Journal of Fish Biology, 81(2), 456–478. 10.1111/j.1095-8649.2012.03342.x 22803720

[ece39128-bib-0044] Cliff, G. , & Dudley, S. F. J. (1991). Sharks caught in the protective gill nets off natal, South‐Africa. 4. The Bull Shark Carcharhinus‐Leucas Valenciennes. South African Journal of Marine Science, 10, 253–270.

[ece39128-bib-0045] Copping, J. P. , Stewart, B. D. , McClean, C. J. , Hancock, J. , & Rees, R. (2018). Does bathymetry drive coastal whale shark (*Rhincodon typus*) aggregations? PeerJ, 6, e4904. 29900072 10.7717/peerj.4904 PMC5995094

[ece39128-bib-0046] Cornwallis, C. K. , & Birkhead, T. R. (2008). Plasticity in reproductive phenotypes reveals status‐specific correlations between behavioral, morphological, and physiological sexual traits. Evolution, 62, 1149–1161. 18266985 10.1111/j.1558-5646.2008.00346.x

[ece39128-bib-0047] Couturier, L. I. , Rohner, C. A. , Richardson, A. J. , Marshall, A. D. , Jaine, F. R. , Bennett, M. B. , Townsend, K. A. , Weeks, S. J. , & Nichols, P. D. (2013). Stable isotope and signature fatty acid analyses suggest reef manta rays feed on demersal zooplankton. PLoS One, 8(10), e77152. 24167562 10.1371/journal.pone.0077152 PMC3805558

[ece39128-bib-0048] Curtis, T. H. , McCandless, C. T. , Carlson, J. K. , Skomal, G. B. , Kohler, N. E. , Natanson, L. J. , Burgess, G. H. , Hoey, J. J. , & Pratt, H. L., Jr. (2014). Seasonal distribution and historic trends in abundance of white sharks, *Carcharodon carcharias*, in the western North Atlantic Ocean. PLoS One, 9(6), e99240. 24918579 10.1371/journal.pone.0099240 PMC4053410

[ece39128-bib-0049] Daly, R. , Froneman, P. W. , & Smale, M. J. (2013). Comparative feeding ecology of bull sharks (*Carcharhinus leucas*) in the coastal waters of the southwest Indian Ocean inferred from stable isotope analysis. PLoS One, 8(10), e78229. 10.1371/journal.pone.0078229 24205168 PMC3804608

[ece39128-bib-0050] Daly, R. , Smale, M. J. , Cowley, P. D. , & Froneman, P. W. (2014). Residency patterns and migration dynamics of adult bull sharks (*Carcharhinus leucas*) on the east coast of southern Africa. PLoS One, 9(10), e109357. 10.1371/journal.pone.0109357 25295972 PMC4190266

[ece39128-bib-0051] Daly, R. , Smale, M. J. , Singh, S. , Anders, D. , Shivji, M. , K. Daly, C. A. , Lea, J. S. , Sousa, L. L. , Wetherbee, B. M. , Fitzpatrick, R. , & Clarke, C. R. (2018). Refuges and risks: Evaluating the benefits of an expanded MPA network for mobile apex predators. Diversity and Distributions, 24(9), 1217–1230.

[ece39128-bib-0052] Dicken, M. L. , Hussey, N. E. , Christiansen, H. M. , Smale, M. J. , Nkabi, N. , Cliff, G. , & Wintner, S. P. (2017). Diet and trophic ecology of the tiger shark (*Galeocerdo cuvier*) from South African waters. PLoS One, 12(6), e0177897. 28594833 10.1371/journal.pone.0177897 PMC5464543

[ece39128-bib-0053] Dingle, H. (2014). Migration: The biology of life on the move. Oxford University Press.

[ece39128-bib-0054] Domeier, M. L. , & Nasby‐Lucas, N. (2008). Migration patterns of white sharks *Carcharodon carcharias* tagged at Guadalupe Island, Mexico, and identification of an eastern Pacific shared offshore foraging area. Marine Ecology Progress Series, 370, 221–237. 10.3354/meps07628

[ece39128-bib-0055] Domeier, M. L. , & Nasby‐Lucas, N. (2013). Two‐year migration of adult female white sharks (*Carcharodon carcharias*) reveals widely separated nursery areas and conservation concerns. Animal Biotelemetry, 1(1), 2.

[ece39128-bib-0056] Dulvy, N. K. , Fowler, S. L. , Musick, J. A. , Cavanagh, R. D. , Kyne, P. M. , Harrison, L. R. , Carlson, J. K. , Davidson, L. N. , Fordham, S. V. , Francis, M. P. , Pollock, C. M. , Simpfendorfer, C. A. , Burgess, G. H. , Carpenter, K. E. , Compagno, L. J. , Ebert, D. A. , Gibson, C. , Heupel, M. R. , Livingstone, S. R. , … White, W. T. (2014). Extinction risk and conservation of the world's sharks and rays. eLife, 3, e00590. 10.7554/eLife.00590 24448405 PMC3897121

[ece39128-bib-0057] Ebert, D. (1996). Biology of the sevengill shark *Notorynchus cepedianus* (Peron, 1807) in the temperate coastal waters of southern Africa. South African Journal of Marine Science, 17(1), 93–103.

[ece39128-bib-0058] Eggeman, S. L. , Hebblewhite, M. , Bohm, H. , Whittington, J. , & Merrill, E. H. (2016). Behavioural flexibility in migratory behaviour in a long‐lived large herbivore. Journal of Animal Ecology, 85, 785–797. 26790111 10.1111/1365-2656.12495

[ece39128-bib-0059] Espinoza, M. , Heupel, M. R. , Tobin, A. J. , & Simpfendorfer, C. A. (2016). Evidence of partial migration in a large coastal predator: Opportunistic foraging and reproduction as key drivers? PLoS One, 11(2), e0147608. 10.1371/journal.pone.0147608 26841110 PMC4740466

[ece39128-bib-0060] Ferreira, L. C. , Thums, M. , Heithaus, M. R. , Barnett, A. , Abrantes, K. G. , Holmes, B. J. , Zamora, L. M. , Frisch, A. J. , Pepperell, J. G. , Burkholder, D. , Vaudo, J. , Nowicki, R. , Meeuwig, J. , & Meekan, M. G. (2017). The trophic role of a large marine predator, the tiger shark *Galeocerdo cuvier* . Scientific Reports, 7, 7641. 10.1038/s41598-017-07751-2 28794497 PMC5550416

[ece39128-bib-0061] Ferreira, L. C. , Thums, M. , Meeuwig, J. J. , Vianna, G. M. , Stevens, J. , McAuley, R. , & Meekan, M. G. (2015). Crossing latitudes—Long‐distance tracking of an apex predator. PLoS One, 10(2), e0116916. 25671609 10.1371/journal.pone.0116916 PMC4324986

[ece39128-bib-0062] Fitzpatrick, R. , Thums, M. , Bell, I. , Meekan, M. G. , Stevens, J. D. , & Barnett, A. (2012). A comparison of the seasonal movements of tiger sharks and green turtles provides insight into their predator‐prey relationship. PLoS One, 7(12), e51927. 23284819 10.1371/journal.pone.0051927 PMC3526478

[ece39128-bib-0063] Flack, A. , Fiedler, W. , Blas, J. , Pokrovsky, I. , Kaatz, M. , Mitropolsky, M. , Aghababyan, K. , Fakriadis, I. , Makrigianni, E. , Jerzak, L. , Azafzaf, H. , Feltrup‐Azafzaf, C. , Rotics, S. , Mokotjomela, T. M. , Nathan, R. , & Wikelski, M. (2016). Costs of migratory decisions: A comparison across eight white stork populations. Science Advances, 2(1), e1500931. 10.1126/sciadv.1500931 26844294 PMC4737271

[ece39128-bib-0064] Francis, M. P. , Shivji, M. S. , Duffy, C. A. , Rogers, P. J. , Byrne, M. E. , Wetherbee, B. M. , Tindale, S. C. , Lyon, W. S. , & Meyers, M. M. (2019). Oceanic nomad or coastal resident? Behavioural switching in the shortfin mako shark (*Isurus oxyrinchus*). Marine Biology, 166(1), 5.

[ece39128-bib-0065] Fu, A. L. , Hammerschlag, N. , Lauder, G. V. , Wilga, C. D. , Kuo, C. Y. , & Irschick, D. J. (2016). Ontogeny of head and caudal fin shape of an apex marine predator: The tiger shark (*Galeocerdo cuvier*). Journal of Morphology, 277(5), 556–564. 10.1002/jmor.20515 26869274

[ece39128-bib-0066] Fudickar, A. M. , Schmidt, A. , Hau, M. , Quetting, M. , & Partecke, J. (2013). Female‐biased obligate strategies in a partially migratory population. Journal of Animal Ecology, 82(4), 863–871. 23363245 10.1111/1365-2656.12052

[ece39128-bib-0067] Gallagher, A. J. , Wagner, D. N. , Irschick, D. J. , & Hammerschlag, N. (2014). Body condition predicts energy stores in apex predatory sharks. Conservation Physiology, 2(1), cou022. 27293643 10.1093/conphys/cou022 PMC4732496

[ece39128-bib-0068] Gaube, P. , Braun, C. D. , Lawson, G. L. , McGillicuddy, D. J. , Della Penna, A. , Skomal, G. B. , Fischer, C. , & Thorrold, S. R. (2018). Mesoscale eddies influence the movements of mature female white sharks in the Gulf Stream and Sargasso Sea. Scientific Reports, 8(1), 1–8. 29743492 10.1038/s41598-018-25565-8 PMC5943458

[ece39128-bib-0069] Geijer, C. K. A. , Notarbartolo di Sciara, G. , & Panigada, S. (2016). Mysticete migration revisited: Are Mediterranean fin whales an anomaly? Mammal Review, 46(4), 284–296. 10.1111/mam.12069

[ece39128-bib-0070] Glaus, K. B. J. , Brunnschweiler, J. M. , Piovano, S. , Mescam, G. , Genter, F. , Fluekiger, P. , & Rico, C. (2019). Essential waters: Young bull sharks in Fiji's largest riverine system. Ecology and Evolution, 9(13), 7574–7585. 10.1002/ece3.5304 31346423 PMC6635934

[ece39128-bib-0071] Goymann, W. , Spina, F. , Ferri, A. , & Fusani, L. (2010). Body fat influences departure from stopover sites in migratory birds: Evidence from whole‐Island telemetry. Biology Letters, 6(4), 478–481. 20164077 10.1098/rsbl.2009.1028 PMC2936206

[ece39128-bib-0072] Hammerschlag, N. , Bell, I. , Fitzpatrick, R. , Gallagher, A. J. , Hawkes, L. A. , Meekan, M. G. , Stevens, J. D. , Thums, M. , Witt, M. J. , & Barnett, A. (2016). Behavioral evidence suggests facultative scavenging by a marine apex predator during a food pulse. Behavioral Ecology and Sociobiology, 70(10), 1777–1788. 10.1007/s00265-016-2183-2

[ece39128-bib-0073] Hammerschlag, N. , Broderick, A. C. , Coker, J. W. , Coyne, M. S. , Dodd, M. , Frick, M. G. , Godfrey, M. H. , Godley, B. J. , Griffin, D. B. , Hartog, K. , Murphy, S. R. , Murphy, T. M. , Nelson, E. R. , Williams, K. L. , Witt, M. J. , & Hawkes, L. A. (2015). Evaluating the landscape of fear between apex predatory sharks and mobile sea turtles across a large dynamic seascape. Ecology, 96(8), 2117–2126. 26405737 10.1890/14-2113.1

[ece39128-bib-0074] Hammerschlag, N. , Gallagher, A. , & Lazarre, D. (2011). A review of shark satellite tagging studies. Journal of Experimental Marine Biology and Ecology, 398(1–2), 1–8.

[ece39128-bib-0075] Hammerschlag, N. , Gallagher, A. J. , Wester, J. , Luo, J. G. , & Ault, J. S. (2012). Don't bite the hand that feeds: Assessing ecological impacts of provisioning ecotourism on an apex marine predator. Functional Ecology, 26(3), 567–576. 10.1111/j.1365-2435.2012.01973.x

[ece39128-bib-0076] Hammerschlag, N. , Luo, J. G. , Irschick, D. J. , & Ault, J. S. (2012). A comparison of spatial and movement patterns between sympatric predators: Bull sharks (*Carcharhinus leucas*) and Atlantic tarpon (*Megalops atlanticus*). PLoS One, 7(9), e45958. 10.1371/journal.pone.0045958 23049904 PMC3458817

[ece39128-bib-0077] Hammerschlag, N. , Schmitz, O. J. , Flecker, A. S. , Lafferty, K. D. , Sih, A. , Atwood, T. B. , Gallagher, A. J. , Irschick, D. J. , Skubel, R. , & Cooke, S. J. (2019). Ecosystem function and services of aquatic predators in the Anthropocene. Trends in Ecology & Evolution, 34(4), 369–383. 10.1016/j.tree.2019.01.005 30857757

[ece39128-bib-0078] Hart, B. L. , & Hart, L. A. (2018). How mammals stay healthy in nature: The evolution of behaviours to avoid parasites and pathogens. Philosophical Transactions of the Royal Society B: Biological Sciences, 373, 20170205. 10.1098/rstb.2017.0205 PMC6000140 29866918

[ece39128-bib-0079] Hatase, H. , Omuta, K. , & Tsukamoto, K. (2010). Oceanic residents, neritic migrants: A possible mechanism underlying foraging dichotomy in adult female loggerhead turtles (*Caretta caretta*). Marine Biology, 157(6), 1337–1342. 10.1007/s00227-010-1413-9

[ece39128-bib-0080] Hays, G. C. , Rattray, A. , & Esteban, N. (2020). Addressing tagging location bias to assess space use by marine animals. Journal of Applied Ecology, 57(10), 1981–1987.

[ece39128-bib-0081] Hedrick, A. V. , & Temeles, E. J. (1989). The evolution of sexual dimorphism in animals: Hypotheses and tests. Trends in Ecology & Evolution, 4, 136–138. 21227335 10.1016/0169-5347(89)90212-7

[ece39128-bib-0082] Heithaus, M. R. (2001). The biology of tiger sharks, Galeocerdo cuvier, in Shark Bay, Western Australia: Sex ratio, size distribution, diet, and seasonal changes in catch rates. Environmental Biology of Fishes, 61(1), 25–36.

[ece39128-bib-0083] Heithaus, M. R. , Wirsing, A. J. , & Dill, L. M. (2012). The ecological importance of intact top‐predator populations: A synthesis of 15 years of research in a seagrass ecosystem. Marine and Freshwater Research, 63(11), 1039–1050. 10.1071/Mf12024

[ece39128-bib-0084] Heithaus, M. R. , Wirsing, A. J. , Dill, L. M. , & Heithaus, L. I. (2007). Long‐term movements of tiger sharks satellite‐tagged in Shark Bay, Western Australia. Marine Biology, 151(4), 1455–1461.

[ece39128-bib-0085] Heupel, M. R. , Simpfendorfer, C. A. , Espinoza, M. , Smoothey, A. F. , Tobin, A. , & Peddemors, V. (2015). Conservation challenges of sharks with continental scale migrations. Frontiers in Marine Science, 2, 1–7. 10.3389/fmars.2015.00012

[ece39128-bib-0086] Holmes, B. J. , Pepperell, J. G. , Griffiths, S. P. , Jaine, F. R. , Tibbetts, I. R. , & Bennett, M. B. (2014). Tiger shark (*Galeocerdo cuvier*) movement patterns and habitat use determined by satellite tagging in eastern Australian waters. Marine Biology, 161(11), 2645–2658.

[ece39128-bib-0087] Holyoak, M. , Casagrandi, R. , Nathan, R. , Revilla, E. , & Spiegel, O. (2008). Trends and missing parts in the study of movement ecology. Proceedings of the National Academy of Sciences of the United States of America, 105, 19060–19065. 19060194 10.1073/pnas.0800483105 PMC2614715

[ece39128-bib-0088] Humphries, N. E. , Queiroz, N. , Dyer, J. R. , Pade, N. G. , Musyl, M. K. , Schaefer, K. M. , Fuller, D. W. , Brunnschweiler, J. M. , Doyle, T. K. , Houghton, J. D. , Hays, G. C. , Jones, C. S. , Noble, L. R. , Wearmouth, V. J. , Southall, E. J. , & Sims, D. W. (2010). Environmental context explains Levy and Brownian movement patterns of marine predators. Nature, 465(7301), 1066–1069. 10.1038/nature09116 20531470

[ece39128-bib-0089] Humphries, N. E. , Weimerskirch, H. , Queiroz, N. , Southall, E. J. , & Sims, D. W. (2012). Foraging success of biological levy flights recorded in situ. Proceedings of the National Academy of Sciences of the United States of America, 109(19), 7169–7174. 10.1073/pnas.1121201109 22529349 PMC3358854

[ece39128-bib-0090] Hussey, N. E. , McCann, H. M. , Cliff, G. , Dudley, S. F. , Wintner, S. P. , & Fisk, A. T. (2012). Size‐based analysis of diet and trophic position of the white shark (*Carcharodon carcharias*) in South African waters. In M. L. Domeier (Ed.), Global perspectives on the biology and life history of the white shark (pp. 27–49). CRC Press.

[ece39128-bib-0091] Huveneers, C. , Apps, K. , Becerril‐García, E. E. , Bruce, B. , Butcher, P. A. , Carlisle, A. B. , Chapple, T. K. , Christiansen, H. M. , Cliff, G. , Curtis, T. H. , Daly‐Engel, T. S. , Dewar, H. , Dicken, M. L. , Domeier, M. L. , CAJ, D. , Ford, R. , Francis, M. P. , GCA, F. , Galván‐Magaña, F. , … Werry, J. M. (2018). Future research directions on the “Elusive” White shark. Frontiers in Marine Science, 5, 455. 10.3389/fmars.2018.00455

[ece39128-bib-0092] Jaine, F. , Rohner, C. , Weeks, S. , Couturier, L. , Bennett, M. , Townsend, K. A. , & Richardson, A. (2014). Movements and habitat use of reef manta rays off eastern Australia: Offshore excursions, deep diving and eddy affinity revealed by satellite telemetry. Marine Ecology Progress Series, 510, 73–86.

[ece39128-bib-0093] Jaine, F. R. , Couturier, L. I. , Weeks, S. J. , Townsend, K. A. , Bennett, M. B. , Fiora, K. , & Richardson, A. J. (2012). When giants turn up: Sighting trends, environmental influences and habitat use of the manta ray Manta alfredi at a coral reef. PLoS One, 7, e46170. 23056255 10.1371/journal.pone.0046170 PMC3463571

[ece39128-bib-0094] Jenni, L. , & Kéry, M. (2003). Timing of autumn bird migration under climate change: Advances in long–distance migrants, delays in short–distance migrants. Proceedings of the Royal Society of London. Series B: Biological Sciences, 270(1523), 1467–1471. 10.1098/rspb.2003.2394 PMC1691393 12965011

[ece39128-bib-0095] Joly, K. , Gurarie, E. , Sorum, M. S. , Kaczensky, P. , Cameron, M. D. , Jakes, A. F. , Borg, B. L. , Nandintsetseg, D. , Hopcraft, J. G. , Buuveibaatar, B. , Jones, P. F. , Mueller, T. , Walzer, C. , Olson, K. A. , Payne, J. C. , Yadamsuren, A. , & Hebblewhite, M. (2019). Longest terrestrial migrations and movements around the world. Scientific Reports, 9(1), 1–10. 31654045 10.1038/s41598-019-51884-5 PMC6814704

[ece39128-bib-0096] Jonker, R. M. , Eichhorn, G. , Van Langevelde, F. , & Bauer, S. (2010). Predation danger can explain changes in timing of migration: The case of the barnacle goose. PLoS One, 5(6), e11369. 20614027 10.1371/journal.pone.0011369 PMC2894857

[ece39128-bib-0097] Jorgensen, S. J. , Arnoldi, N. S. , Estess, E. E. , Chapple, T. K. , Ruckert, M. , Anderson, S. D. , & Block, B. A. (2012). Eating or meeting? Cluster analysis reveals intricacies of white shark (*Carcharodon carcharias*) migration and offshore behavior. PLoS One, 7(10), e47819. 10.1371/journal.pone.0047819 23144707 PMC3483152

[ece39128-bib-0098] Jorgensen, S. J. , Reeb, C. A. , Chapple, T. K. , Anderson, S. , Perle, C. , van Sommeran, S. , Fritz‐Cope, C. , Brown, A. C. , Klimley, A. P. , & Block, B. A. (2010). Philopatry and migration of Pacific white sharks. Proceedings of the Royal Society B: Biological Sciences, 277(1682), 679–688. 10.1098/rspb.2009.1155 PMC2842735 19889703

[ece39128-bib-0099] Kessel, S. T. , Elamin, N. A. , Yurkowski, D. J. , Chekchak, T. , Walter, R. P. , Klaus, R. , Hill, G. , & Hussey, N. E. (2017). Conservation of reef manta rays (Manta alfredi) in a UNESCO World Heritage Site: Large‐scale Island development or sustainable tourism? PLoS One, 12(10), e0185419. 29069079 10.1371/journal.pone.0185419 PMC5656316

[ece39128-bib-0100] Ketterson, E. D. , Fudickar, A. M. , Atwell, J. W. , & Greives, T. J. (2015). Seasonal timing and population divergence: When to breed, when to migrate. Current Opinion in Behavioral Sciences, 6, 50–58.

[ece39128-bib-0101] Ketterson, E. D. , & Nolan, V., Jr. (1976). Geographic variation and its climatic correlates in the sex ratio of eastern‐wintering dark‐eyed juncos (*Junco hyemalis hyemalis*). Ecology, 57(4), 679–693.

[ece39128-bib-0102] Kingsolver, J. G. , Pfennig, D. W. , & Servedio, M. R. (2002). Migration, local adaptation and the evolution of plasticity. Trends in Ecology & Evolution, 17(12), 540–541.

[ece39128-bib-0103] Kuang, F. , Coleman, J. T. , Hassell, C. J. , Leung, K.‐S. K. , Maglio, G. , Ke, W. , Cheng, C. , Zhao, J. , Zhang, Z.‐W. , & Ma, Z. (2020). Seasonal and population differences in migration of Whimbrels in the East Asian–Australasian Flyway. Avian Research, 11(1), 1–12.

[ece39128-bib-0104] Lank, D. B. , Butler, R. W. , Ireland, J. , & Ydenberg, R. C. (2003). Effects of predation danger on migration strategies of sandpipers. Oikos, 103(2), 303–319.

[ece39128-bib-0105] Lawson, C. L. , Halsey, L. G. , Hays, G. C. , Dudgeon, C. L. , Payne, N. L. , Bennett, M. B. , White, C. R. , & Richardson, A. J. (2019). Powering ocean giants: The energetics of shark and ray megafauna. Trends in Ecology & Evolution, 34(11), 1009–1021. 31375293 10.1016/j.tree.2019.07.001

[ece39128-bib-0106] Lea, J. S. , Wetherbee, B. M. , Queiroz, N. , Burnie, N. , Aming, C. , Sousa, L. L. , Mucientes, G. R. , Humphries, N. E. , Harvey, G. M. , Sims, D. W. , & Shivji, M. S. (2015). Repeated, long‐distance migrations by a philopatric predator targeting highly contrasting ecosystems. Scientific Reports, 5, 11202. 26057337 10.1038/srep11202 PMC4460898

[ece39128-bib-0107] Lea, J. S. , Wetherbee, B. M. , Sousa, L. L. , Aming, C. , Burnie, N. , Humphries, N. E. , Queiroz, N. , Sims, D. W. , & Shivji, M. S. (2018). Ontogenetic partial migration is associated with environmental drivers and influences fisheries interactions in a marine predator. ICES Journal of Marine Science, 75(4), 1383–1392.

[ece39128-bib-0108] Lea, J. S. E. , Humphries, N. E. , Clarke, C. R. , & Sims, D. W. (2015). To Madagascar and back: Long‐distance, return migration across open ocean by a pregnant female bull shark *Carcharhinus leucas* . Journal of Fish Biology, 87(6), 1313–1321. 10.1111/jfb.12805 26511427

[ece39128-bib-0109] Lee, K. , Smoothey, A. , Harcourt, R. , Roughan, M. , Butcher, P. , & Peddemors, V. (2019). Environmental drivers of abundance and residency of a large migratory shark, *Carcharhinus leucas*, inshore of a dynamic western boundary current. Marine Ecology Progress Series, 622, 121–137.

[ece39128-bib-0110] Lipscombe, R. S. , Spaet, J. L. Y. , Scott, A. , Lam, C. H. , Brand, C. P. , & Butcher, P. A. (2020). Habitat use and movement patterns of tiger sharks (Galeocerdo cuvier) in eastern Australian waters. ICES Journal of Marine Science, 77(7–8), 3127–3137. 10.1093/icesjms/fsaa212

[ece39128-bib-0111] Lowe, C. G. , Wetherbee, B. M. , Crow, G. L. , & Tester, A. L. (1996). Ontogenetic dietary shifts and feeding behavior of the tiger shark, *Galeocerdo cuvier*, in Hawaiian waters. Environmental Biology of Fishes, 47(2), 203–211.

[ece39128-bib-0112] MacNeill, M. A. , Skomal, G. B. , & Fisk, A. T. (2005). Stable isotopes from multiple tissues reveal diet switching in sharks. Marine Ecology Progress Series, 302, 199–206. 10.3354/meps302199

[ece39128-bib-0113] Malpica‐Cruz, L. , Herzka, S. Z. , Sosa‐Nishizaki, O. , & Escobedo‐Olvera, M. A. (2013). Tissue‐specific stable isotope ratios of shortfin mako (*Isurus oxyrinchus*) and white (*Carcharodon carcharias*) sharks as indicators of size‐based differences in foraging habitat and trophic level. Fisheries Oceanography, 22(6), 429–445.

[ece39128-bib-0114] Marshall, A. , & Bennett, M. (2010). Reproductive ecology of the reef manta ray *Manta alfredi* in southern Mozambique. Journal of Fish Biology, 77(1), 169–190. 20646146 10.1111/j.1095-8649.2010.02669.x

[ece39128-bib-0115] Matich, P. , Heithaus, M. R. , & Layman, C. A. (2011). Contrasting patterns of individual specialization and trophic coupling in two marine apex predators. Journal of Animal Ecology, 80(1), 294–305. 10.1111/j.1365-2656.2010.01753.x 20831730

[ece39128-bib-0116] Mayr, E. (1963). Animal species and evolution (p. 797). Harvard University Press.

[ece39128-bib-0117] Meiri, S. , & Dayan, T. (2003). On the validity of Bergmann's rule. Journal of Biogeography, 30, 331–351.

[ece39128-bib-0118] Meyer, C. G. , Anderson, J. M. , Coffey, D. M. , Hutchinson, M. R. , Royer, M. A. , & Holland, K. N. (2018). Habitat geography around Hawaii's oceanic islands influences tiger shark (*Galeocerdo cuvier*) spatial behaviour and shark bite risk at ocean recreation sites. Scientific Reports, 8(1), 1–18. 29563552 10.1038/s41598-018-23006-0 PMC5862960

[ece39128-bib-0119] Meyer, C. G. , Papastamatiou, Y. P. , & Holland, K. N. (2010). A multiple instrument approach to quantifying the movement patterns and habitat use of tiger (*Galeocerdo cuvier*) and Galapagos sharks (*Carcharhinus galapagensis*) at French Frigate Shoals, Hawaii. Marine Biology, 157(8), 1857–1868.

[ece39128-bib-0120] Moorhead, S. G. , Gallagher, A. J. , Merly, L. , & Hammerschlag, N. (2021). Variation of body condition and plasma energy substrates with life stage, sex, and season in wild‐sampled nurse sharks *Ginglymostoma cirratum* . Journal of Fish Biology, 98(3), 680–693. 33161578 10.1111/jfb.14612

[ece39128-bib-0121] Nasby‐Lucas, N. , Dewar, H. , Lam, C. H. , Goldman, K. J. , & Domeier, M. L. (2009). White shark offshore habitat: A behavioral and environmental characterization of the eastern pacific shared offshore foraging area. PLoS One, 4(12), e8163. 10.1371/journal.pone.0008163 20011032 PMC2780721

[ece39128-bib-0122] Nasby‐Lucas, N. , Dewar, H. , Sosa‐Nishizaki, O. , Wilson, C. , Hyde, J. R. , Vetter, R. D. , Wraith, J. , Block, B. A. , Kinney, M. J. , Sippel, T. , Holts, D. B. , & Kohin, S. (2019). Movements of electronically tagged shortfin mako sharks (*Isurus oxyrinchus*) in the eastern North Pacific Ocean. Animal Biotelemetry, 7(1), 1–26.

[ece39128-bib-0123] Nathan, R. , Getz, W. M. , Revilla, E. , Holyoak, M. , Kadmon, R. , Saltz, D. , & Smouse, P. E. (2008). A movement ecology paradigm for unifying organismal movement research. Proceedings of the National Academy of Sciences of the United States of America, 105(49), 19052–19059. 10.1073/pnas.0800375105 19060196 PMC2614714

[ece39128-bib-0124] Newton, I. , & Dale, L. (1996). Relationship between migration and latitude among west European birds. Journal of Animal Ecology, 65, 137–146.

[ece39128-bib-0125] Niella, Y. , Smoothey, A. F. , Peddemors, V. , & Harcourt, R. (2020). Predicting changes in distribution of a large coastal shark in the face of the strengthening East Australian Current. Marine Ecology Progress Series, 642, 163–177. 10.3354/meps13322

[ece39128-bib-0126] Papastamatiou, Y. , & Lowe, C. (2012). An analytical and hypothesis‐driven approach to elasmobranch movement studies. Journal of Fish Biology, 80(5), 1342–1360. 22497387 10.1111/j.1095-8649.2012.03232.x

[ece39128-bib-0127] Papastamatiou, Y. P. , Meyer, C. G. , Carvalho, F. , Dale, J. J. , Hutchinson, M. R. , & Holland, K. N. (2013). Telemetry and random‐walk models reveal complex patterns of partial migration in a large marine predator. Ecology, 94(11), 2595–2606. 10.1890/12-2014.1 24400511

[ece39128-bib-0128] Payne, N. L. , Meyer, C. G. , Smith, J. A. , Houghton, J. D. , Barnett, A. , Holmes, B. J. , Nakamura, I. , Papastamatiou, Y. P. , Royer, M. A. , Coffey, D. M. , Anderson, J. M. , Hutchinson, M. R. , Sato, K. , & Halsey, L. G. (2018). Combining abundance and performance data reveals how temperature regulates coastal occurrences and activity of a roaming apex predator. Global Change Biology, 24(5), 1884–1893. 29516588 10.1111/gcb.14088

[ece39128-bib-0129] Peel, L. R. , Stevens, G. M. , Daly, R. , Daly, C. A. K. , Lea, J. S. , Clarke, C. R. , Collin, S. P. , & Meekan, M. G. (2019). Movement and residency patterns of reef manta rays *Mobula alfredi* in the Amirante Islands, Seychelles. Marine Ecology Progress Series, 621, 169–184.

[ece39128-bib-0130] Pirog, A. , Ravigné, V. , Fontaine, M. C. , Rieux, A. , Gilabert, A. , Cliff, G. , Clua, E. , Daly, R. , Heithaus, M. R. , Kiszka, J. J. , Matich, P. , Nevill, J. E. G. , Smoothey, A. F. , Temple, A. J. , Berggren, P. , Jaquemet, S. , & Magalon, H. (2019). Population structure, connectivity, and demographic history of an apex marine predator, the bull shark *Carcharhinus leucas* . Ecology and Evolution, 9, 12980–13000. 10.1002/ece3.5597 31871624 PMC6912899

[ece39128-bib-0131] Pratt, A. C. , Smith, K. T. , & Beck, J. L. (2017). Environmental cues used by greater sage‐grouse to initiate altitudinal migration. The Auk: Ornithological Advances, 134(3), 628–643.

[ece39128-bib-0132] Queiroz, N. , Humphries, N. E. , Couto, A. , Vedor, M. , da Costa, I. , Sequeira, A. M. M. , Mucientes, G. , Santos, A. M. , Abascal, F. J. , Abercrombie, D. L. , Abrantes, K. , Acuña‐Marrero, D. , Afonso, A. S. , Afonso, P. , Anders, D. , Araujo, G. , Arauz, R. , Bach, P. , Barnett, A. , … Sims, D. W. (2019). Global spatial risk assessment of sharks under the footprint of fisheries. Nature, 572(7770), 461. 10.1038/s41586-019-1444-4 31340216

[ece39128-bib-0133] Ratcliffe, N. , Adlard, S. , Stowasser, G. , & McGill, R. (2018). Dietary divergence is associated with increased intra‐specific competition in a marine predator. Scientific Reports, 8, 1–10. 29717229 10.1038/s41598-018-25318-7 PMC5931528

[ece39128-bib-0134] Rizzo, L. Y. , & Schulte, D. (2009). A review of humpback whales' migration patterns worldwide and their consequences to gene flow. Journal of the Marine Biological Association of the United Kingdom, 89(5), 995–1002. 10.1017/S0025315409000332

[ece39128-bib-0135] Rogers, P. J. , Huveneers, C. , Page, B. , Goldsworthy, S. D. , Coyne, M. , Lowther, A. D. , Mitchell, J. G. , & Seuront, L. (2015). Living on the continental shelf edge: Habitat use of juvenile shortfin makos Isurus oxyrinchus in the Great Australian Bight, southern Australia. Fisheries Oceanography, 24(3), 205–218.

[ece39128-bib-0136] Scott‐Phillips, T. C. , Dickins, T. E. , & West, S. A. (2011). Evolutionary theory and the ultimate–proximate distinction in the human behavioral sciences. Perspectives on Psychological Science, 6, 38–47. 26162114 10.1177/1745691610393528

[ece39128-bib-0137] Shaw, A. K. (2016). Drivers of animal migration and implications in changing environments. Evolutionary Ecology, 30, 991–1007.

[ece39128-bib-0138] Shaw, A. K. (2020). Causes and consequences of individual variation in animal movement. Movement Ecology, 8, 1–12. 32099656 10.1186/s40462-020-0197-x PMC7027015

[ece39128-bib-0139] Shaw, A. K. , & Levin, S. A. (2011). To breed or not to breed: A model of partial migration. Oikos, 120(12), 1871–1879.

[ece39128-bib-0140] Sims, D. W. , Humphries, N. E. , Bradford, R. W. , & Bruce, B. D. (2012). Levy flight and Brownian search patterns of a free‐ranging predator reflect different prey field characteristics. The Journal of Animal Ecology, 81(2), 432–442. 10.1111/j.1365-2656.2011.01914.x 22004140

[ece39128-bib-0141] Skomal, G. , Braun, C. , Chisholm, J. , & Thorrold, S. (2017). Movements of the white shark Carcharodon carcharias in the North Atlantic Ocean. Marine Ecology Progress Series, 580, 1–16.

[ece39128-bib-0142] Smoothey, A. F. , Lee, K. A. , & Peddemors, V. M. (2019). Long‐term patterns of abundance, residency and movements of bull sharks (*Carcharhinus leucas*) in Sydney Harbour, Australia. Scientific Reports, 9, 1–16. 10.1038/s41598-019-54365-x 31827123 PMC6906466

[ece39128-bib-0143] Somveille, M. , Rodrigues, A. S. , & Manica, A. (2015). Why do birds migrate? A macroecological perspective. Global Ecology and Biogeography, 24, 664–674.

[ece39128-bib-0144] Spaet, J. L. Y. , Manica, A. , Brand, C. P. , Gallen, C. , & Butcher, P. A. (2020). Environmental conditions are poor predictors of immature white shark *Carcharodon carcharias* occurrences on coastal beaches of eastern Australia. Marine Ecology Progress Series, 653, 167–179. 10.3354/meps13488

[ece39128-bib-0145] Stehfest, K. M. , Patterson, T. A. , Barnett, A. , & Semmens, J. M. (2014). Intraspecific differences in movement, dive behavior and vertical habitat preferences of a key marine apex predator. Marine Ecology Progress Series, 495, 249–262.

[ece39128-bib-0146] Stewart, J. D. , Nuttall, M. , Hickerson, E. L. , & Johnston, M. A. (2018). Important juvenile manta ray habitat at Flower Garden Banks National Marine Sanctuary in the northwestern Gulf of Mexico. Marine Biology, 165(7), 1–8.

[ece39128-bib-0147] Stutchbury, B. J. , Gow, E. A. , Done, T. , MacPherson, M. , Fox, J. W. , & Afanasyev, V. (2011). Effects of post‐breeding moult and energetic condition on timing of songbird migration into the tropics. Proceedings of the Royal Society B: Biological Sciences, 278(1702), 131–137. 10.1098/rspb.2010.1220 PMC2992728 20659932

[ece39128-bib-0148] Sulikowski, J. A. , Wheeler, C. R. , Gallagher, A. J. , Prohaska, B. K. , Langan, J. A. , & Hammerschlag, N. (2016). Seasonal and life‐stage variation in the reproductive ecology of a marine apex predator, the tiger shark *Galeocerdo cuvier*, at a protected female‐dominated site. Aquatic Biology, 24(3), 175–184.

[ece39128-bib-0149] Tillett, B. , Meekan, M. , Field, I. , Thorburn, D. , & Ovenden, J. (2012). Evidence for reproductive philopatry in the bull shark *Carcharhinus leucas* . Journal of Fish Biology, 80(6), 2140–2158. 22551174 10.1111/j.1095-8649.2012.03228.x

[ece39128-bib-0150] Tombre, I. M. , Høgda, K. A. , Madsen, J. , Griffin, L. R. , Kuijken, E. , Shimmings, P. , Rees, E. , & Verscheure, C. (2008). The onset of spring and timing of migration in two arctic nesting goose populations: The pink‐footed goose *Anser bachyrhynchus* and the barnacle goose *Branta leucopsis* . Journal of Avian Biology, 39(6), 691–703.

[ece39128-bib-0151] Vardanis, Y. , Klaassen, R. H. , Strandberg, R. , & Alerstam, T. (2011). Individuality in bird migration: Routes and timing. Biology Letters, 7(4), 502–505. 21307045 10.1098/rsbl.2010.1180 PMC3130220

[ece39128-bib-0152] Vaudo, J. J. , Byrne, M. E. , Wetherbee, B. M. , Harvey, G. M. , & Shivji, M. S. (2017). Long‐term satellite tracking reveals region‐specific movements of a large pelagic predator, the shortfin mako shark, in the western North Atlantic Ocean. Journal of Applied Ecology, 54(6), 1765–1775.

[ece39128-bib-0153] Vedor, M. , Queiroz, N. , Mucientes, G. , Couto, A. , Costa, I. D. , Santos, A. D. , Vandeperre, F. , Fontes, J. , Afonso, P. , Rosa, R. , Humphries, N. E. , & Sims, D. W. (2021). Climate‐driven deoxygenation elevates fishing vulnerability for the ocean's widest ranging shark. eLife, 10, e62508. 10.7554/eLife.62508 33461659 PMC7815312

[ece39128-bib-0154] Weng, K. C. , Boustany, A. M. , Pyle, P. , Anderson, S. D. , Brown, A. , & Block, B. A. (2007). Migration and habitat of white sharks (*Carcharodon carcharias*) in the eastern Pacific Ocean. Marine Biology, 152(4), 877–894. 10.1007/s00227-007-0739-4

[ece39128-bib-0155] Werry, J. M. , Lee, S. Y. , Otway, N. M. , Hu, Y. , & Sumpton, W. (2011). A multi‐faceted approach for quantifying the estuarine‐nearshore transition in the life cycle of the bull shark, *Carcharhinus leucas* . Marine and Freshwater Research, 62(12), 1421–1431. 10.1071/Mf11136

[ece39128-bib-0156] Werry, J. M. , Planes, S. , Berumen, M. L. , Lee, K. A. , Braun, C. D. , & Clua, E. (2014). Reef‐fidelity and migration of tiger sharks, Galeocerdo cuvier, across the Coral Sea. PLoS One, 9(1), e83249. 24421879 10.1371/journal.pone.0083249 PMC3885424

[ece39128-bib-0157] Whitney, N. M. , & Crow, G. L. (2007). Reproductive biology of the tiger shark (Galeocerdo cuvier) in Hawaii. Marine Biology, 151(1), 63–70.

[ece39128-bib-0158] Williams, G. , Andrews, K. S. , Katz, S. , Moser, M. L. , Tolimieri, N. , Farrer, D. , & Levin, P. (2012). Scale and pattern of broadnose sevengill shark Notorynchus cepedianus movement in estuarine embayments. Journal of Fish Biology, 80(5), 1380–1400. 22497389 10.1111/j.1095-8649.2011.03179.x

[ece39128-bib-0159] Williams, G. D. , Andrews, K. S. , Farrer, D. A. , Bargmann, G. G. , & Levin, P. S. (2011). Occurrence and biological characteristics of broadnose sevengill sharks (*Notorynchus cepedianus*) in Pacific Northwest coastal estuaries. Environmental Biology of Fishes, 91(4), 379–388.

