# Supplementary material for: The role of context in elucidating drivers of animal movement
Source: Ecol Evol. 2022 Jul 24;12(7):e9128. doi: 10.1002/ece3.9128 (PMC9309038; doi:10.1002/ece3.9128)
Supplement: Supplementary file 1 — Appendix S1 [file ECE3-12-e9128-s001.docx]

**The role of context in elucidating drivers of animal movement**

**Supplemental material:**

**Drivers of movement and intra-specific variability in elasmobranchs, extended discussion from Table 1 and Table 2 of main body (See bottom of document for both full tables)**

**Order: Lamniformes**

**Basking shark** (*Cetorhinus maximus*):

Basking sharks exhibit high variability in movement patterns. In the Eastern Atlantic some individuals remain in waters off the United Kingdom during winter while others move to waters off Spain, Portugal and North Africa (Doherty et al. 2017). Skomal et al. 2009 suggested that stable oceanographic conditions in the North-East Atlantic, driven by the Gulf Stream, result in year-round favourable conditions for basking sharks. This could explain why only some basking sharks leave UK waters in winter (Doherty et al. 2017). Basking sharks in the North-West Atlantic are subject to more dramatic seasonal environmental changes and have been shown to migrate latitudinally crossing the equator into the Southern hemisphere (Skomal et al. 2009).

Basking sharks use coastal and off-shore areas suggesting plasticity in resource use and tolerance to a variety of oceanographic settings. One tagged individual migrated longitudinally between the North-East and North-West Atlantic (Gore et al. 2008). The movements of this shark coincided with high upwelling and resulting plankton abundance at the destination (Gore et al. 2008). Furthermore migrating basking sharks use foraging tactics that enhance encounter rates with areas of high productivity and complex prey-species compositions (Sims et al. 2006). Overall, basking shark aggregations are related to high plankton density (Crowe et al. 2018).

Sims et al. 2000 observed court ship behaviour at feeding aggregations and proposed that court-ship is initiated at prey-rich foraging sites. Generally, reproductive biology of the basking shark is poorly understood (only one captured pregnant female of to date) and although hypothesized, no information on movements to parturition sites exists (Sims et al. 2000).

In summary, although reproduction and thermoregulation have been suggested as drivers for movement, no data exists to confirm either as a driver of movement. However, large-scale oceanographic conditions resulting in prey availability appear to be a driver for basking shark migrations.

**Grey nurse shark** (*Carcharias taurus*)

Movement patterns of grey nurse sharks are complex and variable among geographical locations (Lucifora et al. 2002, Smale 2002, Bansemer and Bennett, 2011, Otway and Ellis 2011, Smale et al. 2012, Kneebone et al. 2014, Teter et al. 2015). Reproduction has been identified as a key driver for this species in all studied locations, however reproductive strategies appear to differ regionally:

Along the East Coast of the USA, seasonal north-sound movements are evident in most demographics, except pregnant females which remain at lower, warmer latitudes in summer (Teter et al. 2015). At higher latitudes, some tagged females migrated off-shore instead of along the coast (Teter et al. 2015). Off-shore moving females are of a narrow size range constituting nearly or recently mature individuals (Teter et al. 2015). Teter et al. 2015 speculated that these females could move off-shore to avoid male harassment and delay pregnancy. Juveniles occupy high-latitude nursery areas in summer and undergo large-scale southward migrations to lower latitudes in winter (Kneebone et al. 2012, 2014, Teter et al. 2015).

In South America, movement patterns are similar to that of the East Coast of the USA (Lucifora et al. 2002). Both sexes and most size classes undergo seasonal north-south movements, while pregnant females are absent at high latitude summering areas (Lucifora et al. 2002). At those sites, sex ratio is 2:1, males to females, suggesting that 50% of females remains at low latitudes during the summer (Lucifora et al. 2002). This indicates a bi-annual reproductive cycle for females, where pregnant individuals remain at low latitudes (Lucifora et al. 2002). Mating is thought to occur at cooler, high latitude areas in South America, which is in contrast to the East Coast of the USA where mating is thought to occur at warmer latitudes (Lucifora et al. 2002, Teter et al. 2015).

In Australia and Southern Africa movement patterns are distinct from South America and the USA. Here, pregnant females undergo, predictable, repeated long-distance migrations (Bansemer and Bennett 2011, Otway and Ellis 2011). Mating occurs at colder, higher latitudes, after which females migrate to warm water gestation sites at lower latitudes and then return to colder waters to give birth (Smale 2002, Bansemer and Bennett 2011, Otway and Ellis 2011). In contrast to the East Coast of the USA, juveniles in South Africa and Australia do not appear to migrate but remain in nursery areas for up to 5 years (Smale 2002, Bansemer and Bennett 2011, Otway and Ellis 2011).

Temperature and resource availability also likely drive seasonal movements in most regions (Smale et al. 2012, Kneebone et al. 2012, 2014, Teter et al. 2015). Off-shore movements have been proposed to occur in order to exploit productive areas and eddy systems (Teter et al. 2015). Additionally, seasonal habitat use by migratory juveniles along the East Coast of the USA is likely driven by predator avoidance and resource availability (Kneebone et al. 2012, 2014).

**Mako shark** (*Isurus oxyrinchus*):

Mako sharks are a globally occurring, pelagic species that undergoes round-trip migrations of up to 25,000 km (Rogers et al. 2015). Movement in some regions occurs in a seasonal North-South manner, suggesting that temperature could shape movement. However, mako sharks are endothermic and high variability is evident with some sharks remaining in higher latitudes during winter (Rogers et al. 2015) (Francis et al. 2019). Additionally, mako sharks have been shown to experience broad temperature ranges (11-31˚C) (Nasby-Lucas et al. 2019), indicating that temperature in some areas is not a limiting factor for mako sharks and other factors like food availability may be more important.

Tagging studies in the Gulf of Mexico, North Atlantic, Australia and New Zealand show high variability in movement patterns with resident and migratory behavioural states (Vaudo et al. 2017, Byrne et al. 2019, Francis et al. 2019). Generally, resident types are associated with stable environmental conditions and year-round productivity (Rogers et al. 2015). Whereas, migrants occupy areas of high spatio-temporal variability of environmental conditions (e.g. temperature) and food availability (Vaudo et al. 2017, Byrne et al. 2019). For example, differing environmental factors appear to be responsible for variation of movement in different populations of mako sharks in the Western North Atlantic (WNA) and the Gulf of Mexico (GOM) (Vaudo et al. 2017). Individuals in the GOM moved considerably less than conspecifics in the WNA. Information on contextual factors such as local current regimes, magnitude of seasonal environmental change and resulting productivity from both study sites was used to demonstrate that conditions are more stable across space and time in the GOM (Vaudo et al. 2017). This relative oceanographic stability appears to negate the need to move over large distances and track patchily distributed resources compared to the WNA (Vaudo et al. 2017). Similarly, mako shark movement in the Eastern Pacific is also highly variable and relates to changing oceanic conditions causing seasonal spikes in productivity supporting large marine predators and prey items to overlap (Nasby-Lucas et al. 2019).

Knowledge on mako shark reproduction in correlation with migration is lacking. However, females might move to birthing grounds as some areas support high numbers of juvenile mako sharks (Nasby-Lucas et al. 2019). Additionally, direct temperature effects on mako shark movements are also poorly understood. However, globally, mako shark movement strategies are likely influenced by large-scale oceanographic conditions and resource availability. Due to high variability, mako shark movements appear context dependent, with varying oceanographic conditions shaping differences in movement patterns. A context-focused approach can help further elucidate the drivers of movement across and within regions (Vaudo et al. 2017).

**Porbeagle shark** (*Lamna nasus*):

Porbeagle sharks undergo large-scale migrations in the Atlantic and South Pacific (Pade et al. 2009, Saunders et al. 2011, Francis et al. 2015, Biais et al. 2017). In the United Kingdom, seasonal aggregations of porbeagles overlap with large schools of Atlantic mackerel (*Scomber scombrus*), a known prey source (Pade et al. 2009). Aggregations may therefore be linked to food availability (Pade et al. 2009). Northward movements by porbeagle sharks in this area have been observed in winter. As porbeagle sharks are endothermic, they may have an advantage over ectothermic predators by exploiting high latitudes in winter (Pade et al. 2009, Saunders et al. 2011). Therefore, low winter temperatures are unlikely to be a direct driver for porbeagle migrations.

One tagged porbeagle shark showed longitudinal, trans-Atlantic movements (Cameron et al. 2018), similar to the movements of a tagged basking shark in the same region (Gore et al. 2008). In basking sharks, it has been proposed that feeding opportunities drive such trans-Atlantic movements, which could also apply to porbeagle sharks (Gore et al. 2008).

In the Bay of Biscay, France sub-adults and adult females generally undergo cyclic seasonal migrations regardless of high variability in individual movement paths and destinations (Biais et al. 2017). These cyclic migrations are characterised by northward movements in summer and autumn, further northward or westward movements in winter, and then southward movements in late winter and spring (Biais et al. 2017). Northward movements into the North Sea and Norwegian waters overlap with spawning aggregations of herring (*Clupea harengus*). Herring are the dominant prey species in porbeagle stomachs in the region (Gauld 1989). Southward movements in spring and summer, back to the Bay of Biscay, overlap in time and space with high abundance of horse mackerel (*Trachurus trachurus*) and blue whiting (*Micromesistius poutassou*). These two species are the dominant prey of porbeagles in the Bay of Biscay in spring and summer (Biais et al. 2017). This suggests that porbeagle sharks in the north-east Atlantic undergo a large-scale cyclic migration that tracks different prey aggregations across seasons. In the Gulf of St. Lawrence, Canada porbeagle sharks overlap seasonally with eel migrations and eels have been found in porbeagle stomachs in the region (Béguer-Pon et al. 2012), further suggesting porbeagle sharks movements are driven by prey aggregations.

Females tagged in temperate Canadian waters made extensive movements into the subtropical Sargasso Sea (North-West Atlantic) while males and immature individuals remained in temperate waters (Campana et al. 2010). Movements to the Sargasso Sea occur during known parturition times for porbeagle sharks (Campana et al. 2010). Therefore the area could be a parturition site for females (Campana et al. 2010). In the North-East Atlantic, pregnant females and young-of-the-year spatially overlap in the Bay of Biscay and the Celtic Sea (Biais et al. 2017). Here, females and their offspring appear to share the same feeding habitats and occur simultaneously, in contrast to the proposed breeding system in the North-West Atlantic (Campana et al. 2010). Therefore, migration for parturition may be context-dependent in this species.

As seen in the Atlantic, porbeagle sharks occupy high latitudes in winter in the South Pacific, adding further evidence to the hypothesis that endothermy in porbeagle sharks enables seasonal exploitation of areas not accessible to ectothermic shark species (Francis et al. 2015). However, similar to the North-West Atlantic, some females migrate to lower latitudes in winter which may be linked to parturition as pupping peaks there in June-July (Campana et al. 2010, Francis et al. 2015).

Although common patterns are evident, the porbeagle sharks demonstrates high variability in movement patterns across and within populations. Seasonal prey aggregations appear to be an important factor in driving cyclic movements in the Atlantic. Reproductive movements seem to be absent in the north-east Atlantic (Biais et al. 2017), while females could be undergoing large-scale movements to parturition sites in the north-west Atlantic and Pacific (Campana et al. 2010, Francis et al. 2015). Reasons for such regional differences in reproductive ecology remain unknown. A context-focused approach may aid in elucidating the drivers behind these patterns.

**Salmon shark** (*Lamna ditropis*):

The endothermic salmon shark, shows high seasonal summer fidelity to areas in the North-East Pacific where salmon runs occur (Hulbert et al. 2005, Weng et al. 2005). A large salmon shark aggregation is found along the coast of British Columbia, Canada, where sharks are thought to intercept migrating salmon (Williams et al. 2010). Salmon sharks also show high seasonal abundance in coastal areas off Alaska, USA in summer (Hulbert et al. 2005, Weng et al. 2005). At these locations, stomach content analysis confirmed salmon as a major prey item, although other fish and cephalopods are taken opportunistically (Hulbert et al. 2005). After salmon numbers decline in Alaska some sharks disperse and undergo directed movements while others remain, even throughout winter (Hulbert et al. 2005, Weng et al. 2005). In general, salmon sharks do not appear to respond directly to low temperatures, as their physiological abilities allow them to maintain a higher body temperature than the surrounding water (Weng et al. 2005). Studies show this species can tolerate temperatures between 2-8˚C (Weng et al. 2005). Remaining animals may feed on overwintering herring and walleye pollock (*Theragra chalcogramma)* (Weng et al. 2008). Individuals leaving coastal waters off Alaska, may be driven by a combination of lower prey density, resulting competition and reproduction (Hulbert et al. 2005). These Individuals possibly take advantage of later salmon runs occurring further south in British Columbia and the Pacific North-West (Hulbert et al. 2005). Indeed, Weng et al. 2008 proposed that Salmon sharks track salmon runs up and down the North American Coast, as spawning runs and juvenile salmon off-shore movements occur at different times in southern regions.

Large-scale migrations from Alaska to the California Current and Subtropical Gyre are also evident in female salmon sharks (Weng et al. 2008). These southern locations may play a role in salmon shark reproduction. Salmon sharks appear to give birth during southerly migrations, as young of year have been observed off California (Weng et al. 2008). Distinct regions within the California current and the subtropical Gyre may be parturition sites. However, area-restricted search behaviour suggests the California Current is also a foraging ground (Weng et al. 2008). Animals moving into the oligotrophic subtropical Gyre on the other hand, show shorter residency and rapid return migrations north, combined with longer residency times in high latitude regions, such as Alaska. This implies, that the subtropical Gyre is likely only used for parturition (Weng et al. 2008). Thus, salmon sharks may exhibit two reproductive strategies in the eastern Pacific. One, where females spend more time in Alaskan waters to feed, migrate to a parturition site in the oligotrophic Subtropical Gyre and then rapidly return north. And the other, where females spend less time in high-latitude areas, migrate to the California Current to give birth, but also use the area for feeding purposes followed by a return to Alaskan waters after longer residency times at lower latitudes (Weng et al. 2008).

Overall, salmon shark movements into high latitude areas of the Eastern Pacific are driven by high seasonal prey abundance (Hulbert et al. 2005). Additionally, reproduction may drive female movement to two locations at lower latitudes (Weng et al. 2008). Low temperature does not appear to directly drive salmon shark migrations (Weng et al. 2005).

**White shark** (*Carcharodon carcharias*):

Movement behaviour of white sharks has been studied extensively in multiple regions. In the Eastern Pacific, white sharks form two coastal aggregations (California coast, USA and Guadalupe Island, Mexico) and migrate seasonally to an off-shore area in the Pacific, named “the white shark café” (Nasby-Lucas et al. 2009). The two research groups that have studied white shark behaviours at the café differ in their opinions of what is driving the use of these off-shore waters. Nasby-Lucas et al. 2009 suggest that the seasonal migrations are related to feeding, as male sharks demonstrate repeated oscillatory dives at the Café that potentially overlap with vertical movement behaviour of prey (Nasby-Lucas et al. 2009, Jorgensen et al. 2012). Additionally, some sharks migrate from the café to the Hawaiian island chain coinciding with humpback whale (*Megaptera novaeangliae*) calving (Domeier and Nasby-Lucas 2008), suggesting that food availability may shape large-scale movements away from coastal sites. Contrary to this, a second research group (Carlisle et al. 2012) suggests that white shark migration to the cafe is influenced by reproduction. Stable isotope analysis of males suggested that while white sharks feed seasonally in coastal areas feeding rates in off-shore areas of the Eastern Pacific, like the café, are reduced (Carlisle et al. 2012). Carlisle et al. 2012 therefore proposed that feeding is of minor importance while aggregating in offshore areas. Based on the reproductive cycle of white sharks in the Eastern Pacific mating is believed to occur during peak presence in the café (Jorgensen et al. 2010). Furthermore, male and female diving and movement behaviour in the café overlaps, and the diving behaviour of males is indicative of a potential lek-mating system (Jorgensen et al. 2012). A lek mating system is comprised of aggregating males, competing to gain enhanced access to females.

Parturition may also play an important role in driving white shark movements. Large-scale movement among females is variable, possibly due to their 18-month gestation period, causing them to only visit coastal nurseries every two years (Domeier and Nasby-Lucas 2013). In addition, young-of-the-year white sharks are observed at certain coastal sites in Mexico and California, suggesting that giving birth in suitable areas is an important aspect of large-scale movements in white sharks (Weng et al. 2007).

In Australia, regional feeding opportunities have been put forward as a main motivation for migration (Bruce et al. 2006). Movement patterns of sharks overlap with seasonal aggregations of prey such as snapper and sharks show highly directional movements, interspersed by short periods of residency (Bruce et al. 2006). Bruce et al. 2006 suggest this to be a strategy to take advantage of changing local food availability. White sharks in South Australia demonstrate sex-differences in movement behaviour with females moving further off-shore and experiencing a narrower temperature range than males (Bradford et al. 2020). Generally, white sharks in this area are associated with complex, productive habitats that support known prey (Bradford et al. 2020). Additionally, white sharks demonstrate fidelity to locations along the east coast of Australia, regardless of seasonally changing environmental factors, providing further support that prey availability or reproductive activity are important drivers of white shark movements (Spaet et al. 2020a). Ontogenetic shifts in movement patterns are also evident in animals tagged off Australia (Spaet et al. 2020b). Coastal movements in Victoria and New South Wales are common, while larger juveniles and sub-adults expand their movements into pelagic regions to New Zealand, New Caledonia and Papua New Guinea (Spaet et al. 2020b). Tagged Australian juvenile and sub-adult white sharks are associated with cyclonic, productive cold-core eddies (Lee et al. 2021). This is in contrast to adult white sharks in the North Atlantic, which prefer anti-cyclonic eddies (Gaube et al. 2018).

In the North Atlantic, off the North-East Coast of the USA, white sharks spend summers in highly productive coastal areas. These areas have recently seen the rebound of pinniped populations which may attract white sharks (Skomal et al. 2017). When temperatures drop, sharks migrate south to Florida, South Carolina and Gulf of Mexico in late autumn (Skomal et al. 2017). These Areas to the south experience a peak in productivity in winter. The southward migration is staggered among individuals, suggesting that low temperatures may not directly drive movements (Skomal et al. 2017). Generally, the occupied temperature range for this species varies between regions (Skomal et al. 2017), suggesting temperature effects on white shark movement may be context dependent.

Skomal et al. 2017 observed mating scars in females at coastal sites off the North-Eastern USA, suggesting that mating could occur there, rather than in an off-shore area as proposed for the Pacific (Jorgensen et al. 2012). Off-shore movements are apparent in the Eastern Pacific, Australia, South Africa and the Northern Atlantic (Bonfil et al. 2005, Jorgensen et al. 2012, Domeier and Nasby-Lucas 2013, Skomal et al. 2017). However, while these movements are spatially and temporally well-defined in the Eastern Pacific, off-shore migrations in Australia, South Africa and the Atlantic follow less clear patterns and individual variation is high (Skomal et al. 2017, Spaet et al. 2020b, Lee et al. 2021).

Overall, feeding opportunities and reproduction appear to drive white shark movements globally. However, information is still lacking on intra-specific variability in use of off-shore areas.

**Order: Carcharhiniformes**

**Blacktip shark** (*Carcharhinus limbatus*):

Blacktip sharks undergo seasonal mass migrations along the Eastern Coast of the United States. These movements overlap with spawning of multiple baitfish species in the region (Kajiura and Tellman 2016). This migration is also associated with temperature changes (Kajiura and Tellman 2016). However, if temperature is a direct physiological driver of blacktip movement or is masked by temperature responses of prey, remains unknown (Kajiura and Tellman 2016). In South Africa, blacktip sharks exhibit sex-differences in movement and space use (Daly, unpublished data). While females show high site fidelity to aggregation sites and migrate occasionally, males are highly mobile tracking prey along the coast and are associated with the Sardine Run (Daly, unpublished data). Overall large-scale movement patterns in this species are not well understood and the extent to which blacktip sharks exhibit variability in movement across their range remains unknown.

**Blue shark** (*Prionace glauca*):

Blue sharks exhibit complex migration patterns with high seasonal variability, sex and size differences (e.g., Howey et al. 2017, Maxwell et al. 2019). The drivers for this variability remain largely unexplored. Being a cosmopolitan, pelagic species, the blue shark is capable of large-scale across-ocean basin migrations. Mark-recapture studies have shown that blue sharks can migrate between Australia and Southern Africa, Southern Africa and South America and Australia and Indonesia (da Silva et al. 2010, Stevens et al. 2010). The wide-open space of pelagic areas and patchily distributed resources may add to this complexity in pelagic shark movement patterns.

Satellite tagged individuals in the Atlantic, move towards the Gulf Stream (east coast USA) in winter showing distinct diving behaviour (Campana et al. 2011). Strong spatial overlap with prey species such as fish and squid suggests that the diving behaviour is a foraging tactic. Blue sharks may therefore migrate into the area to take advantage of increased prey numbers (Campana et al. 2011). Howey et al. 2017 found that blue sharks aggregate seasonally on the continental shelf off the North-Eastern United States. This area is high in primary production and likely functions as an important feeding ground in summer (Howey et al. 2017). Return migrations demonstrated site-fidelity to the area in some individuals (Howey et al. 2017). Mature and immature individuals were observed at the aggregation site, suggesting the driver of movement to the area is linked to resource availability rather than reproduction (Howey et al. 2017). Differences in movement behaviour between mature and immature animals were apparent (Howey et al. 2017). Immature individuals remained largely resident while mature males showed long-distance migrations. Mature females were absent from the tagging site (Howey et al. 2017). However, in this study, two mature females were tagged south in the Bahamas and exhibited fresh mating scars (Howey et al. 2017). These two individuals migrated to two different destinations, the Mid-Atlantic Ridge and Azores respectively. Howey et al. 2017 hypothesized that after a stopover in the Bahamas, pregnant females migrate across the Atlantic to parturition sites, such as the Azores. There, mature females arrive in spring and neonates are observed in summer (Vandeperre et al. 2014). Suggesting female blue sharks migrate to birthing grounds (also see da Silva et al. 2010). Near the Azores, blue shark presence is also associated with eddies and other oceanographic features that concentrate prey animals (Vandeperre et al. 2014), suggesting that parturition and feeding opportunities drive mature blue shark movement across the Atlantic.

Blue sharks in the North-East Pacific show differences in migratory behaviour between individuals tagged in pelagic and coastal habitats (Maxwell et al. 2019). This difference in movement patterns has been hypothesized to be related to differences in oceanographic factors between coastal and pelagic areas (Maxwell et al. 2019).

Overall, the complexity of global blue shark movements with strong regional, sex- and size-class differences prevents a clearer understanding of the drivers of movement in this species. However, seasonal food availability and reproduction likely contribute to movement patterns across the range.

**Bull shark** (*Carcharhinus leucas*):

Movement patterns and large scale migrations of bull sharks vary considerably among regions. In some regions such as Florida, bull sharks appear more localized with limited movement (Carlson et al. 2010, Hammerschlag et al. 2012), while in other regions (east coasts of Australia and southern Africa) bull sharks make large-scale return migrations (Daly et al. 2014, Heupel et al. 2015, Espinoza et al. 2016)

High variability is also evident within regions, such as the east coast of Australia. While some individuals remain resident on the central Great Barrier Reef (GBR), others make extensive return-migrations along the east coast (Heupel et al. 2015, Espinoza et al. 2016). Bull sharks tagged at higher latitudes, such as Sydney Harbour, migrate north into Queensland waters in winter, including the central GBR (Heupel et al. 2015, Espinoza et al. 2016, Lee et al. 2019). In contrast, bull sharks tagged on the central GBR do not move as far south as Sydney Harbour (Heupel et al. 2015). Seasonal space-use in both, the central GBR and Sydney Harbour have been attributed to food availability (Espinoza et al. 2016, Smoothey et al. 2016, Lee et al. 2019, Smoothey et al. 2019). Highest bull shark presence on the central GBR coincides with fish aggregations (e.g., mackerel spawning) (Espinoza et al. 2016), and the Sydney Harbour estuary is regarded as a highly productive area (Smoothey et al. 2016, 2019). While movements along the east coast of Australia may be influenced by resource availability, movement patterns are distinctively different between individuals tagged at the centre of bull shark distribution and individuals at the range limit, where larger migrations may be influenced by seasonal temperature changes, e.g. low winter temperatures.

Reproduction as a driver of movement has also been proposed for bull sharks (Lea et al. 2015, Espinoza et al. 2016). Globally, females utilise upstream reaches of river systems as nurseries (Heupel and Simpfendorfer 2011), therefore seasonal movements from off-shore feeding areas to upstream riverine pupping grounds are likely (Werry et al. 2011, Brunnschweiler and Barnett 2013, Glaus et al. 2019, Lee et al. 2019). A seemingly pregnant female tagged off the Seychelles demonstrated a large-scale migration of 2000 km towards a coastal region in Madagascar that has multiple rivers and estuaries (Lea et al. 2015). There, the shark occupied shallow waters less than 5m deep, indicating that this female may have given birth in a river system at the destination (Lea et al. 2015).

Limited genetic evidence suggests female natal philopatry in bull sharks (Tillett et al. 2012). This could produce further variability in bull shark movement patterns as different females utilise different rivers. If natal philopatry is widely evident in bull sharks, the extent of it may vary annually in some regions depending on available juvenile habitat. In Southern Africa, some river systems known to be bull shark pupping grounds, were closed off from the ocean during years of drought (Daly, personal communication). After extensive rainfall and re-connection of the rivers to the ocean, bull shark pups reappeared in these systems (Daly, personal communication).

Overall, research from multiple locations shows that temperature, food availability and reproduction may all play roles in driving bull shark movements. However, which specific factors drive the high variability among and within populations, remains poorly understood. A context-focused approach may help to shed more light on movement drivers and intra-specific variability in this species.

**Dusky shark** (*Carcharhinus obscurus*):

Dusky sharks can tolerate a relatively broad range of seasonal temperatures but winter movements occur in regions affected by low temperatures, suggesting that for some populations direct temperature effects are driving movement patterns (Hussey et al. 2009, Rogers et al. 2013). Thus, regional differences in temperature regimes may shape variability in dusky shark movements between animals at higher and lower latitudes.

Squid are an important food source for dusky sharks and movements overlap with squid spawning aggregations in South Africa (Hussey et al. 2009). Differences in foraging behaviour are also evident between sex and age classes for dusky sharks in South Africa suggesting differential movements between distant feeding grounds (Hussey et al. 2011). Furthermore, sharks undergoing large-scale movements along the continental shelf edge in the United States appear to track seasonal productivity (Hoffmayer et al. 2014). Overall, available information on diet and seasonal habitat use suggests that seasonal prey availability is an important driver for global dusky shark movements.

Large females are only caught in bather-protection nets in South Africa during the pupping season, which suggests dusky sharks use in-shore nursery grounds. Seasonal female migration may therefore be related to reproduction (Hussey et al. 2009). In Western Australia larger males and females migrate, while smaller size-classes remain more localized (Braccini et al. 2018). Additionally partial migration occurs, where only some large females undergo large-scale migration which is thought to be partially due to a bi- or even tri-annual breeding cycle of females migrating to parturition sites (Braccini et al. 2018).

Overall, dusky shark movements appear to be driven by food availability, reproduction and low winter temperatures. In addition, sex- and age-classes show distinct movements, suggesting individual context may play an important role in driving dusky shark movement behaviour.

**School shark** (*Galeorhinus galeus*):

Large-scale migrations are evident in School sharks from different ocean basins (McMillan et al. 2019, Thorburn et al. 2019). In Southern Australia, female school sharks exhibit partial migration (McMillan et al. 2019). While some females remain and pup in year-round productive areas of South Australia, some females migrate to the South-East, near Tasmania to pup (McMillan et al. 2019). The reasoning behind female partial migration remains unknown, but natal philopatry could play a role (McMillan et al. 2019). This species also likely exhibits a tri-annual breeding cycle which could also shape patterns of partial migration (Thorburn et al. 2019, Nosal et al. 2021). Aggregations by females in warmer waters around South Australia are thought to expedite embryonic growth and gestation (McMillan et al. 2019). Furthermore, seasonal south-eastward movements in Southern Australia overlap with spawning aggregations of prey (Rogers et al. 2017). Rogers et al. 2017 also suggested that movement patterns on the mid-outer shelf in Southern Australia may be driven by predation risk.

In the Eastern Pacific, off the US and Mexican Coast, female school sharks exhibit philopatric behaviour with a tri-annual migration pattern (Nosal et al. 2021). Females return to specific coastal areas which likely function as gestation sites. Presence at these sites is followed by movement to suggested nursery areas (Nosal et al. 2021).

Large-scale migrations have also been observed in the North-Atlantic, where school shark movements overlap with movement of prey species, such as mackerel (*Scomber scrombus*) (Thorburn et al. 2019). Additionally, seasonal movements from the North-Atlantic into the Mediterranean are evident where large females and juveniles overlap, suggesting that such large-scale movements may be for parturition (Colloca et al. 2019). Female partial migration has also been observed in this area (Thorburn et al. 2019). Differing reproductive stages and parturition sites for individual females may drive this pattern (Thorburn et al. 2019).

Overall, although movement studies are available from multiple regions, the key drivers of movement require further research. Food availability and foraging behaviour, as well as predator avoidance has been, at least partially, attributed to shaping movement patterns (Rogers et al. 2017, Thorburn et al. 2019). Additionally, movement of school sharks broadly corresponds to temperature changes and salinity gradients, but if those environmental parameters directly affect shark physiology or prey remains unknown (Jaureguizar et al. 2018). To date, most studies support the notion that philopatry to gestation and parturition sites drives female migration patterns globally.

**Tiger shark** (*Galeocerdo cuvier*):

Tiger sharks show complex migration patterns in a range of locations (e.g., Papastamatiou et al. 2013, Werry et al. 2014, Lea et al. 2015, Meyer et al. 2018).

Tiger shark abundance in Shark Bay, Western Australia correlated with abundance of specific prey animals, particularly dugongs and sea snakes (Heithaus 2001). Satellite tagged animals in the area demonstrated variable movement behaviour (Heithaus et al. 2007). Some individuals remained within Shark Bay, while one made off-shore movements before returning. Heithaus 2001 hypothesized that tiger sharks may use smaller areas at small temporal scales when prey density is high and move between patches of high food availability at larger temporal scales. One individual in the study moved from Shark Bay towards Southern Africa. Drivers for such longitudinal movements between these locations (also observed in white sharks, see Bonfil et al. 2005) remain unknown (Heithaus et al. 2007).

Tiger sharks from southern Africa showed limited coastal movements (Daly et al. 2018). Such small latitudinal movements may be the result of high localised resource availability (Daly et al. 2018). Similarly, tiger sharks along the East Coast of Australia move along a limited latitudinal range (Holmes et al. 2014). However, differences between animals tagged at the range limit, in Southern Queensland/New South Wales and North Queensland are evident (Holmes et al. 2014, Lipscombe et al. 2020, Barnett unpublished data]. Animals tagged at higher latitudes move larger latitudinal distances in accordance with seasonal temperature changes and eddy activity including excursions to seamounts and into off-shore waters (Holmes et al. 2014, Lipscombe et al. 2020). Animals tagged at lower latitudes, on the other hand show high individual variability with localised movements and residency as well as large-scale longitudinal movements to Pacific Islands and the Coral Sea (Fitzpatrick et al. 2012, Holmes et al. 2014, Barnett unpublished data]. For animals tagged in the Coral Sea, intra-specific variability in movement patterns is evident as males and sub-adults show high residency to oceanic islands, while females undergo large-scale movements, potentially to parturition sites (Werry et al. 2014). Overall, tiger sharks on the East Coast of Australia show varying movement strategies based on tagging latitude. This is similar to bull sharks who demonstrate differences in movement patterns between animals tagged at higher and lower latitudes (Heupel et al. 2015, Espinoza et al. 2016).

The restricted latitudinal movements observed in Southern Africa and parts of the Australian East Coast are in contrast to the large-scale latitudinal movements in the Northern Atlantic and Western Australia (Heithaus et al. 2007, Ferreira et al. 2015, Lea et al. 2015).

In the Atlantic, tiger sharks make large-scale (7500 km) round-trip migrations between Caribbean coral reef systems and high-latitude oceanic systems (Lea et al. 2015, 2018). This constitutes movement between highly disparate systems and demonstrates plasticity in tiger shark habitat use. At high latitudes tiger sharks overlap seasonally with prey such as turtles, tuna and billfish in summer (Hammerschlag et al. 2015, Lea et al. 2015), suggesting that north-ward movements are driven by increased prey abundance in pelagic systems. High fidelity to low-latitude coral reef systems may be driven by mating opportunities (Lea et al. 2015). Partial migration is also evident in this system (Lea et al. 2015). Juveniles remain localised at low latitudes, while larger individuals migrated extensively. Lea et al. 2018 showed that the effect of environmental factors on movement patterns differs by size class. Therefore, individual contextual factors such as size, condition, reproductive status and their interaction with environmental factors may play a role in shaping tiger shark movement patterns. This has also been shown in the Gulf of Mexico and Hawaii (Papastamatiou et al. 2013, Ajemian et al. 2020).

Papastamatiou et al. 2013 proposed that further variability in mature female movement in Hawaii may be due to skipped-breeding migrations. Although tiger shark nursery areas remain unknown, limited genetic evidence suggests females may demonstrate natal philopatry to pupping areas (Bernard et al. 2016, Pirog et al. 2019). Hammerschlag et al. 2012 also hypothesized that shallow warm-water areas around the Tiger Beach in the Bahamas could function as gestation sites for pregnant females. Ultra-sounds and hormone analysis added further evidence to this hypothesis (Sulikowski et al. 2016). This suggests that multiple aspects of reproductive strategy could drive movement in female tiger sharks.

In the Hawaiian Islands tiger sharks make inter-island and off-shore movements (Papastamatiou et al. 2013, Meyer et al. 2010, 2018). These directed movements are often followed by localised space use around seamounts, banks and other areas of high productivity, including albatross fledging sites (Meyer et al. 2018). Highly directed movements may indicate that experience plays a role in shaping tiger shark movement (Meyer et al. 2018).

However, tiger sharks in Hawaii also show high variability in movement patterns (Papastamatiou et al. 2013, Meyer et al. 2010, 2018). Only some individuals visit albatross fledging sites or undergo inter-island and off-shore movements. Most tiger sharks around Maui, for example, remain year-round, while sharks from other islands visit Maui seasonally (Meyer et al. 2018). The shelf off Maui constitutes one of the most productive systems of any Pacific Island area and may therefore support a large number of tiger sharks year-round (Meyer et al. 2018). Furthermore, visits by non-Maui-residents coincides with proposed breeding times for tiger sharks (Whitney and Crow 2007) and therefore could be driven by parturition and mating (Papastamatiou et al. 2013, Meyer et al. 2018).

Although tiger sharks are considered thermal generalists, their movements correlate with temperature changes in some regions, especially at the range limits (Holmes et al. 2014, Lipscombe et al. 2020) and activity levels are highest within a certain temperature niche (~22-24˚C) (Payne et al. 2018). In Hawaii and in the Atlantic, tiger shark occurrence is highest around ~23-27°C (Papastamatiou et al. 2013, Lea et al. 2018). Lea et al. 2018 suggested that temperature is an important predictor for overall tiger shark distribution while foraging opportunities and productivity may better explain shark occurrence within favourable temperature ranges (Lea et al. 2018). In contrast, around Raine Island, the world’s largest green turtle (*Chelonia mydas*) nesting site, some individual tiger sharks are exposed to temperatures substantially higher (over 30°C) than the temperature niche found in Lea et al. 2018 and Payne et al. 2018 for prolonged periods (Fitzpatrick et al. 2012). This suggests that localised behaviour despite sub-optimal temperatures may be context dependent. For example, abundant and easily available prey in the form of dead and weakened green turtles may create a trade-off that allows tiger sharks to remain in sub-optimal temperatures (Hammerschlag et al. 2016).

Overall, feeding opportunities appear to be important drivers for tiger shark movement. The high variability in tiger shark movement patterns may partially be explained by variable foraging strategies, which may exist across different regions and habitats in Australia, for example (Ferreira et al. 2017). If sharks focus on different prey and foraging habitats, individual movement patterns could differ as a result. Tiger sharks are regarded as generalist feeders which show diet diversification with increasing size (Lowe et al. 1996, Dicken et al. 2017). As sharks forage over a wider range of taxa throughout ontogeny, their movement patterns could expand as well. This may partially explain why adults range further than juveniles in some areas (Lea et al. 2015).

Globally, tiger shark movements are highly variable and complex. Partial migration is evident across the species range. Environmental, ecological and internal factors likely act in concert producing the observed complexity in tiger shark movements. Further analysis using a context-focused approach may be beneficial in classifying different movement strategies across populations and individuals.

**Great hammerhead** (*Sphyrna mokarran*):

Only limited information on movement behaviour is available for this species. Great Hammerhead sharks have shown large-scale migrations in the north-western Atlantic (Hammerschlag et al. 2011, Guttridge et al. 2017). These have been linked to changes in temperature and prey availability. Partial migration is evident where some individuals remain localised despite changes in temperature and food availability (Guttridge et al. 2017). Young of the year have been identified in potential nursery areas in the South-Eastern United States, suggesting that females may migrate to give birth in coastal nurseries (Barker et al. 2017). However, as spatio-temporal aspects of movement for this species are understudied, knowledge of movement drivers is lacking (Hammerschlag et al. 2011, Guttridge et al. 2017).

**Scalloped hammerhead** (*Sphyrna lewini*):

Scalloped hammerheads demonstrate variable movement strategies (Bessudo et al. 2011, Wells et al. 2018) and are often associated with oceanic seamounts forming large aggregations (Bessudo et al. 2011, Ketchum et al. 2014, Wells et al. 2018, Nalesso et al. 2019). Seasonal residency and small scale movements as well as large-scale migrations occur (Bessudo et al. 2011, Ketchum et al. 2014, Wells et al. 2018, Nalesso et al. 2019). While most satellite tracking studies for scalloped hammerheads are short in duration (<120 days) and do not cover a whole seasonal cycle (Bessudo et al. 2011, Wells et al. 2018, Nalesso et al. 2019), acoustic telemetry has demonstrated return migrations over larger time spans (Bessudo et al. 2011).

Limited movement was evident in the Gulf of Mexico (GOM) with only few across shelf-movements (Wells et al. 2018). In the GOM scalloped hammerheads were associated with the 200m isobath and with low chlorophyll-a levels, artificial structures and hard-bottom habitats. Additionally, individuals avoided areas of high nutrient influx, such as the Mississippi river mouth (Wells et al. 2018). Males and females were highly separated, with females spending more time off-shore (Wells et al. 2018). Presence in the GOM may be driven by bathymetric factors such as depth, habitat type and artificial structures rather than oceanographic factors such as temperature. In Southern Africa, where seasonal temperature changes are greater, seasonally declining scalloped hammerhead abundance has been attributed to low winter temperatures (Diemer et al. 2011). However, information on the relationship between direct temperature effects and other ecological and/or biological factors remains scarce.

High presence at archipelagos in Tropical Eastern Pacific (e.g. Galapagos, Malpelo, Cocos) coincides with seasonal fish abundance and current patterns (Bessudo et al. 2011, Ketchum et al. 2014) However, individual differences in movement behaviour are evident (Bessudo et al. 2011, Ketchum et al. 2014). More females undergo large-scale movements than males (Ketchum et al. 2014). Some sharks move between different archipelagos and movements can cover up to 1900 km (Bessudo et al. 2011, Ketchum et al. 2014). At Cocos Island, hammerheads show high residency but periods of longer absence and movements between archipelagos (Cocos, Galapagos, Malpelo) are common (Nalesso et al. 2019). After periods of absence, animals return to Cocos suggesting site fidelity (Nalesso et al. 2019). Animals tagged at Galapagos and Malpelo were detected at Cocos Island, however showed only short periods of residency, while animals from Cocos were rarely detected at other islands (Nalesso et al. 2019). This suggests that Cocos performs a specific function, e.g. as a stop-over or navigational point for animals migrating to the coast (Nalesso et al. 2019). Additionally, mating of scalloped hammerheads has been observed at Cocos (Salinas-de-Leone et al. 2017).

Speculation that females move to the coast of South and Central America as part of their reproductive cycle are supported by an increase in juvenile hammerheads in April and May at coastal sites in Costa Rica, Panama, Colombia and Ecuador and a decrease in adult hammerheads at Cocos, Galapagos and Malpelo in March (Bessudo et al. 2011, Nalesso et al. 2019). Shallow bays and coastal areas are also nursery areas for scalloped hammerheads elsewhere, e.g. Hawaiian Islands, Eastern Australia and Fiji (Duncan and Holland 2006, Yates et al. 2015, Marie et al. 2017). In addition, seemingly pregnant individuals moved away from Malpelo Island in March and April, further suggesting movement to parturition or nursery areas (Bessudo et al. 2011). In general, scalloped hammerheads show high intra-specific variability in movement patterns. Both seasonal oceanographic conditions shaping resource availability and reproduction appear to play roles in driving movement in this species. However, drivers of individual variability and differences in movement behaviour between archipelagos in the Eastern Pacific remain less well understood, making this species a good candidate for a context-focused approach.

**Order: Hexanchiformes**

**Sevengill shark** (*Notorynchus cepedianus*):

A suite of studies on broadnose sevengill sharks (*Notorynchus cepedianus)* in southern Tasmania, Australia provided insight into the drivers of migration into coastal systems (Barnett et al. 2010a,b,c,d, Abrantes and Barnett 2011, Barnett et al. 2011, Barnett and Semmens 2012, Awruch et al. 2014, Stehfest et al. 2014, Dudgeon et al. 2015). Tracking (acoustic and satellite telemetry), catch rates (recaptures) and stable isotope analysis of sevengill sharks revealed seasonal (spring-Autumn) site fidelity to coastal areas of Tasmania (Barnett et al. 2010c, Barnett et al. 2011, Abrantes and Barnett 2011, Stehfest et al. 2014). Sevengill sharks are apex predators in these coastal systems, and therefore habitat use/migration is likely not linked to sheltering from predation. Although most sevengill sharks leave coastal areas in winter (some make large-scale movements of up to 970 km to New South Wales), some females remain close to coastal areas, suggesting that temperature per se is not a key driver for movement (Barnett et al. 2011, Barnett et al. 2010dc). This has also been observed in New Zealand where sevengill sharks were still present during low winter temperatures (Housiaux et al. 2019). The absence of neonates and smaller juveniles (<100 cm) (Barnett et al. 2010d,c), low number of females with mating scars and reproductive status (e.g. no near-term pregnant individuals) ruled out reproduction as a driver for movement into the area (Awruch et al. 2014). Diet analysis and estimates of predator-prey abundance combined with similar movement patterns, seasonality, and high spatial overlap of predator and prey provide strong support that sevengill sharks move into coastal systems in Tasmania to take advantage of seasonally abundant prey resources Abrantes and Barnett 2011, Barnett et al. 2010a,b, Barnett and Semmens 2012. Here, a multi-method approach ruled out factors unlikely to be responsible for movement while narrowing down the most likely driver – high seasonal prey availability.

On the West Coast of the USA, sevengill sharks migrate along the coast and show high site fidelity to estuaries in summer (Williams et al. 2012). Here, seasonal migration to estuaries is also believed to be related to increased prey availability (Williams et al. 2011). In contrast to Australia (where males made large-scale migrations), females made large scale movements, including movements to Humboldt Bay and Mission Beach in California (650 km and 1800 km, respectively) which is a suspected nursery ground for the species (Williams et al. 2012) suggesting reproduction could be a movement driver for female sevengill sharks.

In summary, a wealth of information supports the notion that across the species range, movement into coastal bays and estuaries during summer occurs to take advantage of ample prey resources (Barnett and Semmens 2012). Temperature appears to play less of a role in sevengill shark movement. However, the role of reproduction and other individual factors driving movement differences requires further investigation in this species (Williams et al. 2012).

**Order: Orectolobiformes**

**Whale shark** (*Rhincodon typus*):

Whale sharks aggregate seasonally in high numbers at multiple sites around the globe (Norman et al. 2017). Aggregation sites generally show three characteristics: (1) shallow, warm waters, (2) proximity to deep waters, (3) steep slopes (Copping et al. 2018). These characteristics facilitate feeding and thermoregulation: Steep slopes are thought to enhance upwelling which results in higher primary productivity. While whale sharks feed in deeper waters on abundant prey, the adjacent shallow, warm waters allow for thermoregulation after dives to cooler waters (Copping et al. 2018).

Overall, aggregations and movements are associated with high prey availability caused by dynamic oceanic conditions (Heyman et al. 2001, Meekan et al. 2006, Norman et al. 2017). In Belize for example, whale sharks seasonally aggregate during fish spawning events (Heyman et al. 2001). Additionally, tagged animals from Ningaloo Reef in Australia showed migratory movements into productive areas in the Indian Ocean known for tuna spawning (Heyman et al. 2001). Fatty acid analysis suggest that whale sharks can exhibit high variability in in foraging ecology, such as prey items consumed and feeding locations (Marcus et al. 2016). This suggests that high variability in movement patterns could be partially attributed to variability in feeding ecology.

At the majority of aggregation sites juvenile males dominate, while the whereabouts and migratory pathways of mature whale sharks remain largely unknown (Acuña-Marrero et al. 2014, Norman et al. 2017) suggesting that such aggregations are not for reproductive purposes. Although large, possibly pregnant females have been observed in the Galapagos Islands (Hueter et al. 2013, Hearn et al. 2016). speculated that female movement might be related to thermoregulation, as thermal preferences might differ between males and females due to differential energetic requirements and pupping (Hueter et al. 2013). Such hypothesis remain to be tested as accounts of pregnant females and newborn whale sharks are extremely rare, for example (Norman et al. 2017). Additionally, Norman et al. 2017 suggest that because coastal aggregations are predominantly segregated by age and size, the need to find suitable mates could be a major driver for large-scale migration (Norman et al. 2017).

Overall, the association of whale shark aggregations with bathymetric features resulting in enhanced feeding and thermoregulation have been well researched. However, for a species that is studied in most known aggregation sites around the world, key questions such as why aggregations consist mainly of juvenile males and the movement ecology of adults remain poorly understood.

**Order: Myliobatiformes**

**Cownose ray** (*Rhinoptera bonasus*):

Cownose rays undergo large-scale migrations along the US East Coast (Omori and Fisher 2017, Ogburn et al. 2018). Animals tagged in Chesapeake Bay spend the summer in the estuary which acts as a feeding ground and nursery area (Omori and Fisher 2017, Ogburn et al. 2018). However, mature males also migrate north to a secondary feeding ground. Reasons for this are unclear, it’s been speculated to relate to sex-differences in feeding ecology or energetic requirements (Omori and Fisher 2017) In autumn, both sexes migrate south and overlap spatially at overwintering sites in Florida (Omori and Fisher 2017), before returning to summer feeding grounds (Ogburn et al. 2018).

Movements of individuals from the Gulf of Mexico and Western Florida are thought to differ from conspecifics on the East Coast of the USA (Collins et al. 2008). Here, animals remain within Florida estuaries even when winter temperatures drop showing that this species can tolerate lower temperatures. Therefore, temperature as a direct driver for migration may depend on other trade-offs such as food availability (Collins et al. 2008). Overall, temperature, reproduction and food availability have been suggested to drive movements in this species.

**Reef manta ray** (*Manta alfredi*):

While reef manta rays were previously thought to be a predominantly resident species, satellite tagging studies show they undergo large-scale movements in some regions (Jaine et al. 2014, Armstrong et al. 2020). Migrations and local abundance along the East Coast of Australia are believed to be associated with seasonal variation in productivity and temperature (Couturier et al. 2011, Jaine et al. 2014). For example, high localized chlorophyll-a levels around Lady Elliot Island are caused by upwelling through the mesoscale, cyclonic Capricorn Eddy. This appears to attract mantas at certain times of the year (Jaine et al. 2014). Furthermore, seasonally strong tides facilitate nutrient influx in the region (Jaine et al. 2014). Contrary to this, tagging studies in the Red Sea show comparatively localized movements (Braun et al. 2015). Indeed, seasonal conditions in the Red Sea are more stable, and year-round upwelling occurs in regions frequented by mantas (Braun et al. 2015). Varying dynamic oceanic conditions in different geographical regions may produce distinct movement patterns among populations.

Information on reef manta reproduction and movement is sparse. Immature individuals appear to share the same habitat as the general population (Kessel et al. 2017) but smaller juveniles and young-of-the-year (YOY) are rarely observed. However, Marshall and Bennett 2010 observed potential YOY near an aggregation site in Mozambique. There, pregnant females were observed to leave the site, only to return a few days later, seemingly non-pregnant (Marshall and Bennett 2010), therefore undergoing small-scale movements to birthing grounds. In Sudan, however, adults, juveniles and newborns overlap spatially, suggesting that migration specifically for parturition is not occurring in females (Kessel et al. 2017).

Overall, seasonal oceanographic conditions resulting in food availability appear to drive reef manta movements, however how reproduction and temperature affect reef manta ray movement remains poorly understood.

**Extended Table 1 from main text:**

| Species | Life history requirements associated with movement | Information | Source of information | Sources |
| --- | --- | --- | --- | --- |
| Basking shark (*Cetorhinus maximus)* | Feeding, reproduction, temperature | Show directed movement towards high-density prey patches and along thermal fronts. Latitudinal movements across seasons and dive to deeper, colder waters when at lower latitudes. Aggregation sites in productive locations with similar topography. Although hypothesized, no evidence exists yet for reproduction or temperature as direct drivers | Satellite telemetry (horizontal and vertical movements), satellite imagery (productivity), prey availability modelling, remote sensing sensing (ocean fronts), stomach content analysis | Sims et al. 2000, Sims et al. 2006, Gore et al. 2008, Skomal et al. 2009, Doherty et al. 2017, Crowe et al. 2018 |
| Blacktip shark (*Carcharhinus limbatus*) | Feeding, reproduction,  temperature | Females may migrate to coastal areas to give birth, mass movements of adults associated with bait fish spawning but also correlated with temperature. In South Africa, movements are associated with sardine runs | Seasonal abundance sampling (nets, aerial), known nursery areas, tracking prey and juvenile shark overlap, inferring prey overlap through previous studies on prey biology, temperature measurements, stomach content analysis | Simpfendorfer and Milward 1993, Heupel and Hueter 2002, Hoffmayer and Parsons 2003, Kajiura and Tellman 2016 |
| Blue shark (*Prionace glauca*) | Feeding, reproduction, temperature | Show association with highly productive oceanic systems. Depth overlap with prey species at aggregation sites. Move to lower latitudes and are associated with warm currents in Winter. Presumed female movement to parturition sites | Satellite telemetry (horizontal and vertical movements), temperature measurements, productivity measurements, inferring prey horizontal and vertical spatial overlap from other studies, data on reproductive status and location of nursery areas, stable isotope and stomach content analysis | MacNeill et al. 2005, da Silva et al. 2010, Stevens et al. 2010, Williams et al. 2010, Campana et al. 2011, Vandeperre et al. 2014, Hernandez-Aguilar et al. 2016, Vandeperre et al. 2016, Howey et al. 2017, Maxwell et al. 2019 |
| Broadnose sevengill shark (Notorynchus cepedianus) | Feeding, reproduction | Move into coastal areas in Tasmania following prey. Absence of smaller size-classes and little mating behaviour rules out reproduction as driver for movement into Tasmania. When leaving coastal areas in Winter show sex-specific migration with males undergoing large-scale movements up the New South Wales coast while some females remain in coastal Tasmania. In the USA, movement into estuary systems coincides with high food availability. A large female in Pacific North-West showed movement to potential birthing and/or nursery areas. | Satellite telemetry (horizontal and vertical movements), acoustic telemetry, temperature measurements, stable isotope analysis, stomach content analysis, reproductive studies (hormone analysis + lack of mating scars), prey tracking and investigation of spatial overlap with sharks, energetics analysis, catch rate surveys, potential nursery areas identified | Ebert 1996, Barnett et al. 2010a,b,c,d, Abrantes and Barnett 2011, Barnett et al. 2011, Williams et al. 2011, Barnett and Semmens 2012, Williams et al. 2012, Awruch et al. 2014, Stehfest et al. 2014 |
| Bull shark (*Carcharhinus leucas*) | Feeding, reproduction, temperature | Show round-trip migrations to and from the central Great Barrier Reef, coinciding with suspected high prey availability. Seasonal presence at low latitude sites such as Sydney Harbour, associated with food availability. Female bull sharks likely migrate into river systems to give birth. Limited data suggests female natal philopatry. Movement broadly associated with temperature changes in South Africa and Australia | Satellite telemetry (horizontal and vertical movements), acoustic telemetry, genetics, inferred prey life history from other studies, tracking prey and shark overlap, temperature measurements, identified nursery areas, stable isotope analysis, stomach content analysis, catch rates | Cliff and Dudley 1991, Carlson et al. 2010, Matich et al. 2011, Werry et al. 2011, Tillett et al. 2012, Brunnschweiler and Barnett 2013, Daly et al. 2014, Heupel et al. 2015, Lea et al. 2015, Espinoza et al. 2016, Glaus et al. 2019, Lee et al. 2019, Smoothey et al. 2019 Niella et al. 2020 |
| Cownose ray (*Rhinoptera bonasus*) | Feeding,reproduction, temperature | In summer reside in temperate pupping and mating areas, overwinter further south.  Feeding and pupping observed during seasonal presence in coastal bays and estuaries. Ray abundance has been linked to productive areas. | Satellite telemetry (horizontal and vertical movements), acoustic telemetry, identified nursery areas, aerial surveys, gill net sampling, temperature measurements, stomach content analysis | Collins et al. 2007a,b, Collins et al. 2008, Goodman et al. 2011, Omori and Fisher 2017, Ogburn et al. 2018 |
| Dusky shark (*Carcharhinus obscurus*) | Reproduction, feeding, temperature | Large, near-term pregnant females thought to migrate from offshore areas to coastal parturition sites. Partial migration to pupping grounds observed in Western Australia.  Movement of sharks in concordance with squid spawning and sardine runs.  Colder Winter temperatures in some areas might drive migration | Satellite telemetry (horizontal and vertical movements), acoustic telemetry, capture/re-capture studies, temperature measurements, nursery area identified, spatial overlap with prey and prey life history inferred from other studies, stable isotope and stomach content analysis | Gelsleichter et al. 1999, Hussey et al. 2009, Hussey et al. 2011, Rogers et al. 2013, Hoffmayer et al. 2014, Braccini et al. 2018 |
| Great hammerhead (*Sphyrna mokarran*) | Reproduction, feeding, temperature | Females are believed to migrate to pupping areas. Large-scale migration roughly coinciding with potential prey movement (e.g. dolphin fish, smaller elasmobranchs etc.). May move with seasonal temperature changes | Satellite telemetry, acoustic telemetry, prey tracks and prey life history traits inferred from other studies, temperature measurements, potential nursery areas identified, stomach content analysis | Stevens and Lyle 1989, Hammerschlag et al. 2011, Barker et al. 2017, Guttridge et al. 2017, Raoult et al. 2019 |
| Grey nurse shark (*Carcharias Taurus)* | Feeding, reproduction, temperature | Pregnant females aggregate during gestation, then move to pupping grounds. Males seem to migrate to aggregation sites to mate, then move further offshore. Movement patterns of males and non-pregnant females similar. In Atlantic, females do not seem to move for parturition, juvenile movement to areas of high prey density, offshore movements of some females and males potentially for feeding? | Satellite telemetry (horizontal and vertical movements), acoustic telemetry, visual behavioural observations (photo-ID), stomach content analysis, temperature measurements, potential gestation areas identified, potential nursery areas identified | Gelsleichter et al. 1999, Lucifora et al. 2002, Smale 2005, Bansemer and Bennett 2011, Kneebone et al. 2012, Kneebone et al. 2014, Teter et al. 2015 |
| Porbeagle shark (*Lamna nasus*) | Feeding, reproduction | Migration along shelf break areas broadly overlaps with prey (mackerel and herring). Movements into Gulf of St. Lawrence could be related to eel migration. Mackerel disperse into deeper waters after spawning, broadly overlapping with movements of porbeagle sharks. Cyclic migration in north-east Atlantic overlaps with different prey items across season. North-South migrations for parturition in Southern hemisphere. Mature females in North-Atlantic migrate to potential subtropical pupping ground. Pregnant females and small juveniles overlap in Bay of Biscay and Celtic Sea (possible natal philopatry). Cyclic migration in North-East Atlantic, with some northward movements in Autumn-Winter, suggesting low temperature not a major driver (possible upper limit of about 21.9C). | Satellite telemetry (horizontal and vertical movements), temperature measurements, oceanographic conditions inferred from other studies, prey overlap inferred from other studies, reproductive data on females in relation to migration, stomach content analysis | Gauld 1989, Joyce et al. 2002, Steven and Joyce 2003, Pade et al. 2009, Campana et al. 2010, Saunders et al. 2011, Béguer-Pon et al. 2012, Francis et al. 2015, Biais et al. 2017, Cameron et al. 2018 |
| Reef manta *(Mobula alfredi)* | Feeding, reproduction? Temperature? | Movements in Eastern Australia are associated with food availability caused by seasonal, dynamic oceanic conditions. Directional large scale-movements evident in Western Australia. Movement in Maldives influenced by monsoons as they impact productivity. Fatty acid analysis shows diet is based on demersal zooplankton. Movement may be linked to tracking this prey type. Reproduction in relation to movement not well known. Small juveniles and young-of-the-year are rarely observed. Pregnant females at aggregation sites, leave and re-appear a few days later, seemingly non-pregnant suggesting at least small scale movements to birthing grounds. In other areas, adults, juveniles and newborns overlap spatially, migration for parturition questionable. Temperature effects evident in Australia | Satellite telemetry (horizontal and vertical movements), acoustic telemetry, abundance counts and behavioural observations (photo-ID), oceanographic conditions inferred from other studies, temperature measurements, remote sensing of environmental factors, fatty acid analysis | Marshall and Bennett 2010, Anderson et al. 2011, Jaine et al. 2012, Couturier et al. 2013, Jaine et al. 2014, Braun et al. 2015, Kessel et al. 2017, Stewart et al. 2018, Peel et al. 2019, Armstrong et al. 2020a,b |
| Salmon shark (*Lamna ditropis*) | Feeding, reproduction | Individuals moving to productive areas show more area-restricted-search behaviour, movement occurs between productive regions. Higher overlap with salmon and herring in Northern areas. Movement of females and observation of young-of-the year sharks suggest migration to nursery grounds. May track temporally separated salmon runs across Pacific North-West. No apparent low-temperature effect, as some individuals remain at high latitudes over Winter. Emigration from Alaskan coastal areas in winter more likely due to low prey densities, competition and reproduction | Satellite telemetry (horizontal and vertical movements), remote sensing of productivity levels, temperature measurements, physiological studies, mating scars observed, areas with young-of-the year identified | Hulbert et al. 2005, Weng et al. 2005, Weng et al. 2008, Williams et al. 2010 |
| Scalloped hammerhead (*Sphyrna lewini*) | Feeding, reproduction, temperature | In Tropical Pacific aggregations at archipelagos and sea mounts linked to food availability and ocean productivity. Long-distance movements of pregnant females to coastal nursery areas proposed, as juveniles are found in high abundance in shallow coastal areas. In South Africa decline in catch rates as temperatures drop. | Satellite telemetry (horizontal and vertical movements), acoustic telemetry, temperature measurements, remote sensing for productivity levels, potential nursery areas identified, catch rates in control programs, analysis of habitat structure, stable isotope and stomach content analysis | Stevens and Lyle 1989, Duncan and Holland 2006, Bessudo et al. 2011, Diemer et al. 2011, Bornatowski et al. 2014, Ketchum et al. 2014, Loor‐Andrade et al. 2015, Wells et al. 2018, Nalesso et al. 2019 |
| School shark (*Galeorhinus galeus*) | Reproduction, feeding, environmental factors (temperature, salinity) | Show broad correlation in large-scale movement with prey movements. Female partial migration to pupping grounds, some females remain resident and give birth without migrating (philopatry?). Aggregations in warmer waters might relate to growth and embryonic development. Some movements superficially, associated with temperature and salinity | Satellite tagging (horizontal and vertical movements), mark-recapture surveys, temperature measurements, potential pupping grounds identified, salinity measurements, ocean productivity measurements, overlap of prey animals inferred from other studies, stomach content analysis | Lucifora et al. 2006, McAllister et al. 2015, Jaureguizar et al. 2018, McAllister et al. 2018, McMillan, et al. 2018, McMillan et al. 2019, Thorburn et al. 2019 |
| Short-fin mako shark (*Isurus oxyrinchus*) | Feeding, reproduction, temperature? | Globally, movement patterns are associated with patterns of resource distribution and oceanographic conditions. In Eastern Pacific, movement corresponds to seasonal increase in productivity, upwelling and sardine abundance. Higher numbers of juvenile mako sharks are present in some areas, suggesting a female migration to pupping grounds. Globally, North-South movements depending on season evident, but high variability might indicate differences in food availability rather than temperature drives movement. | Satellite telemetry (horizontal and vertical movements), remote sensing for temperature and productivity levels, prey life history and spatial overlap inferred from other studies, potential nursery areas identified, modelling of habitat characteristics, stable isotope and stomach content analysis | MacNeill et al. 2005, Rogers et al. 2015, Vaudo et al. 2017, Byrne et al. 2019, Francis et al. 2019, Nasby-Lucas et al. 2019 |
| Tiger shark (*Galeocerdo cuvier*) | Feeding, reproduction, temperature (exploit thermal optimum) | Globally, movements are associated with prey aggregations, such as albatross and sea turtles and areas of high productivity. Female movement to gestation sites and nursery areas proposed. Genetic studies suggest natal philopatry in females. Males may migrate to increase mate encounter rates. Temperature appears to have an effect on movement in some areas, especially for animals at range limits. | Satellite telemetry (horizontal and vertical movements), acoustic telemetry, stable isotope analysis, analysis of spatio-temporal overlap with prey species, prey life history and movement tracks inferred from other studies, temperature measurements, oceanographic conditions measured, analysis of activity patterns and temperature-correlation, catch rates, productivity level measurements, hormone level analysis for reproduction, ultrasounds, general preliminary hormone analysis, stomach-content analysis, “critter-cams”, genetics, mating scars observed, potential gestation areas identified, potential nursery areas identified. | Lowe et al. 1996, Heithaus 2001, Heithaus et al. 2007, Whitney and Crow 2007, Meyer et al. 2010, Fitzpatrick et al. 2012, Hammerschlag et al. 2012, Heithaus et al. 2012, Papastamatiou et al. 2013, Holmes et al. 2014, Werry et al. 2014, Ferreira et al. 2015, Hammerschlag et al. 2015, Lea et al. 2015, Bernard et al. 2016, Hammerschlag et al. 2016, Sulikowski et al. 2016, Acuna-Marrero et al. 2017, Dicken et al. 2017, Ferreira et al. 2017, Daly et al. 2018, Lea et al. 2018, Meyer et al. 2018, Payne et al. 2018, Wosnick et al. 2018, Pirog et al. 2019, Ajemian et al. 2020, Lipscombe et al. 2020 |
| White shark (*Carcharodon carcharias*) | Reproduction, feeding, potentially temperature | Sharks aggregate in an oceanic area near Hawaii, presumably to mate. No concentrated food source around aggregation site, sharks return to coastal waters seemingly emaciated. Stable isotope analysis reveals reduced feeding in offshore (at least in males, female data lacking). Movements have been associated with prey aggregations and general productivity in other areas. Seasonal upwelling could directly affect white sharks. Show seasonal association with eddies in Atlantic and Pacific. Movement of females to coastal pupping sites. | Satellite telemetry (horizontal and vertical movements), acoustic telemetry, temperature measurements, prey life history and spatial overlap with prey inferred from other studies, visual behavioural observations, potential nursery areas identified, , oceanographic and environmental data, stable isotope and stomach content analysis, genetics | Bonfil et al. 2005, Bruce et al. 2006, Weng et al. 2007, Domeier and Nasby-Lucas 2008, Nasby-Lucas et al. 2009, Jorgensen et al. 2010, Carlisle et al. 2012, Hussey et al. 2012, Jorgensen et al. 2012, Domeier and Nasby-Lucas 2013, Malpica‐Cruz et al. 2013, Curtis et al. 2014, Skomal et al. 2017, Gaube et al. 2018, Huveneers et al. 2018, Bradford et al. 2020, Spaet et al. 2020a,b |
| Whale shark (*Rhincodon typus*) | Feeding, potentially reproduction, temperature (thermoregulation) | Whale shark aggregations and movements often associated with high prey density and plankton blooms, which is likely associated with topography. Migration to pupping areas and for thermoregulation speculated | Satellite telemetry (Horizontal and vertical movements), stable isotope/fatty acid analysis, temperature measurements, ocean productivity, photo ID, plane surveys, prey life history and spatial overlap inferred from other studies, habitat structure analysis, stomach content analysis | Heyman et al. 2001, Wilson et al. 2006, Hsu et al. 2007, Ramírez-Macías, et al. 2012, Hueter et al. 2013, Rohner et al. 2013, Acuña-Marrero et al. 2014, Hearn et al. 2016, Marcus et al. 2016, Norman et al. 2017, Copping et al. 2018 |

**Extended Table 2 from main text:**

| Species | Differences in movement behaviour within regions? | Differences in movement behaviour between regions? | Does the species aggregate naturally (no artificial feeding)? | Evidence of context-dependence | Source |
| --- | --- | --- | --- | --- | --- |
| Basking shark (*Cetorhinus maximus*) | Yes | Yes | Yes | Dynamic oceanographic settings vary between West and East Atlantic, potentially resulting in movement differences. Migrate latitudinal and longitudinal. Show high individual variability | Skomal et al. 2009, Doherty et al. 2017, Braun et al. 2018 |
| Blacktip shark (*Carcharhinus limbatus*) | Yes | NA | Yes | In South Africa, females aggregate and show high site fidelity, interspersed with movements. Males are highly mobile and associated with sardine runs | Daly (unpublished data) |
| Blue shark (*Prionace glauca*), | Yes | Yes | Yes | Movement differs between ocean basins, studies on billfish and tuna suggest oceanographic processes resulting in shallow thermoclines with hypoxic areas compress habitat in the tropical Pacific compared to the Atlantic. This may play a role in shaping movement of blue sharks as basically no crossing of tropical latitudes is evident in the Pacific compared to Atlantic. Not all demographics migrate to aggregation sites, at aggregation sites sexes use different temperature niches hinting at sex-specific requirements. Complex movement patterns: Sex and size differences and regional differences | Kohler et al. 1998, Prince and Goodyear 2006, da Silva et al. 2010, Block et al. 2011, Vandeperre et al. 2014, King et al. 2015, Howey et al. 2017, Vedor et al. 2021 |
| Broadnose sevengill shark (Notorynchus cepedianus) | Yes | Yes | Yes | Partial migration is evident in Tasmania and reproductive strategies of sexes (individual context) might shape movement decisions within the population. Sequential tracking of sharks and prey suggests migration due to prey concentrations with individual site fidelity evident. Reproductive studies rule out breeding as driver into coastal Tasmania. However, movement to suspected nursery areas in USA may take place. Differential movement and resource separation by individuals occurs in Tasmania. Females make larger movements in USA, while males do in Australia. | Barnett et al. 2010, Abrantes and Barnett 2011, Barnett et al. 2011, Williams et al. 2011, Williams et al. 2012, Stehfest et al. 2014 |
| Bull shark (*Carcharhinus leucas*), | Yes | Yes | Yes | Movement in Australia and Southern Africa potentially driven by seasonal changes in temperature and food availability. Movements in South-Eastern United States more limited, potentially due to more stable conditions. Bull sharks exhibit high variability in movement patterns within regions, with some individuals moving large distances while others remain more localised. Animals at range limits appear to migrate differentially to animals in centre. Skipped-breeding migration and natal philopatry to river systems could result in variability among females. | Tillett et al. 2012, Daly et al. 2013, Daly et al. 2014, Heupel et al. 2015, Lea et al. 2015, Espinoza et al. 2016, Lee et al. 2019 |
| Cownose ray (*Rhinoptera bonasus*) | Yes | Yes | Yes | Individual philopatry to different summering areas suggests individual context shapes movement strategies, males have secondary feeding grounds in Atlantic, females do not. Stock in Gulf of Mexico differs in movement to Atlantic | Collins et al. 2008, Omori and Fisher 2017, Ogburn et al. 2018 |
| Dusky shark *(Charcharhinus obscurus*) | Yes | Yes | Yes | Partial migration observed in Western Australia for females suggests individual context (reproductive status) shapes movements to nurseries. Differences in movement between size classes. | Hussey et al. 2011, Braccini et al. 2018 |
| Great hammerhead (*Sphyrna mokarran*) | Yes | NA | NA | Partial migration in the South-West Atlantic could be influenced by reproductive status of females migrating to parturition sites (individual context) | (Guttridge et al., 2017) |
| Grey nurse shark (*Carcharias Taurus)* | Yes | Yes | Yes | Size class differences in movement within regions, differences in reproductive movements between regions. Juveniles move further in Atlantic, while moving less in Australia and South Africa. Females in Australia and South Africa appear to have three distinct habitats, one each for mating, gestation and parturition. Mating occurs in colder waters; females then move to warmer waters for gestation to return again to slightly cooler waters for parturition. However, females in North and South America remain in low-latitude, warm waters for gestation and parturition. Females of narrow size range make offshore instead of north-south movements in Eastern USA | Gelsleichter et al. 1999, Lucifora et al. 2002, Smale 2005, Bansemer and Bennett 2011, Smale et al. 2012, Kneebone et al. 2014, Teter et al. 2015 |
| Porbeagle shark (*Lamna nasus*) | Yes | Yes | Yes | Show individual differences in migration routes in North-Atlantic. Movement of a tagged males differs to female movement (sex-differences) in North-East Atlantic. One individual found to undergo transatlantic longitudinal migration. In North-West Atlantic males and immature animals remained off shelf in temperate areas while females moved to subtropical areas potentially to give birth in Sargasso Sea. Females and juveniles overlap spatially in high latitude areas in north-east Atlantic, no apparent movement for parturition. Individual variability in movements in North-East Atlantic when tracking prey | Pade et al. 2009, Campana et al. 2010, Biais et al. 2017, Cameron et al. 2018 |
| Reef manta *(Mobula alfredi)* | Yes | Yes | Yes | Rays encountering dynamic oceanographic conditions in Australia resulting in seasonal upwelling show large-scale movements, while rays in the Red Sea show limited movements suggesting stable oceanographic conditions result in relatively favourable conditions year-round. In some regions females may migrate to birthing grounds while in other areas adults and neonates overlap spatially | Marshall and Bennett 2010, Jaine et al. 2014, Braun et al. 2015, Kessel et al. 2017, |
| Salmon shark (*Lamna nasus*) | Yes | NA | Yes | Some animals remain in high latitudes during Winter, suggesting internal state (such as cold tolerance) could determine residency/migration. Competition may play a role. Reproductive status may shape individual female movements to different nursery areas. Potentially two different movement strategies for parturition in Eastern Pacific. | Hulbert et al. 2005, Wenig et al. 2008 |
| Scalloped hammerhead (*Sphyrna lewini*) | Yes | Yes | Yes | Partial migration is evident in the Eastern Pacific and reproductive status of females (individual context) may shape movement decisions. Animals tagged at different island groups differ in movement. Sexual segregation in space use in Gulf of Mexico | Bessudo et al. 2011, Ketchum et al. 2014, Wells et al. 2018, Nalesso et al. 2019 |
| School shark (*Galeorhinus galeus*) | Yes | NA | Yes | Partial migration evident in Atlantic and Pacific and reproductive status of females (individual context) shapes movement decisions within the population. Some females migrate to pupping Areas in Southern Australia, while others give birth locally – philopatry? | McMillan et al. 2019, Thorburn et al. 2019 |
| Short-fin mako shark (*Isurus oxyrinchus*) | Yes | Yes | NA | Different populations show resident vs. transient behaviours based on seasonal consistency of primary production. Differences in magnitude of environmental change shapes different movement patterns in different regions. | Vaudo et al. 2017, Byrne et al. 2019, Francis et al. 2019 |
| Tiger shark (*Galeocerdo cuvier*) | Yes | Yes | Yes | Movements are more restricted around oceanic islands with narrow shelves compared to movements in areas with more extensive shelves, such as along continents. Movement patterns appear more restricted in Southern Africa and Eastern Australia compared to Atlantic and Western Australia.  Partial migrations evident in most populations.  Movements in Hawaii differ between islands, depending on resource distribution around islands. Movement also differs based on sex, suggesting reproductive strategies and breeding cycles shape movement. Size classes differ in movement behaviour and in their response to seasonal environmental factors. Stable isotope values differ between regions in Australia and so do extent of movements, suggesting a link between feeding behaviour and movement strategy. Animals at range limits appear to migrate differently to animals in center of distribution. Despite being thermal generalist, in some regions respond differently to temperature. High individual differences within regions create variability in movement. Latitudinal vs. longitudinal movements in different regions of Australia. | Heithaus et al. 2007, Fitzpatrick et al. 2012, Hammerschlag et al. 2012, Papastamatiou et al. 2013, Holmes et al. 2014, Werry et al. 2014, Ferreira et al. 2015, Lea et al. 2015, Acuna-Marrero et al. 2017, Ferreira et al. 2017, Daly et al. 2018, Lea et al. 2018, Meyer et al. 2018, Ajemian et al. 2020, Lipscombe et al. 2020 |
| White shark (*Carcharodon carcharias*) | Yes | Yes | Yes | Offshore movements occur in some populations but patterns of offshore movements less clear in Atlantic while more synchronized in the Eastern Pacific. Sex-and size-specific migrations in Eastern Pacific and Southern Australia. Latitudinal vs. longitudinal movements in different regions. Females may differ in movement patterns based on reproductive cycle. Seasonal North-South movements in Atlantic staggered between individuals, suggesting individual context may play a role. Environmental preferences appear to differ between Atlantic and Pacific. | Domeier and Nasby-Lucas 2013, Skomal et al. 2017, Gaube et al. 2018, Bradford et al. 2020 |
| Whale shark (*Rhincodon typus*) | Yes | Yes | Yes | Environmental conditions at aggregation sites across ocean basins concentrate sharks, suggesting that globally animals use similar environmental contexts on a seasonal basis. After aggregating around the Yucatan Peninsula, whale sharks disperse into different directions. One female migrate across the equator into South Atlantic. Possible feeding specialization suggests variability in feeding ecology could shape variability in movement ecology. Generally, sexes and size classes seperated | Hueter et al. 2013, Marcus et al. 2016, Copping et al. 2018 |

**References**

Abrantes, K. G., & Barnett, A. (2011). Intrapopulation variations in diet and habitat use in a marine apex predator, the broadnose sevengill shark Notorynchus cepedianus. *Marine Ecology Progress Series, 442*, 133-148.

Acuña-Marrero, D., Jiménez, J., Smith, F., Doherty Jr, P. F., Hearn, A., Green, J. R., . . . Salinas-de-León, P. (2014). Whale shark (Rhincodon typus) seasonal presence, residence time and habitat use at Darwin Island, Galapagos Marine Reserve. *PloS one, 9*(12).

Ajemian, M. J., Drymon, J. M., Hammerschlag, N., Wells, R. D., Street, G., Falterman, B., . . . Fischer, C. (2020). Movement patterns and habitat use of tiger sharks (Galeocerdo cuvier) across ontogeny in the Gulf of Mexico. *PloS one, 15*(7), e0234868.

Armstrong, A. J., Armstrong, A. O., Bennett, M. B., McGregor, F., Abrantes, K. G., Barnett, A., . . . Dudgeon, C. L. (2020a). The geographic distribution of reef and oceanic manta rays (Mobula alfredi and Mobula birostris) in Australian coastal waters. *Journal of Fish Biology, 96*(3), 835-840.

Armstrong, A. J., Armstrong, A. O., McGregor, F., Richardson, A. J., Bennett, M. B., Townsend, K. A., . . . Dudgeon, C. L. (2020b). Satellite tagging and photographic identification reveal connectivity between two UNESCO World Heritage Areas for reef manta rays. *Frontiers in Marine Science, 7*, 725.

Awruch, C. A., Jones, S. M., Asorey, M. G., & Barnett, A. (2014). Non-lethal assessment of the reproductive status of broadnose sevengill sharks (Notorynchus cepedianus) to determine the significance of habitat use in coastal areas. *Conservation physiology, 2*(1), cou013.

Bansemer, C., & Bennett, M. (2011). Sex-and maturity-based differences in movement and migration patterns of grey nurse shark, Carcharias taurus, along the eastern coast of Australia. *Marine and Freshwater Research, 62*(6), 596-606.

Barker, A. M., Frazier, B. S., Bethea, D. M., Gold, J. R., & Portnoy, D. S. (2017). Identification of young-of-the-year great hammerhead shark Sphyrna mokarran in northern Florida and South Carolina. *Journal of Fish Biology, 91*(2), 664-668. doi:10.1111/jfb.13356

Barnett, A., Abrantes, K., Stevens, J. D., Yick, J. L., Frusher, S. D., & Semmens, J. M. (2010a). Predator-prey relationships and foraging ecology of a marine apex predator with a wide temperate distribution. *Marine Ecology Progress Series, 416*, 189-200. doi:10.3354/meps08778

Barnett, A., Abrantes, K. G., Stevens, J. D., Bruce, B. D., & Semmens, J. M. (2010b). Fine-Scale Movements of the Broadnose Sevengill Shark and Its Main Prey, the Gummy Shark. *PloS one, 5*(12). doi:10.1371/journal.pone.0015464

Barnett, A., Abrantes, K. G., Stevens, J. D., & Semmens, J. M. (2011). Site fidelity and sex-specific migration in a mobile apex predator: implications for conservation and ecosystem dynamics. *Animal Behaviour, 81*(5), 1039-1048. doi:10.1016/j.anbehav.2011.02.011

Barnett, A., Redd, K. S., Frusher, S. D., Stevens, J. D., & Semmens, J. M. (2010c). Non-lethal method to obtain stomach samples from a large marine predator and the use of DNA analysis to improve dietary information. *Journal of experimental marine biology and ecology, 393*(1-2), 188-192. doi:10.1016/j.jembe.2010.07.0

Barnett, A., & Semmens, J. M. (2012). Sequential movement into coastal habitats and high spatial overlap of predator and prey suggest high predation pressure in protected areas. *Oikos, 121*(6), 882-890. doi:10.1111/j.1600-0706.2011.20000.x

Barnett, A., Stevens, J. D., Frusher, S. D., & Semmens, J. M. (2010d). Seasonal occurrence and population structure of the broadnose sevengill shark Notorynchus cepedianus in coastal habitats of south-east Tasmania. *Journal of Fish Biology, 77*(7), 1688-1701. doi:10.1111/j.1095-8649.2010.02810.x

Béguer-Pon, M., Benchetrit, J., Castonguay, M., Aarestrup, K., Campana, S. E., Stokesbury, M. J., & Dodson, J. J. (2012). Shark predation on migrating adult American eels (Anguilla rostrata) in the Gulf of St. Lawrence. *PloS one, 7*(10), e46830.

Bernard, A. M., Feldheim, K. A., Heithaus, M. R., Wintner, S. P., Wetherbee, B. M., & Shivji, M. S. (2016). Global population genetic dynamics of a highly migratory, apex predator shark. *Molecular Ecology, 25*(21), 5312-5329. doi:10.1111/mec.13845

Bessudo, S., Soler, G. A., Klimley, A. P., Ketchum, J. T., Hearn, A., & Arauz, R. (2011). Residency of the scalloped hammerhead shark (Sphyrna lewini) at Malpelo Island and evidence of migration to other islands in the Eastern Tropical Pacific. *Environmental Biology of Fishes, 91*(2), 165-176.

Biais, G., Coupeau, Y., Séret, B., Calmettes, B., Lopez, R., Hetherington, S., & Righton, D. (2017). Return migration patterns of porbeagle shark (Lamna nasus) in the Northeast Atlantic: implications for stock range and structure. *ICES Journal of Marine Science, 74*(5), 1268-1276.

Block, B. A., Jonsen, I. D., Jorgensen, S. J., Winship, A. J., Shaffer, S. A., Bograd, S. J., . . . Costa, D. P. (2011). Tracking apex marine predator movements in a dynamic ocean. *Nature, 475*(7354), 86-90. doi:10.1038/nature10082

Bonfil, R., Meyer, M., Scholl, M. C., Johnson, R., O'Brien, S., Oosthuizen, H., . . . Paterson, M. (2005). Transoceanic migration, spatial dynamics, and population linkages of white sharks. *Science, 310*(5745), 100-103. doi:10.1126/science.1114898

Bornatowski, H., Braga, R., Abilhoa, V., & Corrêa, M. (2014). Feeding ecology and trophic comparisons of six shark species in a coastal ecosystem off southern Brazil. *Journal of Fish Biology, 85*(2), 246-263.

Braccini, M., de Lestang, S., & McAuley, R. (2018). Dusky sharks (Carcharhinus obscurus) undertake large-scale migrations between tropical and temperate ecosystems. *Canadian Journal of Fisheries and Aquatic Sciences, 75*(9), 1525-1533. doi:10.1139/cjfas-2017-0313

Bradford, R., Patterson, T. A., Rogers, P. J., McAuley, R., Mountford, S., Huveneers, C., . . . Bruce, B. D. (2020). Evidence of diverse movement strategies and habitat use by white sharks, Carcharodon carcharias, off southern Australia. *Marine biology, 167*(7). doi:ARTN 9610.1007/s00227-020-03712-y

Braun, C. D., Skomal, G. B., & Thorrold, S. R. (2018). Integrating archival tag data and a high-resolution oceanographic model to estimate basking shark (Cetorhinus maximus) movements in the Western Atlantic. *Frontiers in Marine Science, 5*, 25.

Braun, C. D., Skomal, G. B., Thorrold, S. R., & Berumen, M. L. (2015). Movements of the reef manta ray (Manta alfredi) in the Red Sea using satellite and acoustic telemetry. *Marine biology, 162*(12), 2351-2362.

Bruce, B., Stevens, J., & Malcolm, H. (2006). Movements and swimming behaviour of white sharks (Carcharodon carcharias) in Australian waters. *Marine biology, 150*(2), 161-172.

Brunnschweiler, J. M., & Barnett, A. (2013). Opportunistic Visitors: Long-Term Behavioural Response of Bull Sharks to Food Provisioning in Fiji. *PloS one, 8*(3). doi:ARTN e5852210.1371/journal.pone.0058522

Byrne, M. E., Vaudo, J. J., Harvey, G. C. M., Johnston, M. W., Wetherbee, B. M., & Shivji, M. (2019). Behavioral response of a mobile marine predator to environmental variables differs across ecoregions. *Ecography, 42*(9), 1569-1578.

Cameron, L. W., Roche, W., Green, P., Houghton, J. D., & Mensink, P. J. (2018). Transatlantic movement in porbeagle sharks, Lamna nasus. *Fisheries Research, 207*, 25-27.

Campana, S. E., Dorey, A., Fowler, M., Joyce, W., Wang, Z. L., Wright, D., & Yashayaev, I. (2011). Migration Pathways, Behavioural Thermoregulation and Overwintering Grounds of Blue Sharks in the Northwest Atlantic. *PloS one, 6*(2). doi:ARTN e1685410.1371/journal.pone.0016854

Campana, S. E., Joyce, W., & Fowler, M. (2010). Subtropical pupping ground for a cold-water shark. *Canadian Journal of Fisheries and Aquatic Sciences, 67*(5), 769-773. doi:10.1139/F10-020

Campana, S. E., Joyce, W., & Marks, L. (2003). *Status of the porbeagle shark (Lamna nasus) population in the northwest Atlantic in the context of species at risk*: Fisheries & Oceans Canada, Science, Canadian Science Advisory Secretariat.

Carlisle, A. B., Kim, S. L., Semmens, B. X., Madigan, D. J., Jorgensen, S. J., Perle, C. R., . . . Block, B. A. (2012). Using Stable Isotope Analysis to Understand the Migration and Trophic Ecology of Northeastern Pacific White Sharks (Carcharodon carcharias). *PloS one, 7*(2). doi:ARTN e3049210.1371/journal.pone.0030492

Carlson, J. K., Ribera, M. M., Conrath, C. L., Heupel, M. R., & Burgess, G. H. (2010). Habitat use and movement patterns of bull sharks Carcharhinus leucas determined using pop-up satellite archival tags. *Journal of Fish Biology, 77*(3), 661-675. doi:10.1111/j.1095-8649.2010.02707.x

Collins, A. B., Heupel, M. R., Hueter, R. E., & Motta, P. J. (2007a). Hard prey specialists or opportunistic generalists? An examination of the diet of the cownose ray, Rhinoptera bonasus. *Marine and Freshwater Research, 58*(1), 135-144. doi:10.1071/Mf05227

Collins, A. B., Heupel, M. R., & Motta, P. J. (2007b). Residence and movement patterns of cownose rays Rhinoptera bonasus within a south-west Florida estuary. *Journal of Fish Biology, 71*(4), 1159-1178. doi:10.1111/j.1095-8649.2007.01590.x

Collins, A. B., Heupel, M. R., & Simpfendorfer, C. A. (2008). Spatial Distribution and Long-term Movement Patterns of Cownose Rays Rhinoptera bonasus Within an Estuarine River. *Estuaries and Coasts, 31*(6), 1174-1183. doi:10.1007/s12237-008-9100-5

Colloca, F., Scannella, D., Geraci, M. L., Falsone, F., Giusto, B., Vitale, S., . . . Bono, G. (2019). British sharks in Sicily: records of long-distance migration of tope shark (Galeorhinus galeus) from the north-eastern Atlantic to the Mediterranean Sea. *Mediterranean Marine Science, 20*(2), 309-313. doi:10.12681/mms.18121

Copping, J. P., Stewart, B. D., McClean, C. J., Hancock, J., & Rees, R. (2018). Does bathymetry drive coastal whale shark (Rhincodon typus) aggregations? *PeerJ, 6*, e4904.

Couturier, L. I. E., Jaine, F. R. A., Townsend, K. A., Weeks, S. J., Richardson, A. J., & Bennett, M. B. (2011). Distribution, site affinity and regional movements of the manta ray, Manta alfredi (Krefft, 1868), along the east coast of Australia. *Marine and Freshwater Research, 62*(6), 628-637. doi:10.1071/Mf10148

Crowe, L. M., O'Brien, O., Curtis, T. H., Leiter, S. M., Kenney, R. D., Duley, P., & Kraus, S. D. (2018). Characterization of large basking shark Cetorhinus maximus aggregations in the western North Atlantic Ocean. *Journal of Fish Biology, 92*(5), 1371-1384. doi:10.1111/jfb.13592

da Silva, C., Kerwath, S. E., Wilke, C. G., Meyer, M., & Lamberth, S. J. (2010). First documented southern transatlantic migration of a blue shark Prionace glauca tagged off South Africa. *African Journal of Marine Science, 32*(3), 639-642. doi:10.2989/1814232x.2010.540777

Daly, R., Smale, M. J., Cowley, P. D., & Froneman, P. W. (2014). Residency Patterns and Migration Dynamics of Adult Bull Sharks (Carcharhinus leucas) on the East Coast of Southern Africa. *PloS one, 9*(10). doi:ARTN e10935710.1371/journal.pone.0109357

Daly, R., Smale, M. J., Cowley, P. D., & Froneman, P. W. (2014). Residency Patterns and Migration Dynamics of Adult Bull Sharks (Carcharhinus leucas) on the East Coast of Southern Africa. *PloS one, 9*(10). doi:ARTN e10935710.1371/journal.pone.0109357

Daly, R., Smale, M. J., Singh, S., Anders, D., Shivji, M., K. Daly, C. A., . . . Fitzpatrick, R. (2018). Refuges and risks: Evaluating the benefits of an expanded MPA network for mobile apex predators. *Diversity and Distributions, 24*(9), 1217-1230.

Dicken, M. L., Hussey, N. E., Christiansen, H. M., Smale, M. J., Nkabi, N., Cliff, G., & Wintner, S. P. (2017). Diet and trophic ecology of the tiger shark (Galeocerdo cuvier) from South African waters. *PloS one, 12*(6).

Diemer, K., Mann, B., & Hussey, N. (2011). Distribution and movement of scalloped hammerhead Sphryna lewini and smooth hammerhead Sphyrna zygaena sharks along the east coast of southern Africa. *African Journal of Marine Science, 33*(2), 229-238.

Doherty, P. D., Baxter, J. M., Gell, F. R., Godley, B. J., Graham, R. T., Hall, G., . . . Witt, M. J. (2017). Long-term satellite tracking reveals variable seasonal migration strategies of basking sharks in the north-east Atlantic. *Scientific reports, 7*. doi:ARTN 4283710.1038/srep42837

Domeier, M. L., & Nasby-Lucas, N. (2008). Migration patterns of white sharks Carcharodon carcharias tagged at Guadalupe Island, Mexico, and identification of an eastern Pacific shared offshore foraging area. *Marine Ecology Progress Series, 370*, 221-237. doi:10.3354/meps07628

Domeier, M. L., & Nasby-Lucas, N. (2013). Two-year migration of adult female white sharks (Carcharodon carcharias) reveals widely separated nursery areas and conservation concerns. *Animal Biotelemetry, 1*(1), 1-10.

Dudgeon, C. L., Pollock, K. H., Braccini, J. M., Semmens, J. M., & Barnett, A. (2015). Integrating acoustic telemetry into mark-recapture models to improve the precision of apparent survival and abundance estimates. *Oecologia, 178*(3), 761-772. doi:10.1007/s00442-015-3280-z

Duncan, K. M., & Holland, K. N. (2006). Habitat use, growth rates and dispersal patterns of juvenile scalloped hammerhead sharks Sphyrna lewini in a nursery habitat. *Marine Ecology Progress Series, 312*, 211-221.

Espinoza, M., Heupel, M. R., Tobin, A. J., & Simpfendorfer, C. A. (2016). Evidence of Partial Migration in a Large Coastal Predator: Opportunistic Foraging and Reproduction as Key Drivers? *PloS one, 11*(2). doi:ARTN e0147608 10.1371/journal.pone.0147608

Ferreira, L. C., Thums, M., Heithaus, M. R., Barnett, A., Abrantes, K. G., Holmes, B. J., . . . Meekan, M. G. (2017). The trophic role of a large marine predator, the tiger shark Galeocerdo cuvier. *Scientific reports, 7*. doi:ARTN 764110.1038/s41598-017-07751-2

Ferreira, L. C., Thums, M., Meeuwig, J. J., Vianna, G. M., Stevens, J., McAuley, R., & Meekan, M. G. (2015). Crossing latitudes—long-distance tracking of an apex predator. *PloS one, 10*(2), e0116916.

Fitzpatrick, R., Thums, M., Bell, I., Meekan, M. G., Stevens, J. D., & Barnett, A. (2012). A comparison of the seasonal movements of tiger sharks and green turtles provides insight into their predator-prey relationship. *PloS one, 7*(12).

Francis, M. P., Holdsworth, J. C., & Block, B. A. (2015). Life in the open ocean: seasonal migration and diel diving behaviour of Southern Hemisphere porbeagle sharks (Lamna nasus). *Marine biology, 162*(11), 2305-2323.

Francis, M. P., Shivji, M. S., Duffy, C. A., Rogers, P. J., Byrne, M. E., Wetherbee, B. M., . . . Meyers, M. M. (2019). Oceanic nomad or coastal resident? Behavioural switching in the shortfin mako shark (Isurus oxyrinchus). *Marine biology, 166*(1), 5.

Gaube, P., Braun, C. D., Lawson, G. L., McGillicuddy, D. J., Della Penna, A., Skomal, G. B., . . . Thorrold, S. R. (2018). Mesoscale eddies influence the movements of mature female white sharks in the Gulf Stream and Sargasso Sea. *Scientific reports, 8*(1), 1-8.

Gauld, J. (1989). *Records of Porbeagles Landed in Scotland: With Observations on the Biology, Distribution and Exploitation of the Species*: Department of Agriculture and Fisheries for Scotland.

Gelsleichter, J., Musick, J. A., & Nichols, S. (1999). Food habits of the smooth dogfish, Mustelus canis, dusky shark, Carcharhinus obscurus, Atlantic sharpnose shark, Rhizoprionodon terraenovae, and the sand tiger, Carcharias taurus, from the northwest Atlantic Ocean. *Environmental Biology of Fishes, 54*(2), 205-217. doi:Doi 10.1023/A:1007527111292

Glaus, K. B. J., Brunnschweiler, J. M., Piovano, S., Mescam, G., Genter, F., Fluekiger, P., & Rico, C. (2019). Essential waters: Young bull sharks in Fiji's largest riverine system. *Ecology and evolution, 9*(13), 7574-7585. doi:10.1002/ece3.5304

Goodman, M. A., Conn, P. B., & Fitzpatrick, E. (2011). Seasonal Occurrence of Cownose Rays (Rhinoptera bonasus) in North Carolina's Estuarine and Coastal Waters. *Estuaries and Coasts, 34*(3), 640-651. doi:10.1007/s12237-010-9355-5

Gore, M. A., Rowat, D., Hall, J., Gell, F. R., & Ormond, R. F. (2008). Transatlantic migration and deep mid-ocean diving by basking shark. *Biology letters, 4*(4), 395-398. doi:10.1098/rsbl.2008.0147

Guttridge, T. L., Bergmann, M. P. M. V., Bolte, C., Howey, L. A., Finger, J. S., Kessel, S. T., . . . Gruber, S. H. (2017). Philopatry and Regional Connectivity of the Great Hammerhead Shark, Sphyrna mokarran in the US and Bahamas. *Frontiers in Marine Science, 4*. doi: 10.3389/fmars.2017.00003

Hammerschlag, N., Bell, I., Fitzpatrick, R., Gallagher, A. J., Hawkes, L. A., Meekan, M. G., . . . Barnett, A. (2016). Behavioral evidence suggests facultative scavenging by a marine apex predator during a food pulse. *Behavioral Ecology and Sociobiology, 70*(10), 1777-1788. doi:10.1007/s00265-016-2183-2

Hammerschlag, N., Broderick, A. C., Coker, J. W., Coyne, M. S., Dodd, M., Frick, M. G., . . . Hartog, K. (2015). Evaluating the landscape of fear between apex predatory sharks and mobile sea turtles across a large dynamic seascape. *Ecology, 96*(8), 2117-2126.

Hammerschlag, N., Gallagher, A. J., Lazarre, D. M., & Slonim, C. (2011). Range extension of the Endangered great hammerhead shark Sphyrna mokarran in the Northwest Atlantic: preliminary data and significance for conservation. *Endangered Species Research, 13*(2), 111-116.

Hammerschlag, N., Gallagher, A. J., Wester, J., Luo, J. G., & Ault, J. S. (2012). Don't bite the hand that feeds: assessing ecological impacts of provisioning ecotourism on an apex marine predator. *Functional Ecology, 26*(3), 567-576. doi:10.1111/j.1365-2435.2012.01973.x

Hammerschlag, N., Luo, J. G., Irschick, D. J., & Ault, J. S. (2012). A Comparison of Spatial and Movement Patterns between Sympatric Predators: Bull Sharks (Carcharhinus leucas) and Atlantic Tarpon (Megalops atlanticus). *PloS one, 7*(9). doi:ARTN e4595810.1371/journal.pone.0045958

Hearn, A. R., Green, J., Román, M., Acuña-Marrero, D., Espinoza, E., & Klimley, A. (2016). Adult female whale sharks make long-distance movements past Darwin Island (Galapagos, Ecuador) in the Eastern Tropical Pacific. *Marine biology, 163*(10), 214.

Heithaus, M. R. (2001). The biology of tiger sharks, Galeocerdo cuvier, in Shark Bay, Western Australia: sex ratio, size distribution, diet, and seasonal changes in catch rates. *Environmental Biology of Fishes, 61*(1), 25-36.

Heithaus, M. R., Wirsing, A. J., Dill, L. M., & Heithaus, L. I. (2007). Long-term movements of tiger sharks satellite-tagged in Shark Bay, Western Australia. *Marine biology, 151*(4), 1455-1461.

Hernandez-Aguilar, S. B., Escobar-Sanchez, O., Galvan-Magana, F., & Abitia-Cardenas, L. (2016). Trophic ecology of the blue shark (Prionace glauca) based on stable isotopes (delta C-13 and delta N-15) and stomach content. *Journal of the Marine Biological Association of the United Kingdom, 96*(7), 1403-1410. doi:10.1017/S0025315415001393

Heupel, M., & Hueter, R. (2002). Importance of prey density in relation to the movement patterns of juvenile blacktip sharks (Carcharhinus limbatus) within a coastal nursery area. *Marine and Freshwater Research, 53*(2), 543-550.

Heupel, M. R., & Simpfendorfer, C. A. (2011). Estuarine nursery areas provide a low-mortality environment for young bull sharks Carcharhinus leucas. *Marine Ecology Progress Series, 433*, 237-244. doi:10.3354/meps09191

Heupel, M. R., Simpfendorfer, C. A., Espinoza, M., Smoothey, A. F., Tobin, A., & Peddemors, V. (2015). Conservation challenges of sharks with continental scale migrations. *Frontiers in Marine Science, 2*. doi:10.3389/fmars.2015.00012

Heyman, W. D., Graham, R. T., Kjerfve, B., & Johannes, R. E. (2001). Whale sharks Rhincodon typus aggregate to feed on fish spawn in Belize. *Marine Ecology Progress Series, 215*, 275-282.

Hoffmayer, E. R., Franks, J. S., Driggers, W. B., McKinney, J. A., Hendon, J. M., & Quattro, J. M. (2014). Habitat, movements and environmental preferences of dusky sharks, Carcharhinus obscurus, in the northern Gulf of Mexico. *Marine biology, 161*(4), 911-924. doi:10.1007/s00227-014-2391-0

Hoffmayer, E. R., & Parsons, G. R. (2003). Food habits of three shark species from the Mississippi Sound in the northern Gulf of Mexico. *Southeastern Naturalist, 2*(2), 271-280. doi:Doi 10.1656/1528-7092(2003)002[0271:Fhotss]2.0.Co;2

Holmes, B. J., Pepperell, J. G., Griffiths, S. P., Jaine, F. R., Tibbetts, I. R., & Bennett, M. B. (2014). Tiger shark (Galeocerdo cuvier) movement patterns and habitat use determined by satellite tagging in eastern Australian waters. *Marine biology, 161*(11), 2645-2658.

Housiaux, J. A., Hepburn, C. D., & Rayment, W. J. (2019). Seasonal variation in occurrence of the sevengill shark, Notorynchus cepedianus, in two inshore habitats of southern New Zealand. *New Zealand Journal of Zoology, 46*(1), 48-60.

Howey, L. A., Wetherbee, B. M., Tolentino, E. R., & Shivji, M. S. (2017). Biogeophysical and physiological processes drive movement patterns in a marine predator. *Movement Ecology, 5*. doi: 10.1186/s40462-017-0107-z

Hsu, H.-H., Joung, S.-J., Liao, Y.-Y., & Liu, K.-M. (2007). Satellite tracking of juvenile whale sharks, Rhincodon typus, in the Northwestern Pacific. *Fisheries Research, 84*(1), 25-31.

Hueter, R. E., Tyminski, J. P., & de la Parra, R. (2013). Horizontal movements, migration patterns, and population structure of whale sharks in the Gulf of Mexico and northwestern Caribbean Sea. *PloS one, 8*(8).

Hulbert, L., Aires‐da‐Silva, A., Gallucci, V., & Rice, J. (2005). Seasonal foraging movements and migratory patterns of female Lamna ditropis tagged in Prince William Sound, Alaska. *Journal of Fish Biology, 67*(2), 490-509.

Hussey, N. E., Dudley, S. F. J., McCarthy, I. D., Cliff, G., & Fisk, A. T. (2011). Stable isotope profiles of large marine predators: viable indicators of trophic position, diet, and movement in sharks? *Canadian Journal of Fisheries and Aquatic Sciences, 68*(12), 2029-2045. doi:10.1139/F2011-115

Hussey, N. E., McCarthy, I. D., Dudley, S. F. J., & Mann, B. Q. (2009). Nursery grounds, movement patterns and growth rates of dusky sharks, Carcharhinus obscurus: a long-term tag and release study in South African waters. *Marine and Freshwater Research, 60*(6), 571-583. doi:10.1071/Mf08280

Jaine, F., Rohner, C., Weeks, S., Couturier, L., Bennett, M., Townsend, K. A., & Richardson, A. (2014). Movements and habitat use of reef manta rays off eastern Australia: offshore excursions, deep diving and eddy affinity revealed by satellite telemetry. *Marine Ecology Progress Series, 510*, 73-86.

Jaureguizar, A. J., Argemi, F., Trobbiani, G., Palma, E. D., & Irigoyen, A. J. (2018). Large-scale migration of a school shark, Galeorhinus galeus, in the Southwestern Atlantic. *Neotropical Ichthyology, 16*(1).

Jorgensen, S. J., Arnoldi, N. S., Estess, E. E., Chapple, T. K., Ruckert, M., Anderson, S. D., & Block, B. A. (2012). Eating or Meeting? Cluster Analysis Reveals Intricacies of White Shark (Carcharodon carcharias) Migration and Offshore Behavior. *PloS one, 7*(10). doi:10.1371/journal.pone.0047819

Jorgensen, S. J., Reeb, C. A., Chapple, T. K., Anderson, S., Perle, C., Van Sommeran, S. R., . . . Block, B. A. (2010). Philopatry and migration of Pacific white sharks. *Proceedings of the Royal Society B-Biological Sciences, 277*(1682), 679-688. doi:10.1098/rspb.2009.1155

Joyce, W., Campana, S., Natanson, L., Kohler, N., Pratt Jr, H., & Jensen, C. (2002). Analysis of stomach contents of the porbeagle shark (Lamna nasus Bonnaterre) in the northwest Atlantic. *ICES Journal of Marine Science, 59*(6), 1263-1269.

Kajiura, S. M., & Tellman, S. L. (2016). Quantification of Massive Seasonal Aggregations of Blacktip Sharks (Carcharhinus limbatus) in Southeast Florida. *PloS one, 11*(3). doi: 10.1371/journal.pone.0150911

Kessel, S. T., Elamin, N. A., Yurkowski, D. J., Chekchak, T., Walter, R. P., Klaus, R., . . . Hussey, N. E. (2017). Conservation of reef manta rays (Manta alfredi) in a UNESCO World Heritage Site: Large-scale island development or sustainable tourism? *PloS one, 12*(10), e0185419.

Ketchum, J. T., Hearn, A., Klimley, A. P., Peñaherrera, C., Espinoza, E., Bessudo, S., . . . Arauz, R. (2014). Inter-island movements of scalloped hammerhead sharks (Sphyrna lewini) and seasonal connectivity in a marine protected area of the eastern tropical Pacific. *Marine biology, 161*(4), 939-951.

King, J., Wetklo, M., Supernault, J., Taguchi, M., Yokawa, K., Sosa-Nishizaki, O., & Withler, R. (2015). Genetic analysis of stock structure of blue shark (Prionace glauca) in the north Pacific ocean. *Fisheries Research, 172*, 181-189.

Kneebone, J., Chisholm, J., & Skomal, G. (2014). Movement patterns of juvenile sand tigers (Carcharias taurus) along the east coast of the USA. *Marine biology, 161*(5), 1149-1163.

Kneebone, J., Chisholm, J., & Skomal, G. B. (2012). Seasonal residency, habitat use, and site fidelity of juvenile sand tiger sharks Carcharias taurus in a Massachusetts estuary. *Marine Ecology Progress Series, 471*, 165-181.

Kohler, N. E., Casey, J. G., & Turner, P. A. (1998). NMFS cooperative shark tagging program, 1962-93: an atlas of shark tag and recapture data. *Marine Fisheries Review, 60*(2), 1-1.

Lea, J. S., Wetherbee, B. M., Queiroz, N., Burnie, N., Aming, C., Sousa, L. L., . . . Sims, D. W. (2015). Repeated, long-distance migrations by a philopatric predator targeting highly contrasting ecosystems. *Scientific reports, 5*, 11202.

Lea, J. S., Wetherbee, B. M., Sousa, L. L., Aming, C., Burnie, N., Humphries, N. E., . . . Shivji, M. S. (2018). Ontogenetic partial migration is associated with environmental drivers and influences fisheries interactions in a marine predator. *ICES Journal of Marine Science, 75*(4), 1383-1392.

Lea, J. S. E., Humphries, N. E., Clarke, C. R., & Sims, D. W. (2015). To Madagascar and back: long-distance, return migration across open ocean by a pregnant female bull shark Carcharhinus leucas. *Journal of Fish Biology, 87*(6), 1313-1321. doi:10.1111/jfb.12805

Lee, K. A., Butcher, P. A., Harcourt, R. G., Patterson, T. A., Peddemors, V. M., Roughan, M., . . . Bradford, R. W. (2021). Oceanographic conditions associated with white shark (Carcharodon carcharias) habitat use along eastern Australia. *Marine Ecology Progress Series, 659*, 143-159. doi:10.3354/meps13572

Lee, K. A., Smoothey, A. F., Harcourt, R. G., Roughan, M., Butcher, P. A., & Peddemors, V. M. (2019). Environmental drivers of abundance and residency of a large migratory shark, Carcharhinus leucas, inshore of a dynamic western boundary current. *Marine Ecology Progress Series, 622*, 121-137. doi:10.3354/meps13052

Lipscombe, R. S., Spaet, J. L. Y., Scott, A., Lam, C. H., Brand, C. P., & Butcher, P. A. (2020). Habitat use and movement patterns of tiger sharks (Galeocerdo cuvier) in eastern Australian waters. *ICES Journal of Marine Science, 77*(7-8), 3127-3137. doi:10.1093/icesjms/fsaa212

Loor‐Andrade, P., Galván‐Magaña, F., Elorriaga‐Verplancken, F. R., Polo‐Silva, C., & Delgado‐Huertas, A. (2015). Population and individual foraging patterns of two hammerhead sharks using carbon and nitrogen stable isotopes. *Rapid Communications in Mass Spectrometry, 29*(9), 821-829.

Lowe, C. G., Wetherbee, B. M., Crow, G. L., & Tester, A. L. (1996). Ontogenetic dietary shifts and feeding behavior of the tiger shark, Galeocerdo cuvier, in Hawaiian waters. *Environmental Biology of Fishes, 47*(2), 203-211.

Lucifora, L. O., García, V. B., Menni, R. C., & Escalante, A. H. (2006). Food habits, selectivity, and foraging modes of the school shark Galeorhinus galeus. *Marine Ecology Progress Series, 315*, 259-270.

Lucifora, L. O., Menni, R. C., & Escalante, A. H. (2002). Reproductive ecology and abundance of the sand tiger shark, Carcharias taurus, from the southwestern Atlantic. *ICES Journal of Marine Science, 59*(3), 553-561.

MacNeill, M. A., Skomal, G. B., & Fisk, A. T. (2005). Stable isotopes from multiple tissues reveal diet switching in sharks. *Marine Ecology Progress Series, 302*, 199-206. doi:DOI 10.3354/meps302199

Marcus, L., Virtue, P., Pethybridge, H. R., Meekan, M. G., Thums, M., & Nichols, P. D. (2016). Intraspecific variability in diet and implied foraging ranges of whale sharks at Ningaloo Reef, Western Australia, from signature fatty acid analysis. *Marine Ecology Progress Series, 554*, 115-128.

Marie, A. D., Miller, C., Cawich, C., Piovano, S., & Rico, C. (2017). Fisheries-independent surveys identify critical habitats for young scalloped hammerhead sharks (Sphyrna lewini) in the Rewa Delta, Fiji. *Scientific reports, 7*. doi: 10.1038/s41598-017-17152-0

Marshall, A., & Bennett, M. (2010). Reproductive ecology of the reef manta ray Manta alfredi in southern Mozambique. *Journal of Fish Biology, 77*(1), 169-190.

Maxwell, S. M., Scales, K. L., Bograd, S. J., Briscoe, D. K., Dewar, H., Hazen, E. L., . . . Crowder, L. B. (2019). Seasonal spatial segregation in blue sharks (Prionace glauca) by sex and size class in the Northeast Pacific Ocean. *Diversity and Distributions, 25*(8), 1304-1317. doi:10.1111/ddi.12941

McAllister, J. D., Barnett, A., Lyle, J. M., & Semmens, J. M. (2015). Examining the functional role of current area closures used for the conservation of an overexploited and highly mobile fishery species. *ICES Journal of Marine Science, 72*(8), 2234-2244. doi:10.1093/icesjms/fsv079

McAllister, J. D., Barnett, A., Lyle, J. M., Stehfest, K. M., & Semmens, J. M. (2018). Examining trends in abundance of an overexploited elasmobranch species in a nursery area closure. *Marine and Freshwater Research, 69*(3), 376-384. doi:10.1071/Mf17130

McMillan, M., Huveneers, C., Semmens, J., & Gillanders, B. (2018). Natural tags reveal populations of conservation dependent school shark use different pupping areas. *Marine Ecology Progress Series, 599*, 147-156.

McMillan, M., Huveneers, C., Semmens, J., & Gillanders, B. (2019). Partial female migration and cool-water migration pathways in an overfished shark. *ICES Journal of Marine Science, 76*(4), 1083-1093.

Meekan, M. G., Bradshaw, C. J., Press, M., McLean, C., Richards, A., Quasnichka, S., & Taylor, J. G. (2006). Population size and structure of whale sharks Rhincodon typus at Ningaloo Reef, Western Australia. *Marine Ecology Progress Series, 319*, 275-285.

Meyer, C. G., Anderson, J. M., Coffey, D. M., Hutchinson, M. R., Royer, M. A., & Holland, K. N. (2018). Habitat geography around Hawaii’s oceanic islands influences tiger shark (Galeocerdo cuvier) spatial behaviour and shark bite risk at ocean recreation sites. *Scientific reports, 8*(1), 1-18.

Meyer, C. G., Papastamatiou, Y. P., & Holland, K. N. (2010). A multiple instrument approach to quantifying the movement patterns and habitat use of tiger (Galeocerdo cuvier) and Galapagos sharks (Carcharhinus galapagensis) at French Frigate Shoals, Hawaii. *Marine biology, 157*(8), 1857-1868.

Nalesso, E., Hearn, A., Sosa-Nishizaki, O., Steiner, T., Antoniou, A., Reid, A., . . . Lara, F. (2019). Movements of scalloped hammerhead sharks (Sphyrna lewini) at Cocos Island, Costa Rica and between oceanic islands in the Eastern Tropical Pacific. *PloS one, 14*(3), e0213741.

Nasby-Lucas, N., Dewar, H., Lam, C. H., Goldman, K. J., & Domeier, M. L. (2009). White Shark Offshore Habitat: A Behavioral and Environmental Characterization of the Eastern Pacific Shared Offshore Foraging Area. *PloS one, 4*(12). doi: 10.1371/journal.pone.0008163

Nasby-Lucas, N., Dewar, H., Sosa-Nishizaki, O., Wilson, C., Hyde, J. R., Vetter, R. D., . . . Sippel, T. (2019). Movements of electronically tagged shortfin mako sharks (Isurus oxyrinchus) in the eastern North Pacific Ocean. *Animal Biotelemetry, 7*(1), 12.

Norman, B. M., Holmberg, J. A., Arzoumanian, Z., Reynolds, S. D., Wilson, R. P., Rob, D., . . . Galvan, B. (2017). Undersea constellations: the global biology of an endangered marine megavertebrate further informed through citizen science. *Bioscience, 67*(12), 1029-1043.

Nosal, A. P., Cartamil, D. P., Ammann, A. J., Bellquist, L. F., Ben-Aderet, N. J., Blincow, K. M., . . . Hastings, P. A. (2021). Triennial migration and philopatry in the critically endangered soupfin shark Galeorhinus galeus. *Journal of applied ecology*. doi:10.1111/1365-2664.13848

Ogburn, M. B., Bangley, C. W., Aguilar, R., Fisher, R. A., Curran, M. C., Webb, S. F., & Hines, A. H. (2018). Migratory connectivity and philopatry of cownose rays Rhinoptera bonasus along the Atlantic coast, USA. *Marine Ecology Progress Series, 602*, 197-211. doi:10.3354/meps12686

Omori, K. L., & Fisher, R. A. (2017). Summer and fall movement of cownose ray, Rhinoptera bonasus, along the east coast of United States observed with pop-up satellite tags. *Environmental Biology of Fishes, 100*(11), 1435-1449. doi:10.1007/s10641-017-0654-6

Otway, N. M., & Ellis, M. T. (2011). Pop-up archival satellite tagging of Carcharias taurus: movements and depth/temperature-related use of south-eastern Australian waters. *Marine and Freshwater Research, 62*(6), 607-620. doi:10.1071/Mf10139

Pade, N. G., Queiroz, N., Humphries, N. E., Witt, M. J., Jones, C. S., Noble, L. R., & Sims, D. W. (2009). First results from satellite-linked archival tagging of porbeagle shark, Lamna nasus: area fidelity, wider-scale movements and plasticity in diel depth changes. *Journal of experimental marine biology and ecology, 370*(1-2), 64-74.

Papastamatiou, Y. P., Meyer, C. G., Carvalho, F., Dale, J. J., Hutchinson, M. R., & Holland, K. N. (2013). Telemetry and random-walk models reveal complex patterns of partial migration in a large marine predator. *Ecology, 94*(11), 2595-2606. doi:10.1890/12-2014.1

Payne, N. L., Meyer, C. G., Smith, J. A., Houghton, J. D., Barnett, A., Holmes, B. J., . . . Coffey, D. M. (2018). Combining abundance and performance data reveals how temperature regulates coastal occurrences and activity of a roaming apex predator. *Global change biology, 24*(5), 1884-1893. Retrieved from https://onlinelibrary.wiley.com/doi/full/10.1111/gcb.14088

Pirog, A., Jaquemet, S., Ravigné, V., Cliff, G., Clua, E., Holmes, B. J., . . . Berggren, P. (2019). Genetic population structure and demography of an apex predator, the tiger shark Galeocerdo cuvier. *Ecology and evolution, 9*(10), 5551-5571.

Prince, E. D., & Goodyear, C. P. (2006). Hypoxia‐based habitat compression of tropical pelagic fishes. *Fisheries Oceanography, 15*(6), 451-464.

Ramírez-Macías, D., Vázquez-Haikin, A., & Vázquez-Juárez, R. (2012). Whale shark Rhincodon typus populations along the west coast of the Gulf of California and implications for management. *Endangered Species Research, 18*(2), 115-128.

Raoult, V., Broadhurst, M. K., Peddemors, V. M., Williamson, J. E., & Gaston, T. F. (2019). Resource use of great hammerhead sharks (Sphyrna mokarran) off eastern Australia. *Journal of Fish Biology, 95*(6), 1430-1440. doi:10.1111/jfb.14160

Rogers, P. J., Huveneers, C., Goldsworthy, S. D., Mitchell, J. G., & Seuront, L. (2013). Broad-scale movements and pelagic habitat of the dusky shark Carcharhinus obscurus off Southern Australia determined using pop-up satellite archival tags. *Fisheries Oceanography, 22*(2), 102-112. doi:10.1111/fog.12009

Rogers, P. J., Huveneers, C., Page, B., Goldsworthy, S. D., Coyne, M., Lowther, A. D., . . . Seuront, L. (2015). Living on the continental shelf edge: habitat use of juvenile shortfin makos Isurus oxyrinchus in the Great Australian Bight, southern Australia. *Fisheries Oceanography, 24*(3), 205-218.

Rogers, P. J., Knuckey, I., Hudson, R. J., Lowther, A. D., & Guida, L. (2017). Post-release survival, movement, and habitat use of school shark Galeorhinus galeus in the Great Australian Bight, southern Australia. *Fisheries Research, 187*, 188-198. doi:10.1016/j.fishres.2016.11.011

Rohner, C., Pierce, S., Marshall, A., Weeks, S., Bennett, M., & Richardson, A. (2013). Trends in sightings and environmental influences on a coastal aggregation of manta rays and whale sharks. *Marine Ecology Progress Series, 482*, 153-168.

Salinas-de-Leon, P., Hoyos-Padilla, E. M., & Pochet, F. (2017). First observation on the mating behaviour of the endangered scalloped hammerhead shark Sphyrna lewini in the Tropical Eastern Pacific. *Environmental Biology of Fishes, 100*(12), 1603-1608. doi:10.1007/s10641-017-0668-0

Saunders, R. A., Royer, F., & Clarke, M. W. (2011). Winter migration and diving behaviour of porbeagle shark, Lamna nasus, in the Northeast Atlantic. *ICES Journal of Marine Science, 68*(1), 166-174.

Simpfendorfer, C. A., & Milward, N. E. (1993). Utilization of a Tropical Bay as a Nursery Area by Sharks of the Families Carcharhinidae and Sphyrnidae. *Environmental Biology of Fishes, 37*(4), 337-345. doi:Doi 10.1007/Bf00005200

Sims, D. W., Southall, E. J., Quayle, V. A., & Fox, A. M. (2000). Annual social behaviour of basking sharks associated with coastal front areas. *Proceedings of the Royal Society B-Biological Sciences, 267*(1455), 1897-1904. doi:DOI 10.1098/rspb.2000.1227

Sims, D. W., Witt, M. J., Richardson, A. J., Southall, E. J., & Metcalfe, J. D. (2006). Encounter success of free-ranging marine predator movements across a dynamic prey landscape. *Proceedings of the Royal Society B-Biological Sciences, 273*(1591), 1195-1201. doi:10.1098/rspb.2005.3444

Skomal, G., Braun, C., Chisholm, J., & Thorrold, S. (2017). Movements of the white shark Carcharodon carcharias in the North Atlantic Ocean. *Marine Ecology Progress Series, 580*, 1-16.

Skomal, G. B., Zeeman, S. I., Chisholm, J. H., Summers, E. L., Walsh, H. J., McMahon, K. W., & Thorrold, S. R. (2009). Transequatorial Migrations by Basking Sharks in the Western Atlantic Ocean. *Current Biology, 19*(12), 1019-1022. doi:10.1016/j.cub.2009.04.019

Smale, M. (2005). The diet of the ragged-tooth shark Carcharias taurus Rafinesque 1810 in the Eastern Cape, South Africa. *African Journal of Marine Science, 27*(1), 331-335.

Smale, M. J. (2002). Occurrence of Carcharias taurus in nursery areas of the Eastern and Western Cape, South Africa. *Marine and Freshwater Research, 53*(2), 551-556. doi:Unsp 1323-1650/02/02055110.1071/Mf01129

Smale, M. J., Booth, A. J., Farquhar, M. R., Meÿer, M. R., & Rochat, L. (2012). Migration and habitat use of formerly captive and wild raggedtooth sharks (Carcharias taurus) on the southeast coast of South Africa. *Marine Biology Research, 8*(2), 115-128.

Smoothey, A. F., Gray, C. A., Kennelly, S. J., Masens, O. J., Peddemors, V. M., & Robinson, W. A. (2016). Patterns of Occurrence of Sharks in Sydney Harbour, a Large Urbanised Estuary. *PloS one, 11*(1). doi:ARTN e014691110.1371/journal.pone.0146911

Smoothey, A. F., Lee, K. A., & Peddemors, V. M. (2019). Long-term patterns of abundance, residency and movements of bull sharks (Carcharhinus leucas) in Sydney Harbour, Australia. *Scientific reports, 9*. doi:ARTN 1886410.1038/s41598-019-54365-x

Spaet, J. L. Y., Manica, A., Brand, C. P., Gallen, C., & Butcher, P. A. (2020a). Environmental conditions are poor predictors of immature white shark Carcharodon carcharias occurrences on coastal beaches of eastern Australia. *Marine Ecology Progress Series, 653*, 167-179. doi:10.3354/meps13488

Spaet, J. L. Y., Patterson, T. A., Bradford, R. W., & Butcher, P. A. (2020b). Spatiotemporal distribution patterns of immature Australasian white sharks (Carcharodon carcharias). *Scientific reports, 10*(1). doi:ARTN 1016910.1038/s41598-020-66876-z

Stehfest, K. M., Patterson, T. A., Barnett, A., & Semmens, J. M. (2014). Intraspecific differences in movement, dive behavior and vertical habitat preferences of a key marine apex predator. *Marine Ecology Progress Series, 495*, 249-262. doi:10.3354/meps10563

Stevens, J. D., Bradford, R. W., & West, G. J. (2010). Satellite tagging of blue sharks (Prionace glauca) and other pelagic sharks off eastern Australia: depth behaviour, temperature experience and movements. *Marine biology, 157*(3), 575-591. doi:10.1007/s00227-009-1343-6

Stevens, J. D., & Lyle, J. M. (1989). Biology of 3 Hammerhead Sharks (Eusphyra-Blochii, Sphyrna-Mokarran and S-Lewini) from Northern Australia. *Australian Journal of Marine and Freshwater Research, 40*(2), 129-146. Retrieved from <Go to ISI>://WOS:A1989AA97500002

Sulikowski, J. A., Wheeler, C. R., Gallagher, A. J., Prohaska, B. K., Langan, J. A., & Hammerschlag, N. (2016). Seasonal and life-stage variation in the reproductive ecology of a marine apex predator, the tiger shark Galeocerdo cuvier, at a protected female-dominated site. *Aquatic Biology, 24*(3), 175-184.

Teter, S. M., Wetherbee, B. M., Fox, D. A., Lam, C. H., Kiefer, D. A., & Shivji, M. (2015). Migratory patterns and habitat use of the sand tiger shark (Carcharias taurus) in the western North Atlantic. *Marine and Freshwater Research, 66*(2), 158-169.

Thorburn, J., Neat, F., Burrett, I., Henry, L.-A., Bailey, D., Jones, C., & Noble, L. (2019). Ontogenetic and seasonal variation in movements and depth use, and evidence of partial migration in a benthopelagic elasmobranch. *Frontiers in Ecology and Evolution, 7*, 353.

Tillett, J., Meekan, M. G., Field, I. C., Thorburn, D. C., & Ovenden, J. R. (2012). Evidence for reproductive philopatry in the bull shark Carcharhinus leucas. *Journal of Fish Biology, 80*(6), 2140-2158. doi:10.1111/j.1095-8649.2012.03228.x

Vandeperre, F., Aires-da-Silva, A., Fontes, J., Santos, M., Santos, R. S., & Afonso, P. (2014). Movements of Blue Sharks (Prionace glauca) across Their Life History. *PloS one, 9*(8). doi:ARTN e10353810.1371/journal.pone.0103538

Vandeperre, F., Aires-da-Silva, A., Lennert-Cody, C., Santos, R. S., & Afonso, P. (2016). Essential pelagic habitat of juvenile blue shark (Prionace glauca) inferred from telemetry data. *Limnology and Oceanography, 61*(5), 1605-1625. doi:10.1002/lno.10321

Vaudo, J. J., Byrne, M. E., Wetherbee, B. M., Harvey, G. M., & Shivji, M. S. (2017). Long‐term satellite tracking reveals region‐specific movements of a large pelagic predator, the shortfin mako shark, in the western North Atlantic Ocean. *Journal of applied ecology, 54*(6), 1765-1775.

Vedor, M., Queiroz, N., Mucientes, G., Couto, A., Costa, I. D., Santos, A. D., . . . Sims, D. W. (2021). Climate-driven deoxygenation elevates fishing vulnerability for the ocean's widest ranging shark. *Elife, 10*. doi:10.7554/eLife.62508

Wells, R., TinHan, T. C., Dance, M. A., Drymon, J. M., Falterman, B., Ajemian, M. J., . . . Driggers III, W. B. (2018). Movement, behavior, and habitat use of a marine apex predator, the scalloped hammerhead. *Frontiers in Marine Science, 5*, 321.

Weng, K. C., Boustany, A. M., Pyle, P., Anderson, S. D., Brown, A., & Block, B. A. (2007). Migration and habitat of white sharks (Carcharodon carcharias) in the eastern Pacific Ocean. *Marine biology, 152*(4), 877-894. doi:10.1007/s00227-007-0739-4

Weng, K. C., Castilho, P. C., Morrissette, J. M., Landeira-Fernandez, A. M., Holts, D. B., Schallert, R. J., . . . Block, B. A. (2005). Satellite tagging and cardiac physiology reveal niche expansion in salmon sharks. *Science, 310*(5745), 104-106.

Weng, K. C., Foley, D. G., Ganong, J. E., Perle, C., Shillinger, G. L., & Block, B. A. (2008). Migration of an upper trophic level predator, the salmon shark Lamna ditropis, between distant ecoregions. *Marine Ecology Progress Series, 372*, 253-264. doi:10.3354/meps07706

Werry, J. M., Lee, S. Y., Otway, N. M., Hu, Y., & Sumpton, W. (2011). A multi-faceted approach for quantifying the estuarine-nearshore transition in the life cycle of the bull shark, Carcharhinus leucas. *Marine and Freshwater Research, 62*(12), 1421-1431. doi:10.1071/Mf11136

Werry, J. M., Planes, S., Berumen, M. L., Lee, K. A., Braun, C. D., & Clua, E. (2014). Reef-fidelity and migration of tiger sharks, Galeocerdo cuvier, across the Coral Sea. *PloS one, 9*(1).

Whitney, N. M., & Crow, G. L. (2007). Reproductive biology of the tiger shark (Galeocerdo cuvier) in Hawaii. *Marine biology, 151*(1), 63-70.

Williams, G., Andrews, K. S., Katz, S., Moser, M. L., Tolimieri, N., Farrer, D., & Levin, P. (2012). Scale and pattern of broadnose sevengill shark Notorynchus cepedianus movement in estuarine embayments. *Journal of Fish Biology, 80*(5), 1380-1400.

Williams, G. D., Andrews, K. S., Farrer, D. A., Bargmann, G. G., & Levin, P. S. (2011). Occurrence and biological characteristics of broadnose sevengill sharks (Notorynchus cepedianus) in Pacific Northwest coastal estuaries. *Environmental Biology of Fishes, 91*(4), 379-388.

Williams, R., Okey, T. A., Wallace, S. S., & Gallucci, V. F. (2010). Shark aggregation in coastal waters of British Columbia. *Marine Ecology Progress Series, 414*, 249-256. doi:10.3354/meps08718

Wilson, S., Polovina, J., Stewart, B., & Meekan, M. (2006). Movements of whale sharks (Rhincodon typus) tagged at Ningaloo Reef, Western Australia. *Marine biology, 148*(5), 1157-1166.

Yates, P. M., Heupel, M. R., Tobin, A. J., & Simpfendorfer, C. A. (2015). Ecological Drivers of Shark Distributions along a Tropical Coastline. *PloS one, 10*(4). doi: 10.1371/journal.pone.0121346
